# Stepwise-coordinated polymeric nanoplatform enables traceless cytosolic protein delivery and autophagy blockade for enhanced tumor therapy

**DOI:** 10.1093/nsr/nwag446

**Published:** 2026-07-20

**Authors:** Peng Zhang, Yaowei Zhu, Hongyu Zhang, Shuai Tang, Zhihui Guo, Xuan Yi, Chunsheng Xiao, Xuesi Chen

**Affiliations:** State Key Laboratory of Polymer Science and Technology, Changchun Institute of Applied Chemistry, Chinese Academy of Sciences, Changchun 130022, China; College of Chemistry and Chemical Engineering, Taishan University, Tai’an 271000, China; State Key Laboratory of Polymer Science and Technology, Changchun Institute of Applied Chemistry, Chinese Academy of Sciences, Changchun 130022, China; State Key Laboratory of Supramolecular Structure and Materials, College of Chemistry, Jilin University, Changchun 130012, China; State Key Laboratory of Polymer Science and Technology, Changchun Institute of Applied Chemistry, Chinese Academy of Sciences, Changchun 130022, China; State Key Laboratory of Polymer Science and Technology, Changchun Institute of Applied Chemistry, Chinese Academy of Sciences, Changchun 130022, China; State Key Laboratory of Polymer Science and Technology, Changchun Institute of Applied Chemistry, Chinese Academy of Sciences, Changchun 130022, China; State Key Laboratory of Polymer Science and Technology, Changchun Institute of Applied Chemistry, Chinese Academy of Sciences, Changchun 130022, China

**Keywords:** polymer nanocarrier, protein drug, protein delivery, autophagy blockade, tumor therapy

## Abstract

Autophagic degradation of proteins following cellular internalization and endosomal escape presents a significant, yet underexplored, challenge in intracellular protein delivery. This highlights the need for delivery carriers capable of concurrently achieving cytosolic delivery and autophagy inhibition. Here, we report a stepwise-coordinated polymeric nanoplatform based on a tailor-made amphiphilic triblock copolymer (mPEG-*b*-PGBA-*b*-PGCQ) that enables traceless cytosolic delivery of functional proteins while concurrently suppressing autophagy. This system incorporates three modular blocks: an mPEG stealth shell, a PGBA block for reversible protein conjugation via pH-sensitive iminoboronates, and a PGCQ block grafted with autophagy inhibitor hydroxychloroquine (HCQ) via self-immolative disulfide linkages. Using ribonuclease A (RNase A) as a model therapeutic, we demonstrated efficient protein release in acidic endolysosomes, simultaneous HCQ-promoted endosomal escape, and glutathione-triggered HCQ liberation in cytosol. The released HCQ significantly enhanced RNase A-induced apoptosis by inhibiting both the autophagic degradation of the protein and RNase A-induced mitophagy. Furthermore, by integrating cytosolic delivery with autophagy inhibition, this platform produced notable functional enhancement across multiple proteins, including green fluorescent protein, β-galactosidase, and horseradish peroxidase, demonstrating its broad utility. Our work establishes a potent traceless delivery and autophagy-blockade strategy for boosting intracellular protein efficacy, with considerable potential in both basic research and biomedical applications.

## Teaser

A smart nanocarrier simultaneously delivers proteins into cells and prevents their intracellular degradation, greatly enhancing protein activity and offering a versatile strategy for protein-based research and therapy.

## INTRODUCTION

Proteins act as pivotal mediators in diverse biological processes, including signal transduction, metabolism, and immune regulation [1,2]. Owing to their high specificity, potency, and biocompatibility, protein-based therapeutics have been widely developed to treat diseases such as diabetes, cancer, and autoimmune disorders [3,4]. However, their clinical translation remains largely confined to extracellular targets, primarily hindered by two key barriers: the plasma membrane, which blocks cellular entry, and the endosomal barrier, which traps internalized proteins and facilitates their degradation, thereby preventing cytosolic access [5–7]. To address these challenges, various delivery vehicles, including cell-penetrating peptides [8–11], polymers [12–18], lipids [19–21], and inorganic nanoparticles [22–26], have been engineered to facilitate cytosolic protein delivery. Despite progress in enhancing cellular uptake and endosomal escape, these systems frequently fail to protect proteins from degradation by cellular self-detoxification mechanisms like autophagy—a critical and underexplored obstacle in the post-endosomal escape phase [27–30].

As a conserved self-degradative process, autophagy facilitates the encapsulation of proteins and damaged organelles into double-membraned autophagosomes for subsequent lysosomal degradation [27,28]. Although autophagy plays context-dependent and at times contradictory roles in tumor biology, it is well established that cancer cells frequently upregulate autophagic activity to mitigate apoptosis triggered by therapeutic agents, including protein-based drugs [29–31]. Consequently, even after successful endosomal escape, delivered proteins remain vulnerable to degradation by upregulated autophagy in the cytosol, which significantly compromises their therapeutic efficacy. In this context, autophagy inhibition represents a promising strategy to augment the potency of protein therapeutics. However, to the best of our knowledge, the development of polymeric nanocarriers capable of concurrently enabling cytosolic protein delivery and intrinsic autophagy suppression has not yet been reported.

Herein, we present a stepwise-coordinated nanoplatform constructed from a tailor-made amphiphilic triblock copolymer (mPEG-*b*-PGBA-*b*-PGCQ), which enables traceless cytosolic protein delivery and concurrent autophagy inhibition for enhanced antitumor therapy (Scheme 1). The copolymer consists of three functionally distinct blocks: an mPEG segment that serves as a stealth shell, a middle PGBA block designed to conjugate proteins through pH-reversible iminoboronates [32–36], and a terminal PGCQ block incorporating hydroxychloroquine (HCQ)—a known autophagy inhibitor—via self-immolative disulfide linkages [37–39]. Ribonuclease A (RNase A), which triggers apoptosis through cytosolic RNA degradation [40,41], was employed as a model therapeutic protein. The platform was constructed by first self-assembling protein-free micelles under physiological pH, followed by conjugation of RNase A to the PGBA block via iminoboronates in aqueous solution, yielding cross-linked nanoconstructs with enhanced stability, prolonged blood circulation, and improved tumor accumulation. Upon cellular uptake, the acidic endolysosomal environment promoted traceless release of RNase A through cleavage of the iminoboronate bonds. Concurrently, protonation of tertiary amines in the HCQ pendants facilitated endolysosomal escape of both the protein and the copolymer [42,43]. Subsequent cleavage of the disulfide linkers by cytosolic glutathione (GSH) liberated free HCQ. As validated both *in vitro* and *in vivo*, HCQ potentiated RNase A-induced apoptosis by inhibiting autophagic degradation of the protein. Mechanistic studies further revealed that this enhancement was concurrently driven by HCQ-mediated suppression of RNase A-induced mitophagy. Beyond RNase A, the synergistic strategy of combining cytosolic delivery with autophagy inhibition produced significant functional enhancement across multiple proteins, including green fluorescent protein (GFP), β-galactosidase (β-Gal), and horseradish peroxidase (HRP), demonstrating the generalizability of this mechanism for boosting intracellular protein efficacy.

**Scheme 1.**
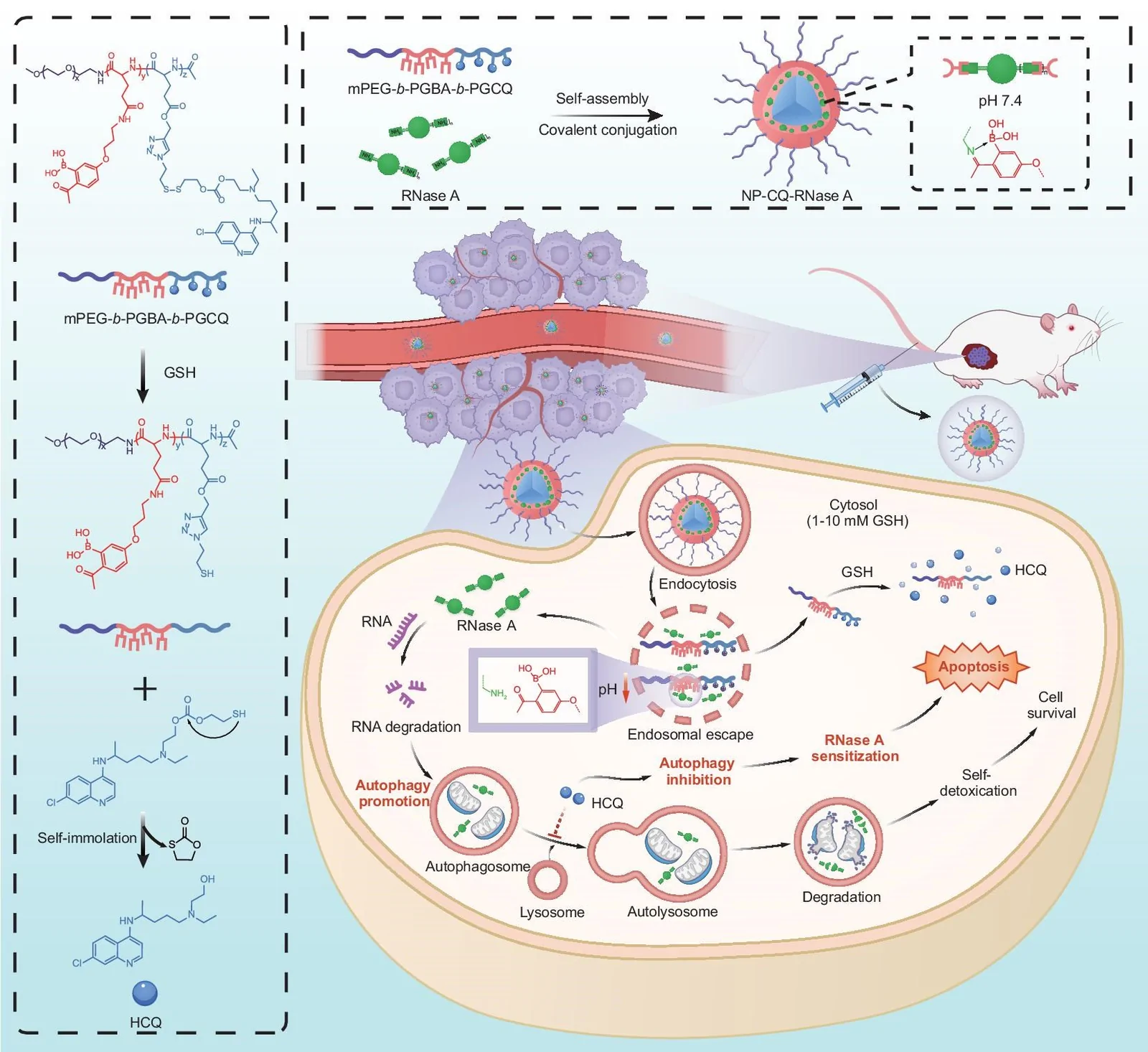
Schematic illustration of the preparation of NP-CQ-RNase A, the cytosolic delivery of Ribonuclease A (RNase A) and HCQ, and the enhanced apoptosis induction of RNase A by HCQ-mediated autophagy inhibition.

## RESULTS AND DISCUSSION

### Preparation and characterization of NP-CQ-RNase A

The preparation of mPEG-*b*-PGBA-*b*-PGCQ was initiated through the sequential ring-opening polymerization (ROP) of BLG-NCA and PLG-NCA, using mPEG-NH_2_ as a macroinitiator, to afford mPEG-*b*-PBLG-*b*-PPLG. The terminal amine group was subsequently blocked with acetic anhydride (Fig. S1). ^1^H nuclear magnetic resonance (NMR) analysis confirmed the degrees of polymerization for the PBLG and PPLG segments to be 25 and 20, respectively (Fig. S2). Subsequent removal of the benzyl groups yielded mPEG-*b*-PLGA-*b*-PPLG (Figs S1 and S3). In parallel, an HCQ derivative containing disulfide and azido (HCQ-SS-N_3_) and a 2-acetylphenylboronic acid (2-APBA) derivative containing primary amine (2-APBA-NH_2_) were synthesized (Figs S4–S14). mPEG-*b*-PLGA-*b*-PGCQ was then obtained by conjugating HCQ-SS-N_3_ onto the side chains of PPLG segment via Cu(I)-catalyzed click reactions (Figs S15 and S16). The appearance of proton peaks a, b, c, d, e and t indicated the successful conjugation of HCQ-SS-N_3_ to PPLG block. Finally, 2-APBA-NH_2_ molecules were grafted onto the side chains of PLGA segment through amidation reactions to achieve mPEG-*b*-PGBA-*b*-PGCQ (Figs S15 and S17). The appearance of proton peaks b’, c’ and d’ verified the successful conjugation of 2-APBA-NH_2_ to PLGA block. The number of 2-APBA-NH_2_ and HCQ-SS-N_3_ introduced to mPEG-*b*-PGBA-*b*-PGCQ was determined to be 25 and 20, respectively (Figs S16 and S17). For comparison, a control copolymer, mPEG-*b*-PGBA-*b*-PGHQ, was synthesized by replacing HCQ with a chlorine-deficient analog 4-(*N*-ethyl-*N*-(2-hydroxyethyl)amino)-1-methylbutylamino)-quinoline (HQ) that lacks autophagy-inhibitory activity [31]. Specifically, HQ was first synthesized via catalytic dechloridation of HCQ using Pd/C under a H_2_ atmosphere (Figs S18 and S19). Then, an HQ derivative containing disulfide and azido (HQ-SS-N_3_) was synthesized (Figs S20–S22). Afterwards, mPEG-*b*-PGBA-*b*-PGHQ was prepared by sequential conjugation of HQ-SS-N_3_ and 2-APBA-NH_2_ to the PPLG and PLGA block of mPEG-*b*-PLGA-*b*-PPLG, respectively (Figs S23–S25). The number of 2-APBA-NH_2_ and HQ-SS-N_3_ that were introduced to mPEG-*b*-PGBA-*b*-PGHQ was also determined to be 25 and 20, respectively (Figs S24 and S25).

Subsequently, the RNase A-loaded autophagy-inhibiting nanoparticles (NP-CQ-RNase A) were constructed via a two-step method. Firstly, the protein-free micelles (NP-CQ) were obtained by the self-assembly of mPEG-*b*-PGBA-*b*-PGCQ. RNase A was then attached to NP-CQ in aqueous solution by the formation of reversible iminoboronates between primary amines on RNase A surfaces and 2-APBA in mPEG-*b*-PGBA-*b*-PGCQ to achieve NP-CQ-RNase A (Fig. 1a). The reversible iminoboronate linkage between polymer and RNase A was verified utilizing 2-APBA and lysine as model agents, analyzed by ^11^B NMR and ^1^H NMR (Fig. 1b and c). In the ^11^B NMR spectrum, 2-APBA alone exhibited a characteristic peak near 30 ppm. Upon mixing with lysine, a new signal emerged around 7 ppm, corresponding to the anionic boron in the iminoboronates. A similar peak was observed when 2-APBA was incubated with RNase A, confirming iminoboronate formation. Notably, this peak disappeared under acidic conditions, demonstrating the pH-sensitive nature of the iminoboronates (Fig. 1b). Furthermore, ^1^H NMR analysis revealed clustering of the aromatic proton signals of 2-APBA following reaction with lysine, which is also indicative of iminoboronate formation (Fig. 1c). This spectral change was also reversed at acidic pH. Together, these results confirm both the feasibility of conjugating RNase A to NP-CQ via iminoboronates and the acid-labile behavior of the linkage. The drug loading content (DLC) was 16.1% for HCQ and 32.9% for RNase A in the NP-CQ-RNase A formulation, with a corresponding drug loading efficiency (DLE) of 98.1% for RNase A. The hydrodynamic diameter of NP-CQ-RNase A was around 126 nm as determined by dynamic light scattering (DLS) measurements (Fig. 1d). Transmission electron microscopy (TEM) images showed that NP-CQ-RNase A had a spherical morphology (Fig. 1d). Furthermore, the nanoparticles (NP-HQ) formed by the self-assembly of mPEG-*b*-PGBA-*b*-PGHQ and the RNase A-loaded NP-HQ (NP-HQ-RNase A) were also prepared by a similar method. NP-CQ, NP-HQ and NP-HQ-RNase A showed a similar diameter around 120 nm (Fig. S26). All nanoparticle variants showed narrow size distributions and negatively charged surfaces (Fig. S27). Notably, the zeta potential values of NP-HQ-RNase A and NP-CQ-RNase A were higher than those of their protein-free counterparts, likely owing to the presence of residual protonatable amines on surface-conjugated RNase A at pH 7.4. This also corroborates the successful surface attachment of RNase A. Moreover, RNase A-mediated surface cross-linking contributed to nanoparticle stabilization. The stability of NP-CQ-RNase A was thus assessed via monitoring particle sizes over 24 h in phosphate-buffered saline (PBS) or PBS supplemented with 10% fetal bovine serum (FBS). Negligible size changes were observed in PBS, and only a slight, non-significant increase occurred in the presence of 10% FBS (Fig. S28), confirming that NP-CQ-RNase A possesses excellent stability suitable for *in vivo* applications.

**Figure 1.**
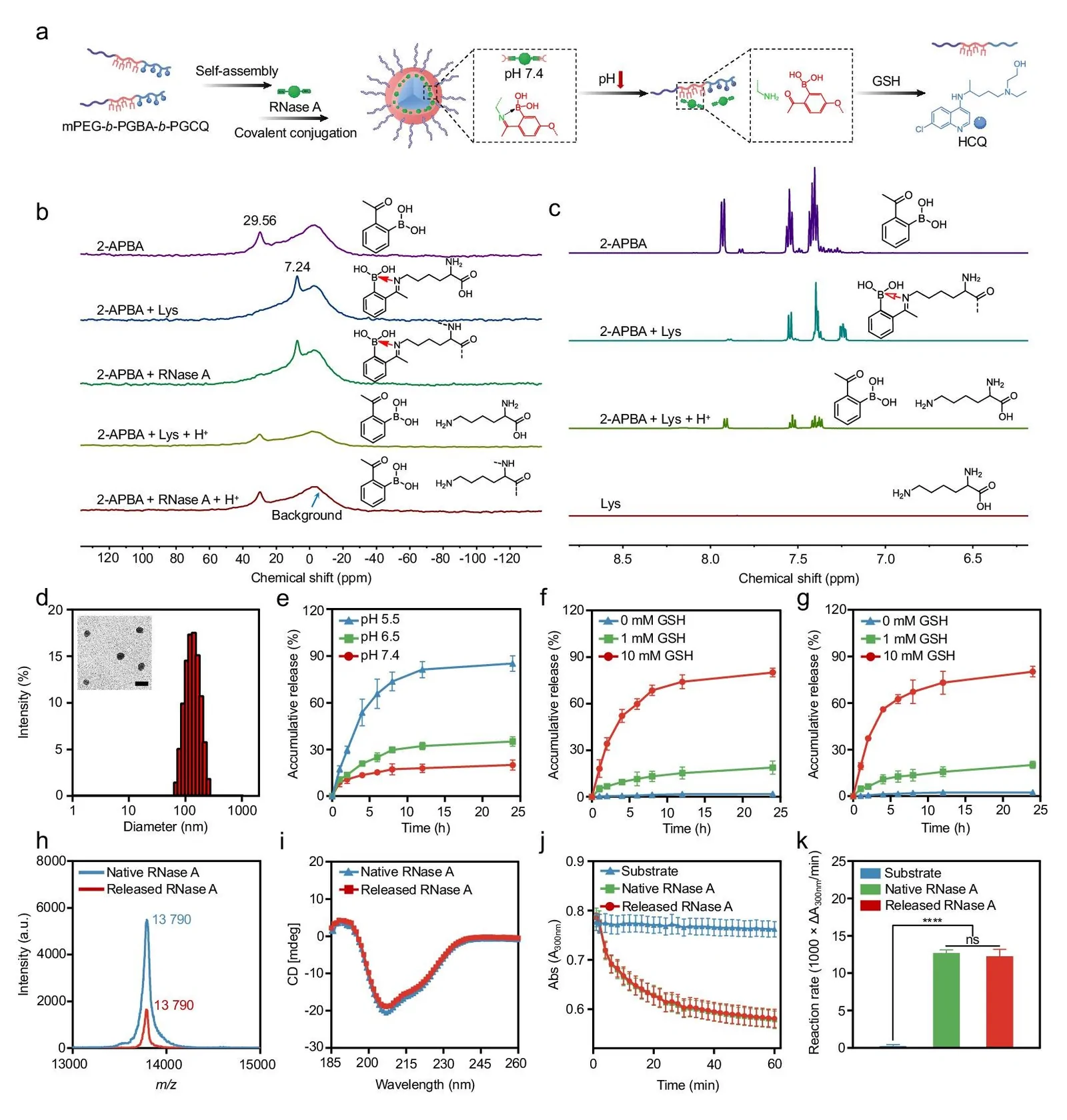
Characterization of NP-CQ-RNase A. (a) Schematic illustration of the fabrication and drug release of NP-CQ-RNase A. (b) ^11^B NMR spectra of 2-APBA, and its mixtures with lysine (1:1 molar ratio) or with RNase A (equimolar to primary amines of RNase A) at pH 7.4 and 4.0. (c) ^1^H NMR spectra of 2-APBA and its mixtures with lysine (Lys, 1:1 molar ratio) at pH 7.4 and 4.0. (d) Characterization of NP-CQ-RNase A at pH 7.4 by DLS and TEM. Scale bar = 200 nm. (e) Release of RNase A-Cy5 from NP-CQ-RNase A-Cy5 in PBS at different pH values (7.4, 6.5, 5.5) at 37°C. (f) HCQ release from NP-CQ-RNase A in PBS (pH 7.4) supplemented with 0, 1, or 10 mM GSH at 37°C. (g) Release kinetics of HQ from NP-HQ-RNase A in PBS (pH 7.4) containing 0, 1, or 10 mM GSH at 37°C. (h) MALDI-TOF MS comparing RNase A released from NP-CQ-RNase A and native RNase A. (i) CD spectra of native RNase A and the released RNase A from NP-CQ-RNase A. (j) Enzymatic activity assay of released RNase A versus native RNase A. (k) Catalytic rates of native and released RNase A, determined by monitoring the absorbance decay at 300 nm for 6 min upon incubation with RNA. ****P < 0.0001, ns: not significant. Data are presented as mean ± SD (*n* = 3).

As previously noted, iminoboronate linkages are cleavable under acidic conditions, and tertiary amines in the PGCQ segment undergo protonation, collectively contributing to pH-responsive protein release, increased hydrophilicity of the polymers, and eventual nanoparticle disassembly. To characterize these changes, the size and morphology of NP-CQ-RNase A were examined at pH 6.5 and 5.5 using DLS and TEM. At pH 6.5, the nanoparticles maintained a spherical morphology despite swelling from 126 nm (pH 7.4) to 251 nm (Fig. S29A). In contrast, at pH 5.5, NP-CQ-RNase A underwent complete disassembly, losing their spherical structure and forming irregular aggregates (Fig. S29B). These findings indicate that the nanoparticles can retain their structural integrity in weakly acidic intratumoral environment (pH ∼6.5) but rapidly dissociate in more acidic endolysosomes (pH ∼5.5), facilitating cargo release. The pH-dependent release profile of RNase A from NP-CQ-RNase A was thus evaluated in PBS at pH 7.4, 6.5, and 5.5. RNase A was released in a sustained and controlled manner over 24 h under all conditions, with the release rate increasing as the pH decreased from 7.4 to 5.5 (Fig. 1e). This behavior is consistent with acid-triggered cleavage of iminoboronates. In addition, HCQ and HQ are expected to be released under reducing conditions due to their linkages to polymers by disulfide bonds. Since cytosolic GSH concentrations typically range from 1 to 10 mM [44,45], the release of HCQ and HQ was evaluated in PBS containing 0, 1, or 10 mM GSH. As shown in Fig. 1f and g, negligible release occurred in the absence of GSH, whereas a significant increase in release was observed with rising GSH concentrations, confirming the reducibility-responsive release behavior mediated by disulfide cleavage. Collectively, these results demonstrate that NP-CQ-RNase A maintains high stability under physiological conditions, minimizing premature cargo leakage, while enabling efficient drug release in acidic and reducing intracellular environments.

Traceless cleavage of covalent linkages is crucial for efficiently releasing native proteins and maintaining their intact bioactivity. To confirm the traceless RNase A release from NP-CQ-RNase A, the molecular weight of released protein was investigated by matrix-assisted laser desorption ionization-time of flight mass spectrometry (MALDI-TOF MS). As shown in Fig. 1h, the released RNase A exhibited a molecular weight of 13 790 Da, identical to that of the native protein, demonstrating its successful release from NP-CQ-RNase A in a traceless form. Circular dichroism spectroscopy further revealed no significant structural alterations in the released RNase A compared to its native counterpart (Fig. 1i), suggesting preservation of its secondary structure. To assess whether the conformational integrity translated to functional activity, the enzymatic performance of the released RNase A was evaluated. As depicted in Fig. 1j and k, both the RNA degradation activity and the catalytic rate of the released RNase A were comparable to those of the native enzyme. Together, these data demonstrate that NP-CQ-RNase A enables traceless release of RNase A without compromising its structural or functional properties.

### *In vitro* autophagy-inhibiting effects and antitumor activity of NP-CQ-RNase A

The cellular internalization of NP-CQ-RNase A by mouse breast cancer (4T1) cells was initially examined using flow cytometry. As shown in Fig. S30, cells treated with NP-CQ-RNase A-Cy5 exhibited a time-dependent increase in fluorescence intensity, indicating efficient cellular uptake. NP-HQ-RNase A-Cy5 displayed comparable uptake kinetics, which can be attributed to their similar hydrodynamic sizes and surface charges (Figs S26 and S27). To elucidate the specific endocytic pathways involved, cells were pretreated for 1 h at either 4°C (to suppress cellular metabolism and arrest energy-dependent processes) or with various endocytosis inhibitors, including amiloride (macropinocytosis), chlorpromazine (clathrin-mediated endocytosis), nystatin (caveolae-mediated endocytosis), and NaN_3_ (energy-dependent endocytosis). Subsequently, cells were incubated with NP-CQ-RNase A-Cy5 for 3 h at 4°C or 37°C. As depicted in Fig. 2a, low-temperature treatment and NaN_3_ significantly diminished intracellular fluorescence, confirming that the internalization of NP-CQ-RNase A is an energy-dependent process. Furthermore, chlorpromazine pretreatment substantially reduced cellular fluorescence compared to the inhibitor-free control, suggesting that clathrin-mediated endocytosis is a predominant pathway for NP-CQ-RNase A uptake. Subsequently, endolysosomal escape was assessed by treating 4T1 cells with different formulations and staining endolysosomes with LysoTracker Green and nuclei with Hoechst 33342. In RNase A-Cy5 group, minimal red fluorescence was detected, confirming the poor cellular permeability of free RNase A (Fig. 2b). In contrast, cells exposed to NP-HQ-RNase A-Cy5 or NP-CQ-RNase A-Cy5 exhibited strong intracellular red fluorescence with limited overlap with green endolysosomal signals, suggesting effective nanoparticle-mediated delivery and subsequent escape of RNase A into the cytosol. This escape can be attributed to tertiary-amine-triggered ‘proton sponge’ effects, which disrupts endolysosomal integrity [42]. Moreover, the NP-HQ-RNase A and NP-CQ-RNase A groups exhibited comparable Pearson correlation coefficients, indicating their similar endosomal escape efficiency (Fig. S31). To further corroborate lysosomal membrane damage, a galectin-3 (Gal3) puncta formation assay was employed. Gal3 redistributes from a diffuse cytoplasmic localization to form puncta upon lysosomal membrane permeabilization (Fig. 2c) [46]. As shown in Fig. 2d, cells treated with NP-HQ, NP-CQ, NP-HQ-RNase A, or NP-CQ-RNase A displayed significant Gal3 puncta compared to the PBS control, confirming the effective lysosomal damage induced by the protonation of tertiary-amines in the polymers. The similar puncta counts across these groups further indicated comparable endosomal escape efficiency (Fig. 2e).

**Figure 2.**
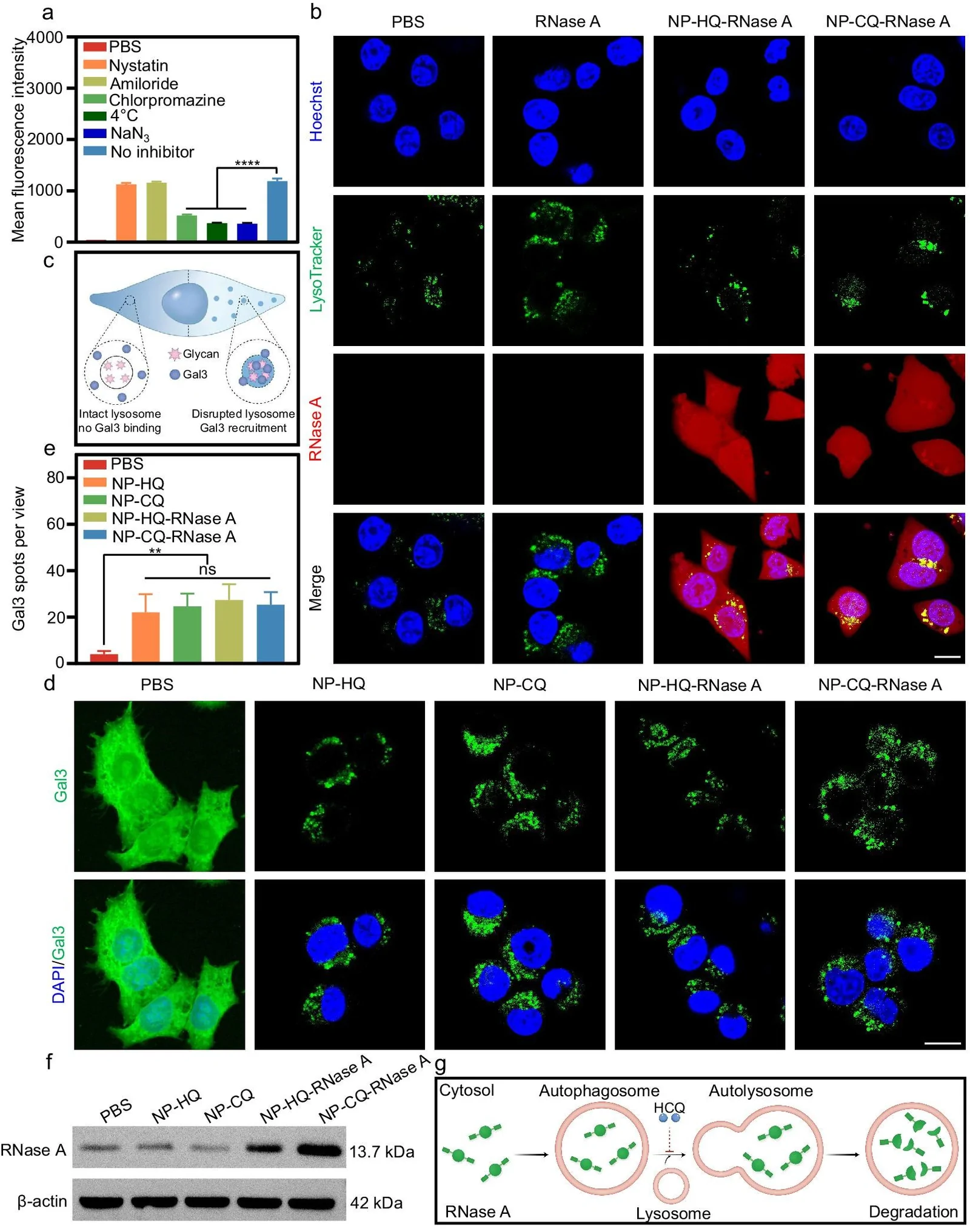
Cytosolic delivery of RNase A. (a) Flow cytometric analysis of mean fluorescence intensities in 4T1 cells pre-incubated either at 4°C or with nystatin (15 μg/mL), chlorpromazine (15 μg/mL), amiloride (10 μg/mL), or NaN_3_ (100 mM) for 1 h, followed by treatment with NP-CQ-RNase A-Cy5 (10 μg/mL RNase A) at 4°C or 37°C for 3 h. (b) Fluorescence micrographs of 4T1 cells exposed to different formulations (10 μg/mL RNase A) at 37°C for 8 h. Endolysosomes and nuclei were labeled with LysoTracker Green (200 nM, 1 h) and Hoechst 33342 (1 μg/mL, 10 min), respectively. Scale bar = 10 μm. (c) Schematic representation of the Gal3 recruitment assay. (d) Assessment of lysosomal membrane integrity via Gal3 (green) and DAPI (blue) staining in 4T1 cells after 8 h treatment with various formulations (10 μg/mL RNase A). Scale bar = 10 μm. (e) Quantitative analysis of Gal3 puncta formation from (d). (f) Western blot analysis of RNase A and β-actin levels in 4T1 cells subjected to the indicated formulations (10 μg/mL RNase A) for 24 h. (g) Schematic of the autophagic degradation of RNase A. **P < 0.01, ****P < 0.0001, ns: not significant. Data represent mean ± SD (*n* = 3).

Furthermore, western blot analysis was performed to evaluate the autophagic degradation of RNase A following endosomal escape. As shown in Fig. 2f, treatment with NP-HQ-RNase A and NP-CQ-RNase A resulted in higher intracellular RNase A levels compared to other groups, confirming successful intracellular delivery of the protein. Notably, the NP-CQ-RNase A group exhibited a significantly higher RNase A level than the NP-HQ-RNase A group. Given that both formulations demonstrated comparable cellular uptake and endosomal escape efficiency (Figs S30 and S31), the enhanced RNase A retention in the NP-CQ-RNase A group likely results from HCQ-mediated inhibition of autophagic degradation (Fig. 2g). To verify this, we evaluated the ability of NP-CQ-RNase A to inhibit autophagy in 4T1 cells. Autophagic flux involves the conversion of LC3-I to LC3-II and its incorporation into autophagosomes, followed by lysosomal degradation of LC3-II and substrates such as p62 (Fig. 3a). HCQ inhibits autophagy by preventing autophagosome-lysosome fusion, resulting in accumulation of autophagosomes, LC3-II and p62 (Fig. 3a). To monitor autophagosome-associated punctum formation and/or accumulation following various treatments, immunofluorescence staining of LC3 was initially performed. Fig. 3b and Fig. S32 depict that cells treated with NP-HQ or PBS exhibited few red fluorescent dots, suggesting a minimal impact of NP-HQ on autophagy. In contrast, treatments with NP-CQ, NP-HQ-RNase A, and NP-CQ-RNase A led to a marked increase in autophagosome-associated puncta compared to PBS and NP-HQ, with the NP-CQ-RNase A group displaying the highest number, indicating a substantial influence on autophagic flux. Furthermore, bio-TEM was employed to directly visualize autophagosome formation and accumulation (Fig. 3c). In comparison to PBS and NP-HQ groups, NP-CQ, NP-HQ-RNase A, and NP-CQ-RNase A treatments resulted in a greater abundance of autophagosomes, with the NP-CQ-RNase A group showing the most pronounced accumulation. These observations are consistent with the immunofluorescence results and collectively demonstrate that these treatments promote the formation and/or accumulation of autophagosomes.

**Figure 3.**
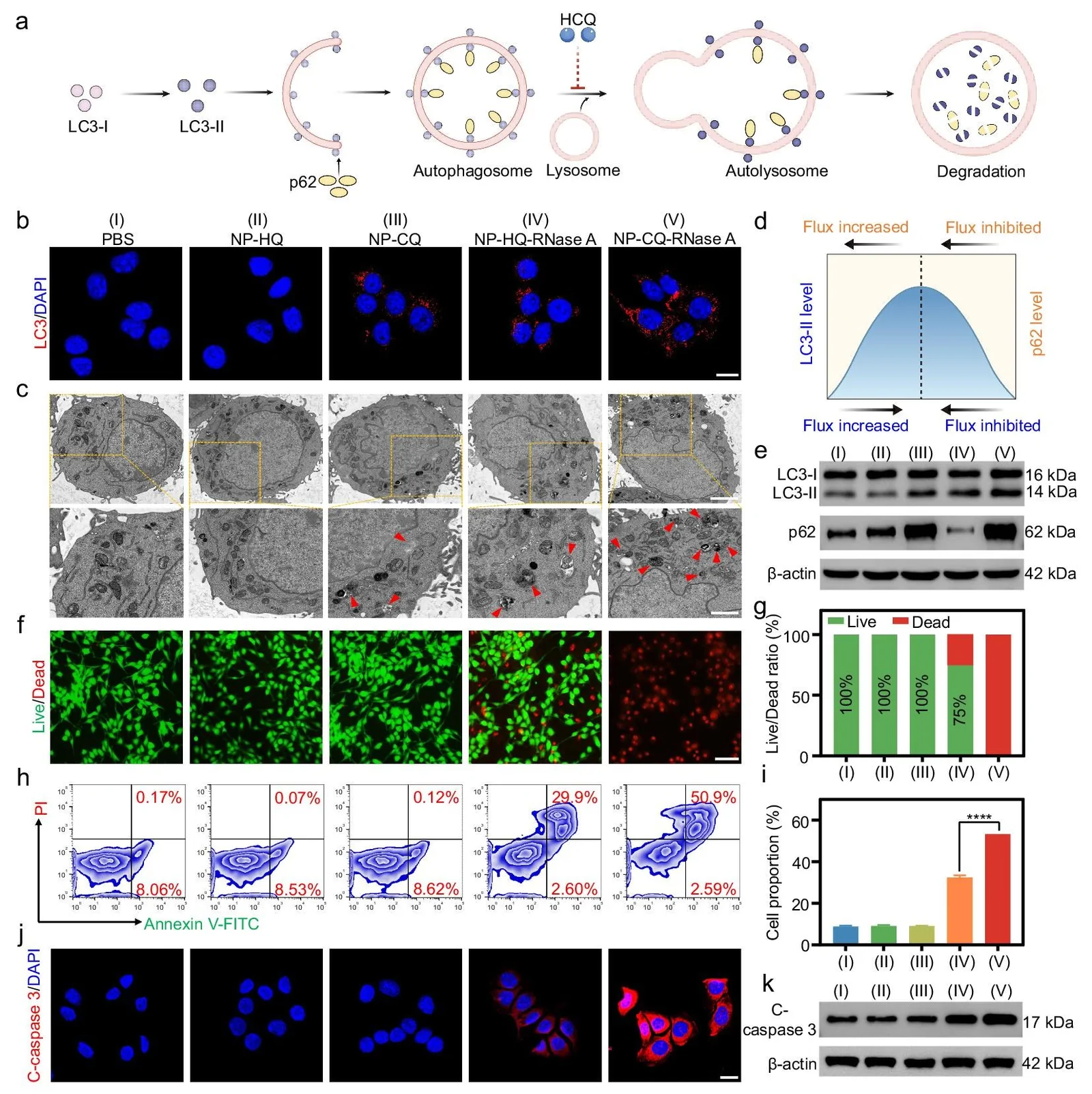
Autophagy suppression and cytotoxicity induced by NP-CQ-RNase A. (a) Diagram illustrating the process of autophagic flux. (b) Representative immunofluorescence micrographs of LC3 puncta in 4T1 cells exposed to the indicated formulations (10 μg/mL RNase A) for 24 h. Scale bar = 10 μm. (c) TEM images of autophagosomes in 4T1 cells following 24 h treatments (10 μg/mL RNase A). Red arrows indicate autophagosomes. Top row, scale bar = 2 μm, bottom row, scale bar = 1 μm. (d) Schematic representation of LC3-II and p62 level dynamics during autophagic flux. (e) Western blot analysis of LC3-I, LC3-II, p62, and β-actin protein expression in 4T1 cells treated with the indicated formulations (10 μg/mL RNase A) for 24 h. (f) Viability assessment of 4T1 cells using Live/Dead staining after 24 h treatment with specified formulations (10 μg/mL RNase A). Scale bar = 50 μm. (g) Quantitative analysis of cell viability based on the results shown in panel (f). (h) Apoptosis evaluation by flow cytometry with Annexin V-FITC/PI double staining in 4T1 cells after 24 h treatments (10 μg/mL RNase A). (i) Proportion of apoptotic cells quantified from the data in panel (h). ****P < 0.0001. Data represent mean ± SD (*n* = 3). (j) Immunofluorescence detection of C-caspase-3 in 4T1 cells following 24 h treatments (10 μg/mL RNase A). Scale bar = 10 μm. (k) Western blot analysis of C-caspase-3 and β-actin levels in 4T1 cells subjected to the indicated formulations (10 μg/mL RNase A) for 24 h.

Nevertheless, the aforementioned results remain insufficient to definitively conclude whether autophagy is activated or suppressed under the specific experimental conditions. An increase in autophagosome number may result from either enhanced autophagic activity or impaired autolysosome formation or function [47]. More detailed molecular evidence is required to accurately distinguish between induction, inhibition, or a combination of both in autophagic regulation. Therefore, we evaluated the protein levels of the autophagy marker LC3-II and the selective substrate p62 by western blot following various treatments. As shown in Fig. 3d and e, NP-HQ treatment led to only marginal increases in LC3-II and p62 levels compared with the PBS group, indicating a limited impact on autophagy. In contrast, NP-CQ, NP-HQ-RNase A, and NP-CQ-RNase A treatments all upregulated LC3-II protein levels relative to the PBS control, with the highest level observed in the NP-CQ-RNase A group. This finding aligns with the increased autophagosome quantity detected by immunofluorescence and bio-TEM. Moreover, p62 abundance was elevated in the NP-CQ and NP-CQ-RNase A groups compared to the PBS group, confirming that released HCQ inhibits autophagosome-lysosome fusion, resulting in accumulation of autophagic components and substrates. In comparison, NP-HQ-RNase A treatment resulted in a notable reduction in p62 protein relative to the PBS group, suggesting that RNase A delivery induces protective autophagy. Collectively, the increased autophagosome count and elevated levels of LC3-II and p62 in NP-CQ-RNase A group, as compared to NP-HQ-RNase A, can be attributed to autophagy inhibition mediated by released HCQ.

These immunoblot data provide solid evidence to differentiate between promotion and inhibition of autophagic flux: while RNase A enhances overall autophagy flux, HCQ prevents autophagosome-lysosome fusion and lysosomal degradation. The synergy between RNase A and HCQ enhances early-phase autophagosome processing but disrupts late-phase lysosomal function, thereby preventing the clearance of RNase A and following restoration of homeostasis. This mechanism is likely to amplify the cell-killing efficacy of RNase A. To verify this, the tumor cell-killing efficacy of NP-CQ-RNase A was evaluated using an MTT assay following 72 h of treatment. Free RNase A showed no cytotoxicity due to poor membrane permeability (Fig. S33). HQ, HCQ, NP-HQ and NP-CQ caused negligible cell death (Figs S34 and S35). In contrast, both NP-HQ-RNase A and NP-CQ-RNase A induced concentration-dependent cytotoxicity, with NP-CQ-RNase A exhibiting superior potency, attributable to its enhanced autophagy inhibition (Fig. S35). Furthermore, the combination of HCQ with NP-HQ-RNase A exhibited higher cytotoxicity than NP-HQ-RNase A alone, reaching a level similar to NP-CQ-RNase A, thereby further confirming that autophagy inhibition plays a critical role in enhancing the therapeutic efficacy of RNase A (Fig. S36). Live/dead staining further confirmed the strongest cytotoxic effect in the NP-CQ-RNase A group, which displayed the highest proportion of dead cells (Fig. 3f and g). Apoptosis-inducing ability of NP-CQ-RNase A was then assessed using Annexin V-FITC/PI staining. While NP-HQ and NP-CQ alone induced minimal apoptosis, both NP-HQ-RNase A and NP-CQ-RNase A triggered significant apoptotic cell death. The apoptosis rate in the NP-CQ-RNase A group (53.5%) was substantially higher than that in the NP-HQ-RNase A group (32.5%) (Fig. 3h and i). Consistent with this, expression of cleaved caspase-3 (C-caspase-3), a key apoptosis executor, was upregulated only in NP-HQ-RNase A and NP-CQ-RNase A groups, as demonstrated by both immunofluorescence staining and western blot assays, with the highest level observed in the latter (Fig. 3j and k). Additional supporting data are shown in Fig. S37. In summary, these findings demonstrate that NP-CQ-RNase A enters cells via clathrin-mediated endocytosis, escapes endolysosomes, and inhibits the autophagic degradation of RNase A, thereby augmenting RNase A-induced apoptosis.

### RNA-seq analysis of 4T1 cells after treatment with NP-CQ-RNase A

To further elucidate the anticancer mechanism of NP-CQ-RNase A, RNA sequencing (RNA-seq) was performed on 4T1 cells to assess its impact on global gene expression. Pearson correlation coefficients between samples within the same group all exceeded 0.94, confirming high reproducibility of the data (Fig. S38). Cluster analysis and heatmap visualization of differentially expressed genes (DEGs) demonstrated distinct transcriptional alterations in response to NP-CQ-RNase A relative to the control (Fig. 4a). Under the thresholds of |fold change| >2 and false discovery rate (FDR) <0.05, a total of 2065 upregulated and 1619 downregulated DEGs were identified in the NP-CQ-RNase A-treated group (Fig. 4b).

**Figure 4.**
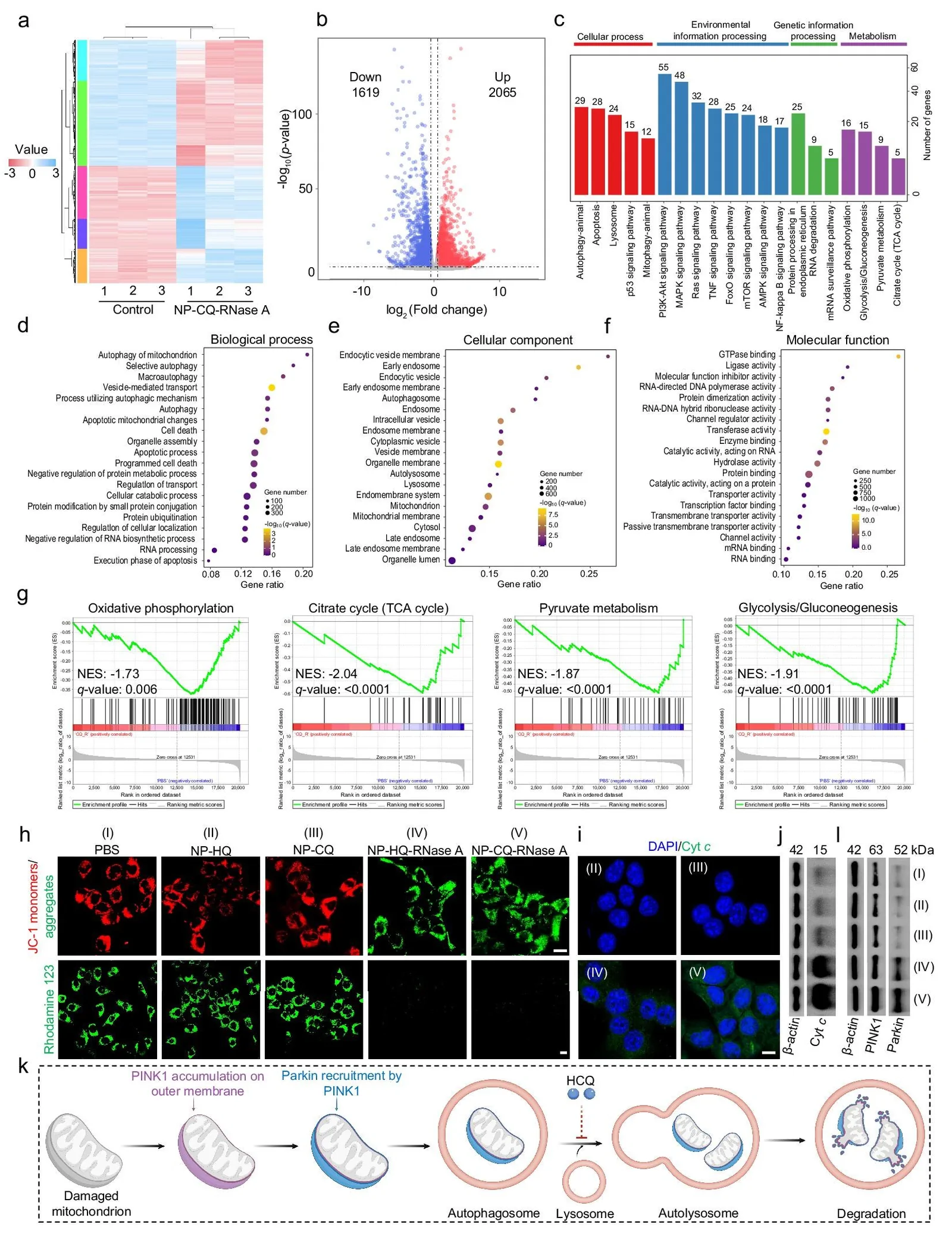
Transcriptomic profiling and mitophagy assessment in 4T1 cells treated with NP-CQ-RNase A. (a) Clustering and heatmap visualization of DEGs following NP-CQ-RNase A treatment. (b) Volcano plot illustrating the distribution of DEGs in 4T1 cells exposed to NP-CQ-RNase A. (c) KEGG pathway enrichment analysis of DEGs identified from NP-CQ-RNase A-treated cells. (d–f) GO enrichment analysis of DEGs across (d) biological processes, (e) cellular components, and (f) molecular functions. (g) GSEA for the depicted signaling pathway. (h) Assessment of ΔΨ_m_ using JC-1 and Rhodamine 123 staining after 24 h treatments (10 μg/mL RNase A). Scale bars = 10 μm. (i) Representative immunofluorescence micrographs showing Cyt *c* localization in 4T1 cells subjected to indicated formulations (10 μg/mL RNase A) for 24 h. Scale bar = 10 μm. (j) Western blot analyses of Cyt *c* levels in 4T1 cells exposed to indicated formulations (10 μg/mL RNase A) for 24 h. (k) Schematic representation of the mitophagy process. (l) Western blot evaluation of PINK1 and Parkin expression levels following 24 h treatments with indicated formulations (10 μg/mL RNase A).

Kyoto Encyclopedia of Genes and Genomes (KEGG) enrichment analyses showed that NP-CQ-RNase A predominantly modulated pathways associated with apoptosis (e.g. apoptosis, p53 signaling, MAPK signaling, TNF signaling), autophagy (e.g. autophagy-animal, mitophagy-animal, PI3K-Akt, Ras, mTOR, and AMPK signaling), and mitochondrial function (e.g. oxidative phosphorylation, citrate cycle, pyruvate metabolism, glycolysis/gluconeogenesis) (Fig. 4c). Gene Ontology (GO) enrichment analysis further revealed that NP-CQ-RNase A significantly influenced biological processes related to apoptosis (e.g. apoptotic mitochondrial changes, apoptotic process, programmed cell death, execution phase of apoptosis) and autophagy (e.g. autophagy of mitochondrion, selective autophagy, macroautophagy, autophagy) (Fig. 4d). Regarding cellular components, the treatment mainly affected autophagosome, autolysosome, mitochondrion, and endolysosomal systems (Fig. 4e). In terms of molecular functions, it notably altered GTPase binding, ligase activity, molecular function inhibitor activity, RNA-directed DNA polymerase activity, protein dimerization activity, and RNA-DNA hybrid ribonuclease activity, among others (Fig. 4f). Gene set enrichment analysis (GSEA) further indicated that NP-CQ-RNase A downregulated mitochondrial-related pathways such as oxidative phosphorylation, citrate cycle, pyruvate metabolism, and glycolysis/gluconeogenesis, suggesting possible mitochondrial impairment (Fig. 4g). Additionally, GSEA confirmed significant enrichment of DEGs in autophagy- and apoptosis-related KEGG pathways and GO terms (Fig. S39).

Given the RNA-seq findings that implicated mitochondrial damage, apoptosis, and mitophagy, we propose that NP-CQ-RNase A induces mitochondrial impairment through RNase A-mediated nonspecific degradation of mitochondria-associated RNA, leading to cytochrome *c* (Cyt *c*) release and subsequent activation of caspase-3-dependent apoptosis [48,49]. Concurrently, mitochondrial damage may activate mitophagy, which can clear damaged mitochondria and unreleased Cyt *c*, thereby attenuating RNase A-induced apoptosis. However, the released HCQ may counteract this protective mitophagy, enhancing apoptosis through increasing intracellular Cyt *c* levels. To test this hypothesis, mitochondrial membrane potential (ΔΨ_m_) was assessed using JC-1 and Rhodamine 123 probes. A loss in ΔΨ_m_ causes JC-1 to shift from red fluorescent aggregates within mitochondria to green fluorescent monomers in the cytosol, while Rhodamine 123 fluorescence intensity decreases. As shown in Fig. 4h, cells exposed to NP-HQ-RNase A and NP-CQ-RNase A exhibited enhanced green JC-1 monomer fluorescence and reduced red JC-1 aggregate fluorescence. Additional confirmatory results and quantitative analysis are provided in Figs S40 and S41. Consistently, these two groups displayed diminished Rhodamine 123 fluorescence (Fig. 4h), indicating severe mitochondrial damage induced by NP-HQ-RNase A and NP-CQ-RNase A. Additional supporting data are shown in Fig. S42. We next evaluated cytosolic Cyt *c* levels via immunofluorescence (Fig. 4i) and western blot (Fig. 4j). Both NP-HQ-RNase A and NP-CQ-RNase A treatments led to significant Cyt *c* upregulation compared to their respective controls without RNase A (Fig. S43), suggesting RNase A damages mitochondria and promotes Cyt *c* release. Notably, NP-CQ-RNase A induced higher Cyt *c* levels than NP-HQ-RNase A, likely due to HCQ-mediated mitophagy inhibition and reduced Cyt *c* degradation. Consistent with the known mechanism where Cyt *c* activates caspase-3 [48,49], the higher cytosolic Cyt *c* level likely accounts for the elevated C-caspase-3 levels detected in NP-CQ-RNase A group in comparison to NP-HQ-RNase A group (Fig. 3j and k). We further investigated mitophagy by analyzing PINK1 and Parkin, key regulators that accumulate on impaired mitochondria and are degraded via lysosomal activity during mitophagy (Fig. 4k). Western blot results showed elevated PINK1 and Parkin expression in both NP-HQ-RNase A and NP-CQ-RNase A groups relative to NP-HQ and NP-CQ groups respectively (Fig. 4l), confirming RNase A-induced mitophagy initiation. Moreover, the NP-CQ-RNase A group showed higher PINK1 and Parkin levels than the NP-HQ-RNase A group, which can be attributed to the inhibition of mitophagy flux by the released HCQ and the resultant reduction in PINK1 and Parkin degradation by lysosomes. In summary, these findings indicate that NP-CQ-RNase A can also enhance RNase A-induced apoptosis by suppressing mitophagy, leading to accumulation of Cyt *c* and activation of caspase-3.

### *In vivo* antitumor efficacy and mechanisms of NP-CQ-RNase A

Encouraged by its outstanding *in vitro* antitumor effects, we further explored the *in vivo* antitumor activity of NP-CQ-RNase A. Prior to efficacy evaluation, the pharmacokinetic behavior of the formulation was examined. As depicted in Fig. S44, NP-CQ-RNase A led to a substantially higher plasma concentration of RNase A in comparison to the free RNase A group. The area under the curve for RNase A in the NP-CQ-RNase A group reached 175.5 µg h mL^−1^, which was about 44 times higher than that of free RNase A (4.0 µg h mL^−1^). In addition, the circulation half-life of NP-CQ-RNase A was 52.6 h, about 105 times longer than that of free RNase A (0.5 h). We next investigated the biodistribution of NP-CQ-RNase A following intravenous administration in 4T1 tumor-bearing mice. Tumors from the NP-CQ-RNase A group exhibited stronger fluorescence signals than those from the free RNase A group at 1, 8, and 24 h post-injection (Figs S45 and S46), suggesting improved intratumoral accumulation and retention of RNase A mediated via the enhanced permeability and retention (EPR) effect. Moreover, Cy5-labelled NP-CQ-RNase A fluorescence was also detected in the liver and kidneys. The signal observed in the liver is likely due to the high presence of Kupffer cells, which efficiently remove nanoparticles through endocytic and phagocytic processes [50]. Meanwhile, accumulation in the kidneys may result from their physiological function in clearing nanoparticles and fluorescent molecules via glomerular filtration and tubular secretion [51].

Subsequently, the *in vivo* antitumor efficiency of NP-CQ-RNase A was assessed in 4T1 tumor-bearing mice via intravenous injection every two days (Fig. 5a). As illustrated in Fig. 5b–f, both NP-HQ-RNase A and NP-CQ-RNase A significantly suppressed tumor growth relative to the PBS, NP-HQ, and NP-CQ control groups. Notably, NP-CQ-RNase A exhibited superior antitumor activity compared to NP-HQ-RNase A, aligning with earlier *in vitro* observations (Fig. 3f–k). Terminal deoxynucleotidyl transferase-mediated dUTP nick-end labeling (TUNEL) staining showed the highest extent of apoptotic cells in the NP-CQ-RNase A-treated tumors (Fig. 5g). Hematoxylin and eosin (H&E) staining further indicated the most pronounced tumor regression in this group (Fig. 5g). In addition, immunohistochemical analysis of Ki67 demonstrated a marked reduction in proliferating cancer cells following NP-CQ-RNase A treatment in comparison to other groups (Fig. 5g).

**Figure 5.**
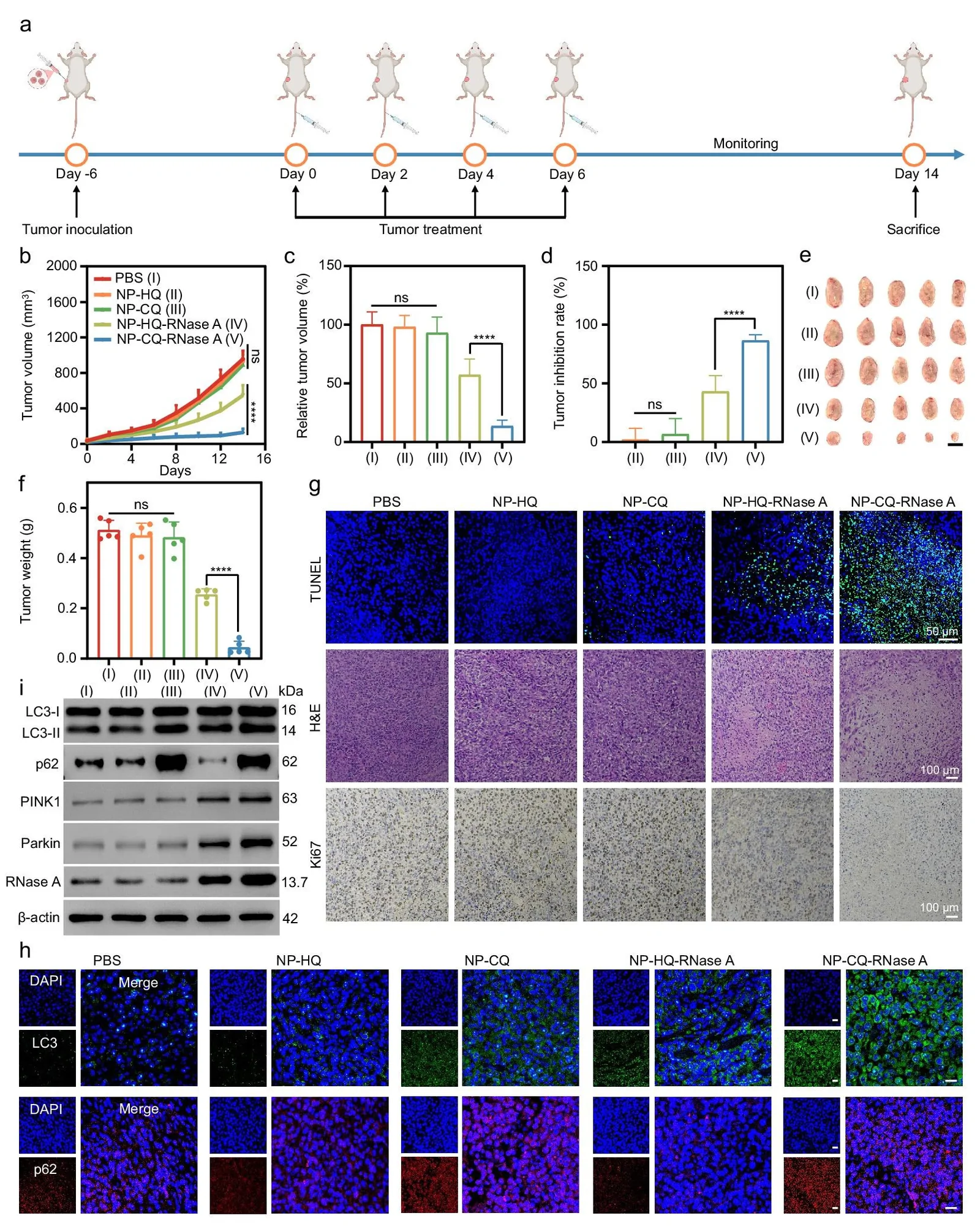
Antitumor efficacy of NP-CQ-RNase A in mice bearing 4T1 tumors. (a) Schematic of the tumor model and therapeutic schedule. (b) Tumor progression profiles in mice receiving different treatments (10.2 mg kg^−1^ RNase A) at specified time points (days 0, 2, 4, and 6). (c) Comparison of final relative tumor volumes across treatment groups on day 14. (d) Calculated tumor growth inhibition rates for indicated groups at the endpoint (day 14). (e) Representative photographic images of excised tumors from all experimental cohorts. Scale bar = 1 cm. (f) Average tumor weights measured in each group following the 14-day treatment period. (g) Analysis of tumor sections via TUNEL, H&E, and Ki67 staining. Scale bars = 50 or 100 μm. (h) Immunofluorescence staining of autophagy markers LC3 and p62 in tumor tissues. Scale bars = 20 μm. (i) Western blot analysis of LC3-I, LC3-II, p62, PINK1, Parkin, RNase A, and β-actin levels in tumors from different groups. ****P < 0.0001, ns: not significant. All quantitative data are shown as mean ± SD (*n* = 5).

We also assessed the autophagy-inhibiting capability of NP-CQ-RNase A *in vivo* through immunofluorescence staining and western blot analyses of LC3 and p62 in tumor tissues. The NP-CQ-RNase A group displayed a greater number of autophagosome-associated green fluorescent dots and more intense p62 fluorescence compared to the NP-HQ-RNase A group (Fig. 5h). Consistent with this, higher protein levels of LC3-II and p62 were detected in NP-CQ-RNase A group (Fig. 5i), supporting robust autophagy inhibition *in vivo*. Furthermore, western blot analysis of tumor tissues showed elevated levels of PINK1 and Parkin in the NP-CQ-RNase A group relative to the NP-HQ-RNase A group (Fig. 5i), confirming effective mitophagy suppression. RNase A levels in tumor tissues were also evaluated by western blot. As shown in Fig. 5i, treatment with NP-HQ-RNase A and NP-CQ-RNase A led to higher intratumoral RNase A levels compared to other groups, confirming successful *in vivo* delivery. Notably, the NP-CQ-RNase A group exhibited significantly higher RNase A levels than the NP-HQ-RNase A group, suggesting that this enhancement resulted from HCQ-mediated inhibition of autophagic degradation. These results demonstrate that HCQ released from NP-CQ-RNase A effectively blocks autophagic RNase A degradation *in vivo*, which together with its suppression of mitophagy contributes to the enhanced antitumor efficacy.

The biosafety of NP-CQ-RNase A was subsequently evaluated by monitoring body weight changes in treated mice. No obvious differences in average body weight were found between the NP-CQ-RNase A and PBS control groups (Fig. S47), indicating a favorable safety profile. H&E staining of major organs further showed no notable pathological alterations in the NP-CQ-RNase A group compared to PBS-treated mice (Fig. S48), reinforcing its high biocompatibility. Additionally, serum biochemical analysis showed comparable levels of alanine aminotransferase (ALT), aspartate aminotransferase (AST), alkaline phosphatase (ALP), blood urea nitrogen (BUN), creatinine (CREA), and uric acid (UA) between the NP-CQ-RNase A and PBS groups (Fig. S49), suggesting no adverse effects on hepatic or renal function. Routine blood examinations, such as white blood cell (WBC), red blood cell (RBC), mean corpuscular volume (MCV), hematocrit (HCT), hemoglobin (HGB), and mean corpuscular hemoglobin concentration (MCHC), also revealed no significant variations between the two groups (Fig. S50). In summary, these findings demonstrate that NP-CQ-RNase A effectively delivers functional RNase A into tumor tissues, significantly inhibiting tumor growth, while exhibiting excellent biocompatibility and minimal systemic toxicity.

### Generality of NP-CQ for cytosolic protein delivery

To assess the generality of NP-CQ for intracellular protein delivery, we employed model proteins with varying molecular weights and isoelectric points (pI), including GFP (31.5 kDa, pI 6.6), β-Gal (465 kDa, pI 4.6), and HRP (40.0 kDa, pI 7.2), in addition to RNase A (13.7 kDa, pI 9.6). The corresponding protein-loaded nanoparticles (NP-HQ-GFP, NP-CQ-GFP, NP-HQ-Gal, NP-CQ-Gal, NP-HQ-HRP, and NP-CQ-HRP) were successfully fabricated, exhibiting a DLC of 18.6%–27% and a DLE of 94.4%–97.9% for the encapsulated proteins (Table S1). These nanoparticles exhibited sizes in the range of 150–200 nm (Fig. S51), a narrow size distribution, and a negatively charged surface (Fig. S52). The GFP used in this study was engineered to incorporate a nuclear localization signal (NLS), enabling its translocation from the cytosol into the nucleus (Fig. S53A) [52,53]. Owing to this feature, the intracellular distribution of GFP could be visualized to evaluate endolysosomal escape efficiency. As depicted in Fig. S53B, cells treated with NP-CQ-GFP displayed pronounced green fluorescence within the nuclei, indicating that the protein was successfully released from the nanoparticles in acidic endolysosomes, entered the cytosol following HCQ-mediated endolysosomal disruption, and subsequently underwent active nuclear import guided by NLS. Although the NP-HQ-GFP group showed a fluorescence distribution similar to that of the NP-CQ-GFP group, confirming effective endolysosomal escape, its fluorescence intensity was significantly weaker. This reduction is likely due to the absence of HCQ and the consequent inability to inhibit autophagic degradation of GFP. Furthermore, GFP alone showed no discernible cellular fluorescence, comparable to the PBS control, confirming its poor membrane permeability. Flow cytometric analysis further corroborated these findings, revealing significantly higher mean fluorescence intensities in cells incubated with NP-CQ-GFP relative to those treated with NP-HQ-GFP (Fig. S53C).

We next evaluated the delivery of β-Gal, an enzyme that can catalyze the hydrolysis of X-Gal to yield an insoluble blue precipitate, thereby serving as a visual indicator of intracellular bioactive β-Gal (Fig. S53D) [54]. Following X-Gal staining, cells exposed to NP-CQ-Gal showed more intense blue staining than those receiving NP-HQ-Gal (Fig. S53E). Consistently, a β-Gal activity assay demonstrated approximately 2-fold higher enzymatic activity in the NP-CQ-Gal group in comparison to the NP-HQ-Gal group (Fig. S53F). These results indicate that NP-CQ reduces autophagic degradation and facilitates cytosolic retention of bioactive β-Gal. Free β-Gal, as expected, showed neither visible staining nor significant enzymatic activity, attributable to its inability to cross the plasma membrane. We further investigated HRP delivery using a colorimetric assay in which HRP catalyzes the H_2_O_2_-dependent oxidation of TMB, generating a soluble blue product (Fig. S53G) [54]. In the presence of TMB and H_2_O_2_, cells treated with NP-CQ-HRP produced substantially more blue product than any other group (Fig. S53H). Quantitative analysis indicated that the HRP activity in the NP-CQ-HRP group was higher than that in the NP-HQ-HRP group (Fig. S53I). These findings confirm that NP-CQ facilitates more effective accumulation of functionally intact HRP in the cytosol. Beyond 4T1 cells, we extended our evaluation of NP-CQ-mediated protein delivery to multiple cell lines, including human lung adenocarcinoma (A549), mouse osteosarcoma (K7), mouse melanoma (B16F10), human umbilical vein endothelial cell (HUVEC), human embryonic kidney (293T), and mouse fibroblast (L929) cells. In all cases, NP-CQ efficiently delivered GFP into the nuclei (Fig. S53J). This cell line-independent protein delivery capability was further confirmed by additional data (Fig. S54). Moreover, NP-CQ delivered RNase A more efficiently than a commercial lipid reagent, Lipofectamine 2000 (Fig. S55). In summary, these results establish the versatility of the NP-CQ platform, demonstrating its dual functionality in cytosolic delivery and efficacy enhancement of diverse proteins.

## CONCLUSION

In summary, we report a stepwise cooperative nanoplatform that enables traceless cytosolic protein delivery and concurrent autophagy inhibition for enhanced tumor therapy. The system was constructed using a tailor-made amphiphilic triblock copolymer consisting of mPEG, a 2-APBA-functionalized segment, and an HCQ-conjugated block, which efficiently loaded RNase A through pH-reversible iminoboronates. The resulting nanoparticles exhibited favorable stability, extended blood circulation, and improved intratumoral accumulation. Upon cell uptake, RNase A was tracelessly released in acidic endolysosomes through acid-labile cleavage of iminoboronates and subsequently escaped into the cytoplasm through HCQ-mediated ‘proton sponge’ effects. The subsequent GSH-triggered release of HCQ in the reducing cytosolic environment suppressed both RNase A degradation and mitophagy, thereby potentiating RNase A-induced apoptosis, as validated both *in vitro* and *in vivo*. Moreover, this nanoplatform could also deliver other functional proteins including GFP, β-Gal, and HRP into the cytosol while enhancing their bioactivity. Collectively, this work presents a versatile and robust strategy to enhance protein-based therapeutics through traceless delivery and autophagy disruption. However, despite these advantages, the clinical translation of this nanoplatform remains challenged by the lack of active tumor-targeting capability, the need for scalable and reproducible manufacturing, and associated regulatory complexities. These challenges may be addressed by incorporating tumor-selective ligands or engineering stimuli-responsive release mechanisms to enhance targeting specificity, alongside process optimization and standardized automated production to ensure scalability and consistency. In parallel, early integration of regulatory considerations, including rigorous characterization, comprehensive safety evaluation, and compliance with Good Manufacturing Practice (GMP), will be critical to facilitate clinical translation. Despite these hurdles, this nanoplatform holds significant promise for therapeutic applications through coordinated multidisciplinary efforts spanning biology, chemistry, engineering, and clinical medicine.

## MATERIALS AND METHODS

Detailed materials and methods can be found in the online supplementary file.

### Ethical statements

All animal procedures were approved by the Ethics Committee of the Changchun Institute of Applied Chemistry, Chinese Academy of Sciences. The approval number for animal experiments is 202124.

## Supplementary Material

nwag446_Supplemental_File
