# Supplementary material for: Stepwise-coordinated polymeric nanoplatform enables traceless cytosolic protein delivery and autophagy blockade for enhanced tumor therapy
Source: Natl Sci Rev. 2026 Jul 20;13(18):nwag446. doi: 10.1093/nsr/nwag446 (PMC13622349; doi:10.1093/nsr/nwag446)
Supplement: Supplementary file 1 [file nwag446_supplemental_file.pdf]

# Supplementary Information

**Stepwise-Coordinated Polymeric Nanoplatform Enables Traceless Cytosolic Protein Delivery and Autophagy Blockade for Enhanced Tumor Therapy**

Peng Zhang,[1] Yaowei Zhu,[2] Hongyu Zhang,[1] Shuai Tang,[3] Zhihui Guo,[1] Xuan Yi,[1,]* Chunsheng Xiao, [1,]* and Xuesi Chen[1]

[1] State Key Laboratory of Polymer Science and Technology, Changchun Institute of Applied Chemistry, Chinese Academy of Sciences, Changchun 130022, P. R. China

[2] College of Chemistry and Chemical Engineering, Taishan University, Tai'an 271000, P. R. China

[3] State Key Laboratory of Supramolecular Structure and Materials, College of Chemistry, Jilin University, Changchun 130012, P. R. China

* Corresponding authors: yixuan@ciac.ac.cn (X. Yi); xiaocs@ciac.ac.cn (C. Xiao)

## Experimental Section

### Cells and Animals

4T1, A549, K7, B16F10, L929, 293T, and HUVEC cells were bought from Chinese Academy of Sciences cell bank and cultured in RPMI-1640 or DMEM supplemented with 10% FBS and antibiotics under 5% $CO_2$ at 37 °C. Female BALB/c mice (6–8 weeks) were obtained from Beijing Vital River Laboratory Animal Technology Co. Ltd. (China). All animal procedures were approved by the Ethics Committee of the Changchun Institute of Applied Chemistry, Chinese Academy of Sciences.

### Synthesis of mPEG-*b*-PBLG-*b*-PPLG

Methoxy poly (ethylene glycol)-*block*-poly(γ-benzyl-L-glutamate)-*block*-poly(γ-propargyl-L-glutamate) (mPEG-*b*-PBLG-*b*-PPLG) was synthesized by sequential ring-opening polymerization (ROP) of BLG-NCA and PLG-NCA using amino-terminated mPEG ($mPEG-NH_2$) as a macroinitiator. In brief, anhydrous $mPEG-NH_2$ (2 g) was dissolved in dry DMF (35 mL) in a flame-dried flask. Then BLG-NCA (2.65 g) was added into the flask quickly. After stirring for 72 h at room temperature (RT), PLG-NCA (1.7 g) in anhydrous DMF (15 mL) was added to the flask and stirring for another 72 h at RT. Finally, DMAP (58.65 mg) and acetic anhydride (2 mL) were added to the flask and stirring for another 24 h to block the terminal amine groups. mPEG-*b*-PBLG-*b*-PPLG was precipitated into excessive diethyl ether and washed with diethyl ether thrice. The resultant product was dried under vacuum to obtain a white powder (yield: 77%).

### Synthesis of mPEG-*b*-PLGA-*b*-PPLG

mPEG-*b*-PBLG-*b*-PPLG (2.5 g) was dissolved in dichloroacetic acid (25 mL) at RT in a flask. Then, 33% HBr in acetic acid solution (7.5 mL) was added into the flask to remove γ-benzyl group. The reaction was proceeded for 1.5 h at 30 °C. Subsequently, the product was precipitated into excessive diethyl ether and was redissolved in DMF. After dialyzed against distilled (DI) water (MWCO = 3.5 kDa) for 2 d, mPEG-*b*-PLGA-*b*-PPLG was obtained as a white powder after lyophilization (yield: 50%).

### Synthesis of HO-SS-Br

Triphenylphosphine (1.3 g, 5.0 mmol) was added to an ice-cold solution of 2-hydroxyethyl disulfide (0.77 g, 5.0 mmol) in DCM (300 mL). After the mixture was stirred at 0 °C for 15 min, carbon tetrabromide (1.66 g, 5.0 mmol) was added slowly. The mixture was stirred for a further 10 h and then washed with

water (2 × 150 mL) and brine (150 mL). The organic layer was dried ($Na_2SO_4$), concentrated, and purified by silica gel column chromatography (20% ethyl acetate in petroleum ether) to afford HO-SS-Br as a colorless oil (yield: 58%).

**Synthesis of HO-SS-$N_3$**

Sodium azide (0.65 g, 10 mmol) was added to a solution of HO-SS-Br (1.08 g, 5 mmol) in dry DMF (40 mL), and the mixture was stirred at 80 °C for 10 h. The product was extracted three times with ethyl ether. The combined organic layer was washed with brine and dried over anhydrous $Na_2SO_4$. Following filtration and evaporation, the product was dried under a vacuum to give HO-SS-$N_3$ as a yellow oil (yield: 80%).

**Synthesis of HCQ-IMI**

CDI (0.2 g, 1.2 mmol) was dissolved in dry DCM (20 mL). Then, HCQ (0.2 g, 0.6 mmol) was dissolved in dry DCM (10 mL) and added dropwise to the solution of CDI. The reaction was stirred for 24 h at RT. Then, the reaction mixture was washed with water (3 × 20 mL). Afterwards, the organic layer was collected and dried over anhydrous $Na_2SO_4$. Following filtration and evaporation, the product was dried under a vacuum to give HCQ-IMI (yield: 69%).

**Synthesis of HCQ-SS-$N_3$**

HCQ-IMI (175 mg, 0.41 mmol) was dissolved in dry DCM (10 mL). Then, HO-SS-$N_3$ (73 mg, 0.41 mmol) was dissolved in dry DCM (10 mL) and added dropwise to the solution of HCQ-IMI. The reaction was stirred for 24 h at RT. Then, the reaction mixture was washed with water (3 × 20 mL). Afterwards, the organic layer was collected and dried over anhydrous $Na_2SO_4$. Following filtration and evaporation, the product was dried under a vacuum to give HCQ-SS-$N_3$ (yield: 61%).

**Synthesis of HQ**

HCQ (1 g, 3.0 mmol) was dissolved in ethanol (20 mL). Pd/C (100 mg) was added into the solution and the mixture was stirred for 24 h under $H_2$. Pd/C was removed by filtration and the liquid was collected and dried to obtain HQ (yield: 50%).

**Synthesis of HQ-IMI**

CDI (0.2 g, 1.2 mmol) was dissolved in dry DCM (20 mL). Then, HQ (0.6 mmol) was dissolved in dry DCM (10 mL) and added dropwise to the solution of CDI. The reaction was stirred for 24 h at RT. Then, the reaction mixture was washed with water (3 × 20 mL). Afterwards, the organic layer was collected and

dried over anhydrous $Na_2SO_4$. Following filtration and evaporation, the product was dried under a vacuum to give HQ-IMI (yield: 68%).

**Synthesis of HQ-SS-$N_3$**

HQ-IMI (0.41 mmol) was dissolved in dry DCM (10 mL). Then, HO-SS-$N_3$ (73 mg, 0.41 mmol) was dissolved in dry DCM (10 mL) and added dropwise to the solution of HQ-IMI. The reaction was stirred for 24 h at RT. Then, the reaction mixture was washed with water (3 × 20 mL). Afterwards, the organic layer was collected and dried over anhydrous $Na_2SO_4$. Following filtration and evaporation, the product was dried under a vacuum to give HQ-SS-$N_3$ (yield: 63%).

**Synthesis of intermediate 1 of 2-APBA-$NH_2$**

Tert-butyl (3-bromopropyl) carbamate (3.00 g, 12.6 mmol) and 2,4-dihydroxyacetophenone (2.13 g, 14.0 mmol) were dissolved in 30 mL of acetone. $K_2CO_3$ (7.74 g, 56.0 mmol) was added and the reaction was allowed to reflux at 65 °C overnight. Acetone was then evaporated and the residue was dissolved in 100 mL water. The product was extracted with EtOAC (3 × 100 mL). The organic layers were combined and washed with saturated brine (150 mL) and dried over anhydrous $Na_2SO_4$. Following filtration and evaporation, the product was dried under a vacuum to give **1** (yield: 73%).

**Synthesis of intermediate 2 of 2-APBA-$NH_2$**

Intermediate **1** (2.00 g) was dissolved in 40 mL dry DCM. Triethylamine (2.17 g, 21.5 mmol) was added to the solution and the mixture was kept at -78 °C for 5 min. Trifluoromethane sulfonic anhydride (3.52 g, 12.4 mmol) was added slowly during 5 min. The reaction was warmed up to RT and allowed to stir for 1 h. The reaction was subsequently quenched with 40 mL saturated sodium bicarbonate. The mixture was stirred for 5 min and the product was extracted with DCM (3 × 100 mL). The combined organic layer was washed with brine (100 mL) and dried over anhydrous $Na_2SO_4$. The solvent was removed and the product was purified via silica gel column chromatography using EtOAc/Hexane (1:4) to give the desired product as a light orange solid (yield: 70%).

**Synthesis of intermediate 3 of 2-APBA-$NH_2$**

Intermediate **2** (1.00 g, 2.27 mmol), [1,1′-bis(diphenylphosphino)ferrocene]dichloropalladium(II) (0.20 g, 0.27 mmol), potassium acetate (0.8 g, 8.16 mmol), and bis(pinacolato)diboron (1.40 g, 5.51 mmol) were dissolved in 20 mL of anhydrous dioxane, to which ~100 mg of 3 Å molecular sieves were added. The

reaction was bubbled with argon for 15 min and allowed to stir for 1 h at 85 °C. The reaction was cooled down and 50 mL of water was added to the reaction. The product was extracted with EtOAc (3 × 100 mL). The combined organic layer was washed with brine (100 mL) and dried over anhydrous $Na_2SO_4$. The solvent was removed and the product was purified via silica gel column chromatography using EtOAc/Hexane (3:7) to give **3** as a light yellow viscous liquid (yield: 80%).

**Synthesis of 2-APBA-NH$_2$**

Intermediate **3** (0.91 g) was dissolved in 10 mL of dioxane containing 4 M HCl. The reaction was stirred at RT for 2 h. Subsequently, the product was precipitated into excessive diethyl ether and redissolved in methanol. The product in methanol was then precipitated into excessive diethyl ether again. The product was dried under a vacuum to give 2-APBA-NH$_2$ (yield: 69%).

**Synthesis of mPEG-*b*-PLGA-*b*-PGCQ**

mPEG-*b*-PLGA-*b*-PPLG (1.16 g, 2 mmol alkyne pendants) and HCQ-SS-N$_3$ (2.16 g, 4 mmol) was dissolved in anhydrous DMSO (35 mL) and flushed with nitrogen for 20 min. Then sodium ascorbate (0.214 g, 1.08 mmol) was added into the solution. After another 5 min with nitrogen flushing, copper sulfate pentahydrate (0.1225 g, 0.49 mmol) was added to the solution. After reaction for 3 d at 40 °C under nitrogen atmosphere, the solution was dialyzed against DI water containing hydrochloric acid for 24 h and against DI water (MWCO = 3.5 kDa) for 72 h, respectively. Then mPEG-*b*-PLGA-*b*-PGCQ was obtained after lyophilization (yield: 63%).

**Synthesis of mPEG-*b*-PGBA-*b*-PGCQ**

mPEG-*b*-PLGA-*b*-PGCQ (0.5 g, 0.557 mmol carboxyl pendants) was dissolved in anhydrous DMSO (40 mL) and flushed with nitrogen for 20 min. 2-APBA-NH$_2$ hydrochloride (0.152 g, 0.557 mmol) was added to the solution and flushed with nitrogen for 5 min. Then NHS (0.077 g, 0.668 mmol) was added to the solution and flushed with nitrogen for another 5 min. Finally, EDC·HCl (0.128 g, 0.668 mmol) was added and the reaction was proceeded in nitrogen atmosphere for 48 h. Subsequently, the solution was dialyzed against DI water containing hydrochloric acid for 24 h and against DI water (MWCO = 3.5 kDa) for 72 h before collection. mPEG-*b*-PGBA-*b*-PGCQ was obtained after lyophilization (yield: 81%).

**Synthesis of mPEG-*b*-PLGA-*b*-PGHQ**

mPEG-*b*-PLGA-*b*-PPLG (1.16 g, 2 mmol alkyne pendants) and HQ-SS-$N_3$ (2.02 g, 4 mmol) was dissolved in anhydrous DMSO (35 mL) and flushed with nitrogen for 20 min. Then sodium ascorbate (0.214 g, 1.08 mmol) was added into the solution. After another 5 min with nitrogen flushing, copper sulfate pentahydrate (0.1225 g, 0.49 mmol) was added to the solution. After reaction for 3 d at 40 °C under nitrogen atmosphere, the solution was dialyzed against DI water containing hydrochloric acid for 24 h and against DI water (MWCO = 3.5 kDa) for 72 h, respectively. Then mPEG-*b*-PLGA-*b*-PGHQ was obtained after lyophilization (yield: 70%).

**Synthesis of mPEG-*b*-PGBA-*b*-PGHQ**

mPEG-*b*-PLGA-*b*-PGHQ (0.484 g, 0.557 mmol carboxyl pendants) was dissolved in anhydrous DMSO (40 mL) and flushed with nitrogen for 20 min. 2-APBA-$NH_2$ hydrochloride (0.152 g, 0.557 mmol) was added to the solution and flushed with nitrogen for 5 min. Then NHS (0.077 g, 0.668 mmol) was added to the solution and flushed with nitrogen for another 5 min. Finally, EDC·HCl (0.128 g, 0.668 mmol) was added and the reaction was proceeded in nitrogen atmosphere for 48 h. Subsequently, the solution was dialyzed against DI water containing hydrochloric acid for 24 h and against DI water (MWCO = 3.5 kDa) for 72 h before collection. mPEG-*b*-PGBA-*b*-PGHQ was obtained after lyophilization (yield: 83%).

**Synthesis of Cy5-modified RNase A (RNase A-Cy5)**

RNase A (6 mg) was dissolved in sodium carbonate-bicarbonate buffer (2 mL, 0.1 M, pH 9.0). Then Cy5-NHS (0.876 μmol) was dissolved in DMSO (5 mg $mL^{-1}$) and added to the RNase A solution. After an incubation at RT overnight, RNase A-Cy5 was purified by dialysis against DI water (MWCO = 3 kDa) and lyophilized. The whole experiment process was protected from light.

**Nanoparticle Preparation**

Protein-loaded nanoparticles were fabricated by a two-step procedure. First, the polymer (e.g., mPEG-*b*-PGBA-*b*-PGCQ) was dissolved in DMSO and dropwise added into DI water. After stirring and dialysis, blank nanoparticles (NP-CQ) were collected by lyophilization. Subsequently, NP-CQ was incubated with the protein (e.g., RNase A) in PBS, and the complex (NP-CQ-RNase A) was purified by ultrafiltration (100 kDa cutoff). Drug loading content (DLC) and efficiency (DLE) were calculated based on the fluorescence of Cy5-labeled protein.

**In Vitro Release Studies**

RNase A release from NP-CQ-RNase A-Cy5 was assessed by dialysis against buffers at pH 7.4, 6.5, or 5.5. HCQ/HQ release was evaluated in PBS (pH 7.4) containing 0, 1, or 10 mM GSH. Released amounts were quantified by fluorescence (RNase A-Cy5) or UV-Vis absorbance (HCQ/HQ at 343 nm).

**Cellular Uptake and Mechanism**

4T1 cells were exposed to NP-HQ-RNase A-Cy5 or NP-CQ-RNase A-Cy5 (10 μg $mL^{-1}$ RNase A) for 1–6 h, then analyzed by flow cytometry. Endocytosis pathways were probed using amiloride, chlorpromazine, nystatin, or $NaN_3$ as inhibitors prior to nanoparticle exposure.

**Intracellular Trafficking and Organelle Effect**s

Confocal microscopy was employed to track lysosomal escape (LysoTracker Green co-staining), lysosomal membrane permeabilization (galectin-3 immunofluorescence), and autophagosome formation (LC3 immunofluorescence). Mitochondrial membrane potential (ΔΨm) was evaluated with JC-1 or Rhodamine 123 staining followed by microscopy or flow cytometry.

**Cytotoxicity and Apoptosis**

Cell viabilities were determined by MTT assays after a 72 h treatment. Live/dead staining used Calcein AM/PI. Apoptosis was quantified by flow cytometry after Annexin V-FITC/PI staining. Immunofluorescence visualized cytochrome c release and cleaved caspase-3 expression.

**RNA-Sequencing**

4T1 cells treated by PBS or NP-CQ-RNase A for 24 h were lysed in TRIzol, and RNA-seq was performed by Scale Biomedicine Technology Co., Ltd. (Beijing, China).

**Pharmacokinetics and Biodistribution**

Mice received intravenous injections of RNase A-Cy5 or NP-CQ-RNase A-Cy5 (3.6 mg $kg^{-1}$ RNase A). Blood was collected at intervals for plasma fluorescence measurement. For biodistribution, 4T1-tumor-bearing mice were injected similarly, and organs/tumors were excised at 1, 8, and 24 h for ex vivo fluorescence imaging.

**In Vivo Antitumor Activity**

Female BALB/c mice were subcutaneously injected with $1\times10^6$ 4T1 cells in the right flank. When the tumors grew to approximately 100 $mm^3$, the mice were divided into five groups (n = 5) and were intravenously injected with PBS, NP-HQ, NP-CQ, NP-HQ-RNase A and NP-CQ-RNase A with an

equivalent dosage of RNase A (10.2 mg/kg) on day 0, 2, 4, and 6. Tumor sizes and body weights of the mice in each group were recorded every two days. The tumor volume was calculated as V ($mm^3$) = length × $(width)^2/2$. Finally, on day 14, the tumors and major organs such as heart, liver, spleen, lung and kidney of mice in each group were collected for hematoxylin and eosin (H&E) staining analysis, terminal deoxynucleotidyl transferase-mediated dUTP nick-end labeling (TUNEL) staining analysis, and Ki-67 staining analysis. The blood of each group was collected for blood analyses.

**Cytosolic Delivery of Other Proteins**

GFP, β-Gal, and HRP were loaded into nanoparticles as described above. Delivery efficiency was evaluated by confocal microscopy (GFP), enzymatic staining (β-Gal with X-Gal; HRP with TMB), and quantitative activity assays (β-Gal and HRP). Flow cytometry was also used for GFP-positive cell quantification.

**Statistical Analysis**

Data are shown as mean ± SD. Significance is determined via one-way ANOVA using GraphPad Prism 8.0 (*$p < 0.05$, **$p < 0.01$, ***$p < 0.001$, ****$p < 0.0001$, ns: not significant).

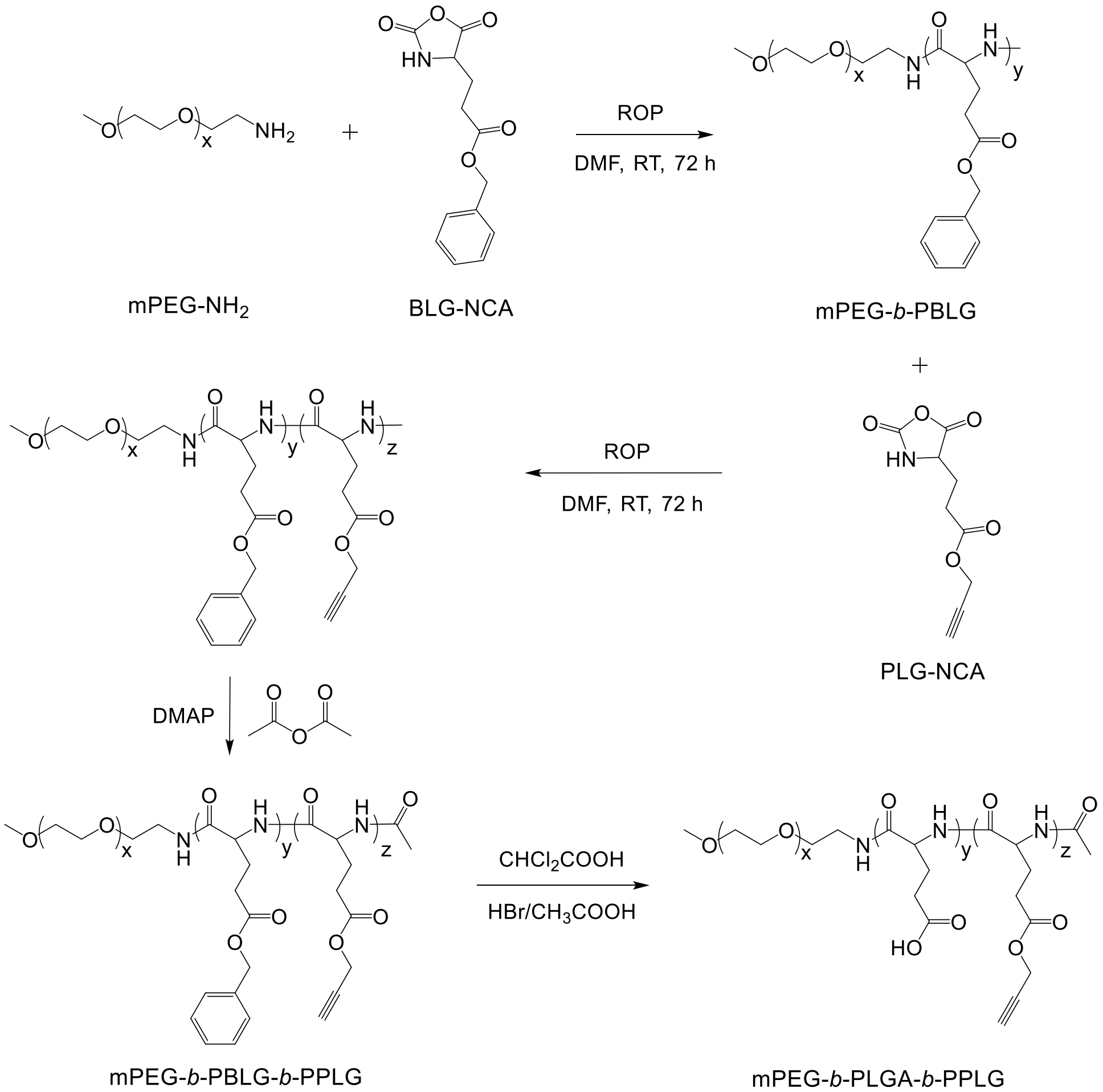


**Figure S1.** Synthetic route of mPEG-*b*-PLGA-*b*-PPLG.

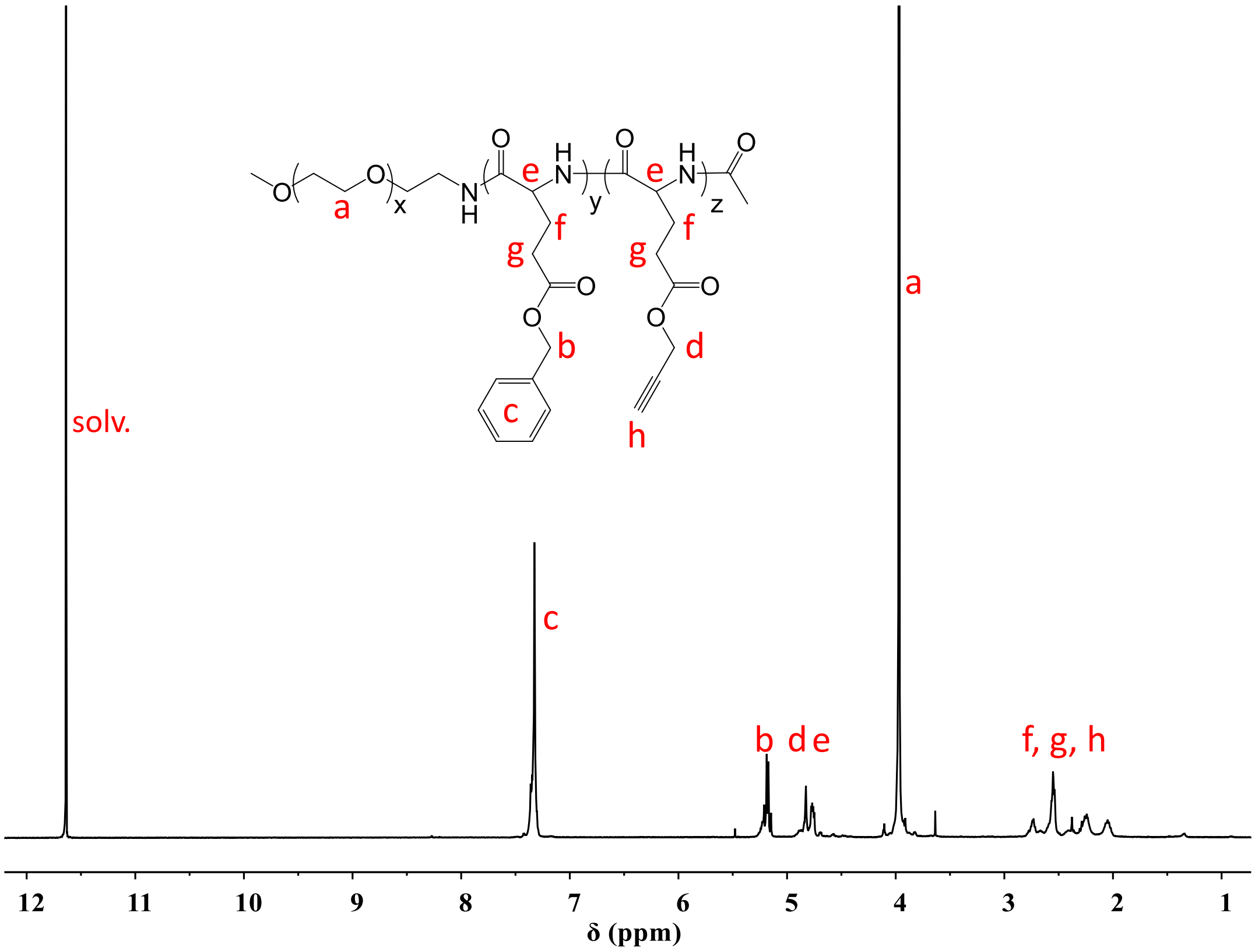


**Figure S2.** $^{1}$H NMR spectrum of mPEG-*b*-PBLG-*b*-PPLG in $CF_3COOD$.

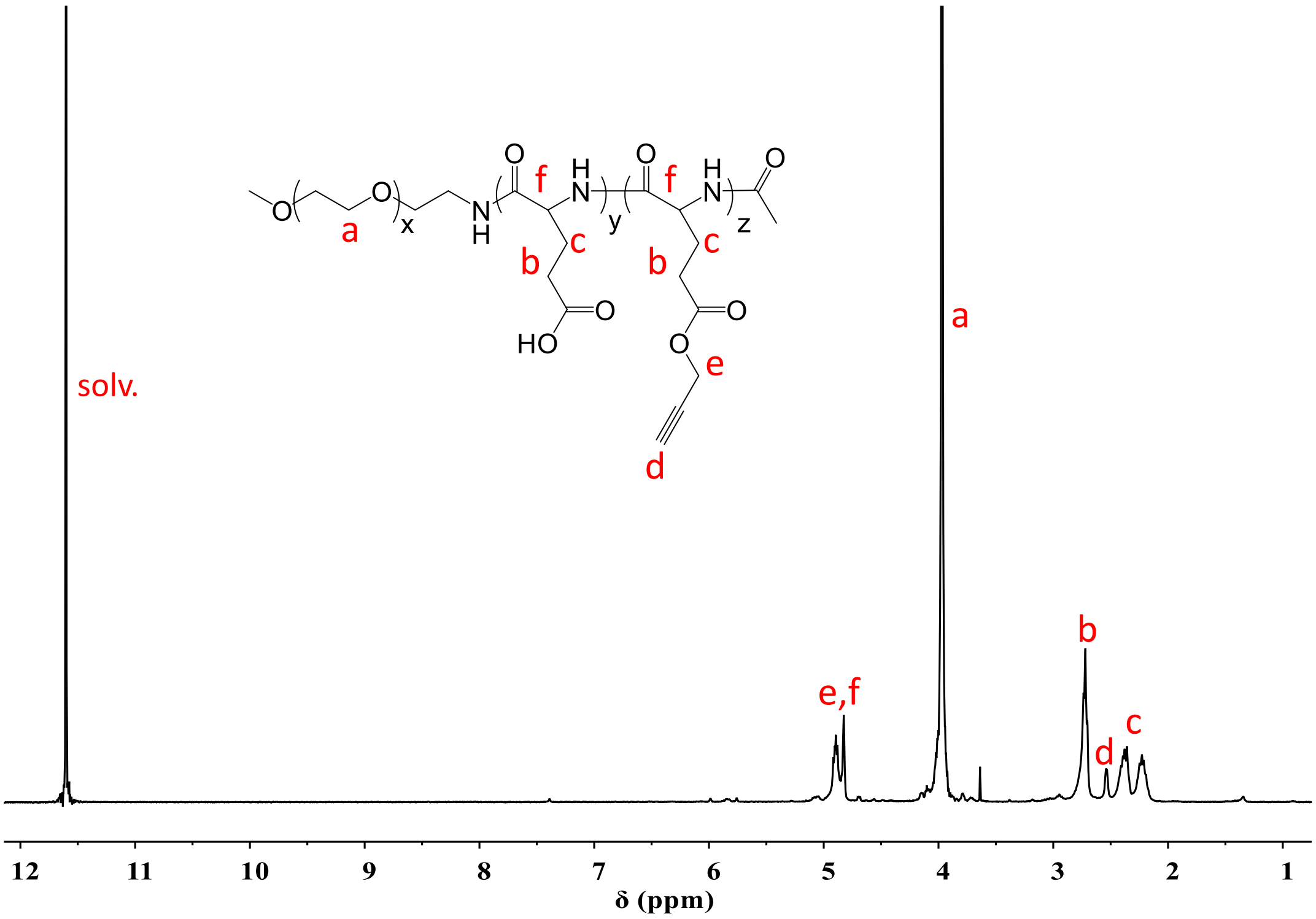


**Figure S3.** $^{1}$H NMR spectrum of mPEG-*b*-PLGA-*b*-PPLG in $CF_3COOD$.

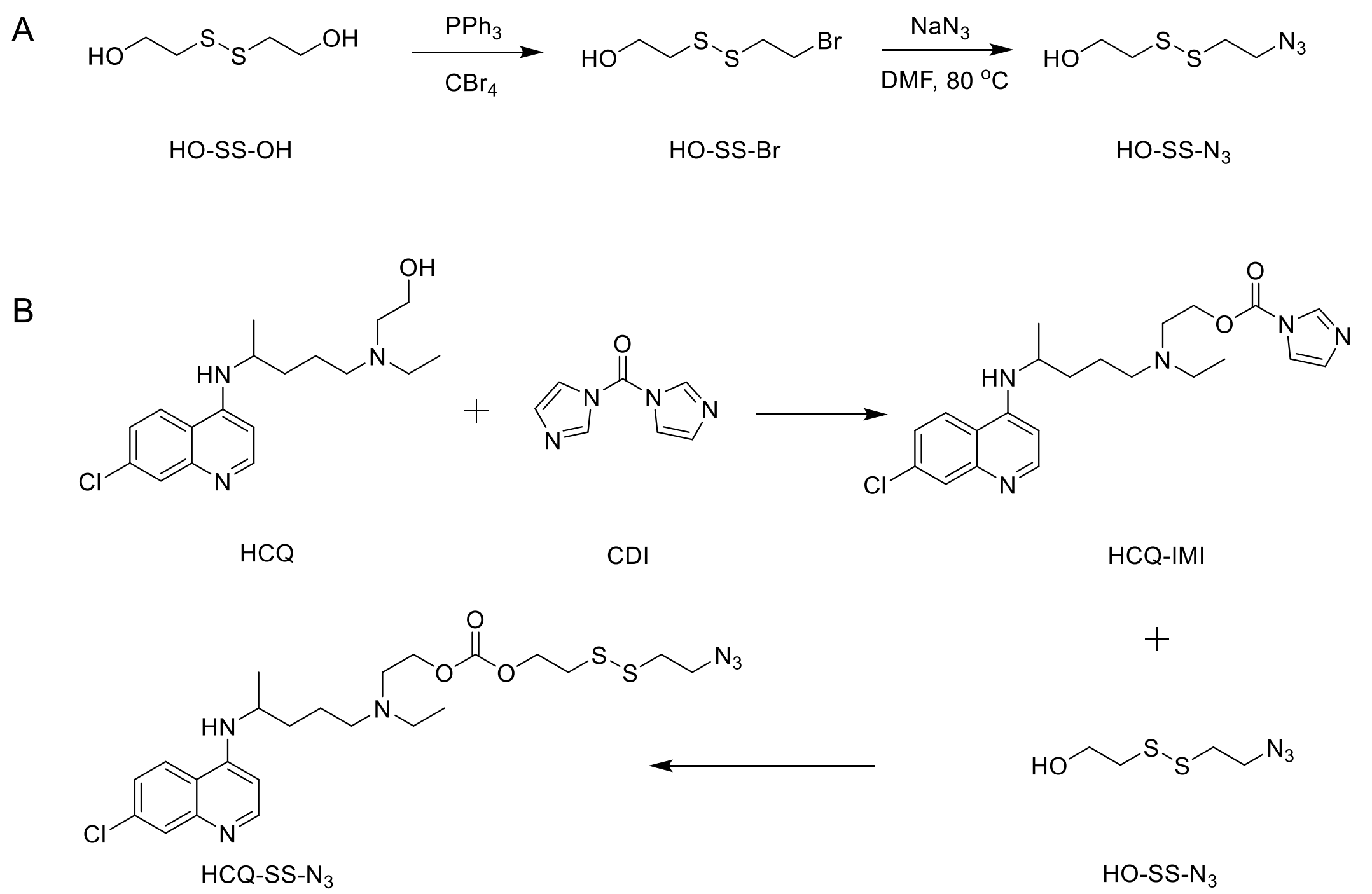


**Figure S4.** (A) Synthetic route of HO-SS-$N_3$. (B) Synthetic route of HCQ-SS-$N_3$.

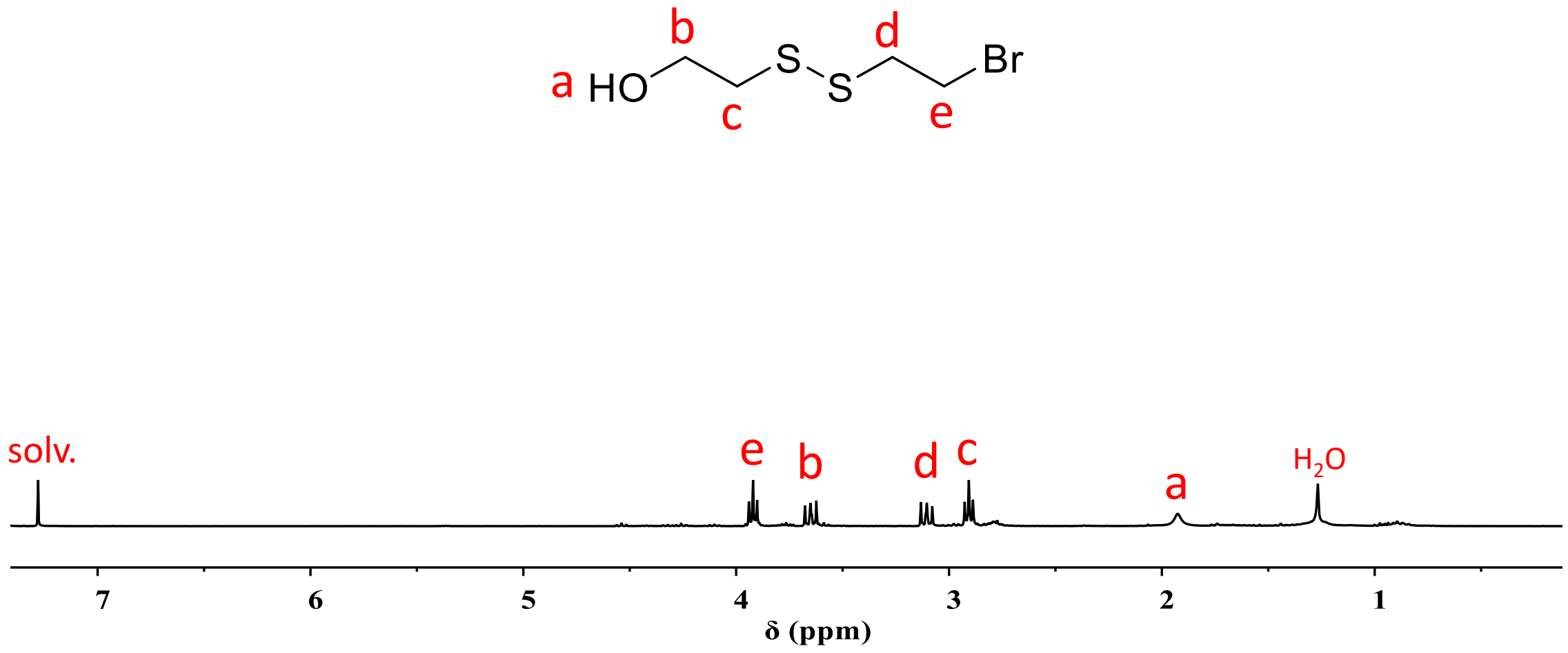


**Figure S5.** $^{1}H$ NMR spectrum of HO-SS-Br in $CDCl_3$.

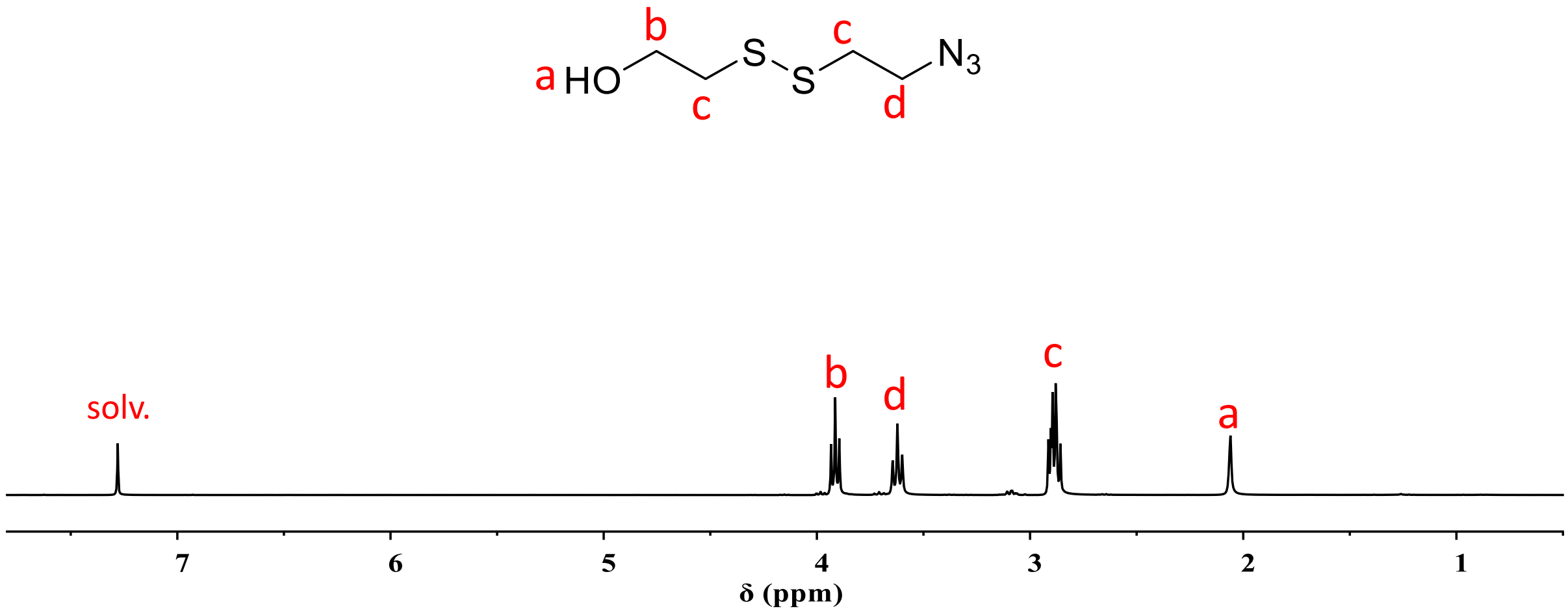


**Figure S6.** $^{1}H$ NMR spectrum of HO-SS-$N_3$ in $CDCl_3$.

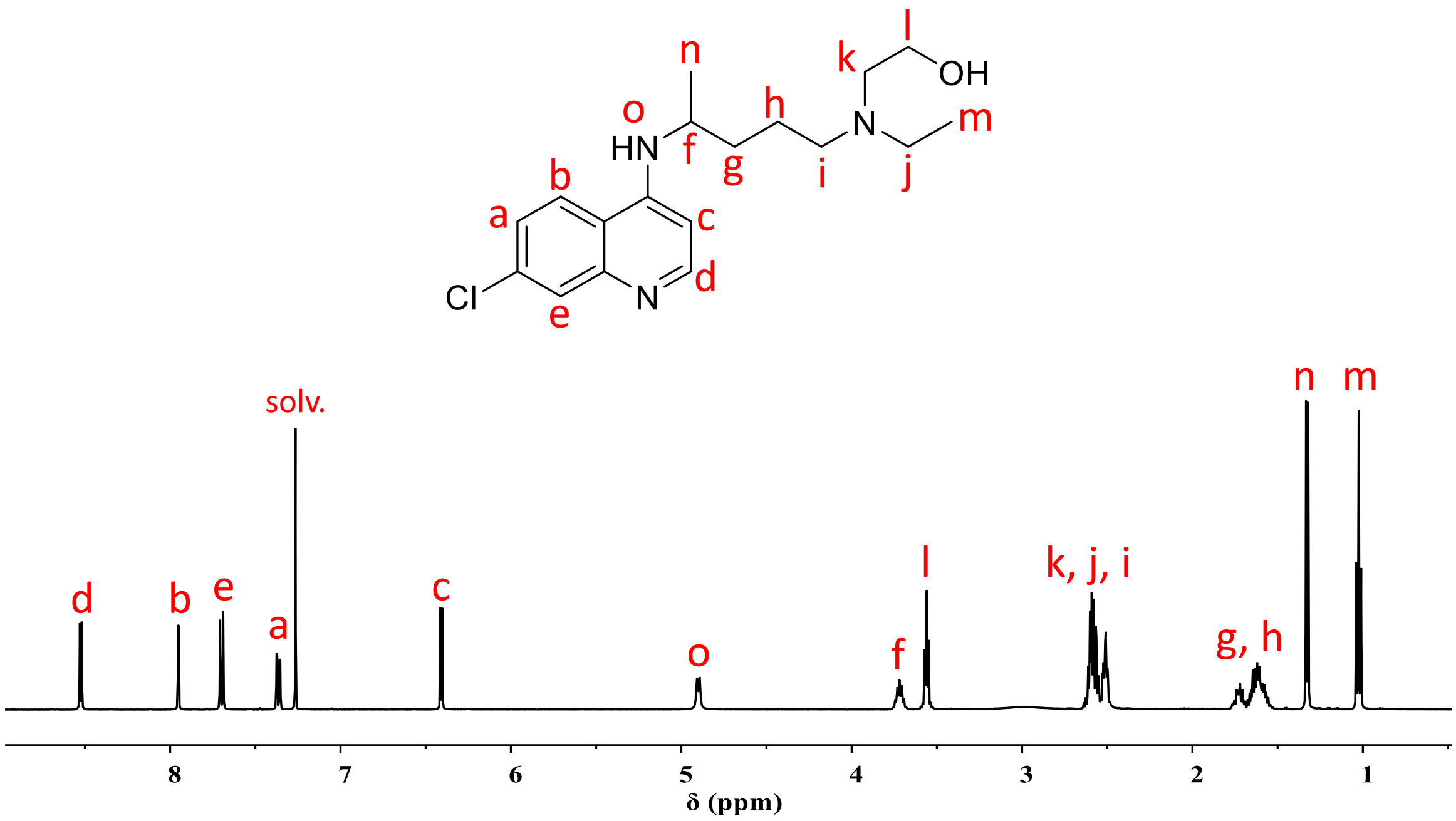


**Figure S7.** $^{1}$H NMR spectrum of HCQ in $CDCl_3$.

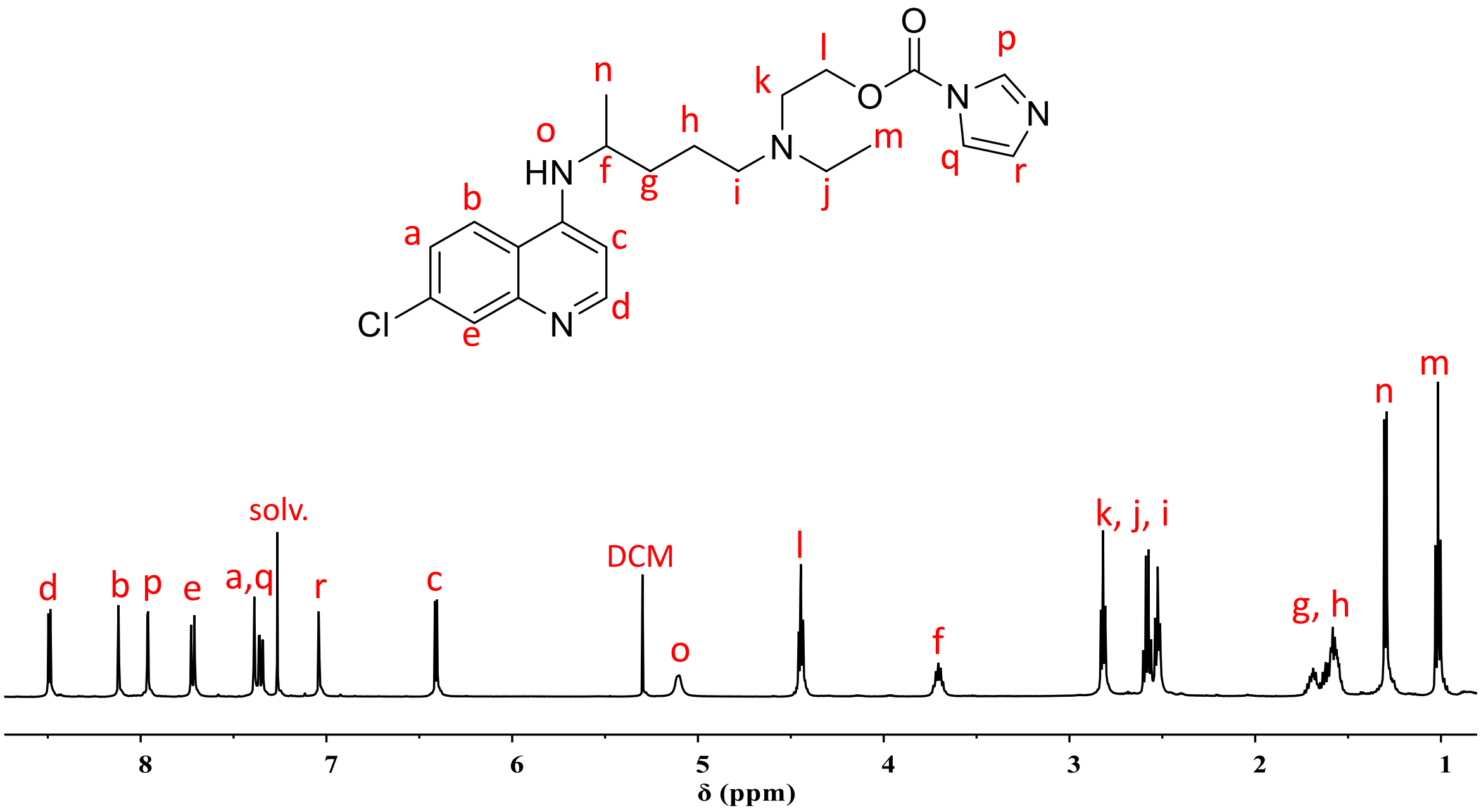


**Figure S8.** $^{1}$H NMR spectrum of HCQ-IMI in $CDCl_3$.

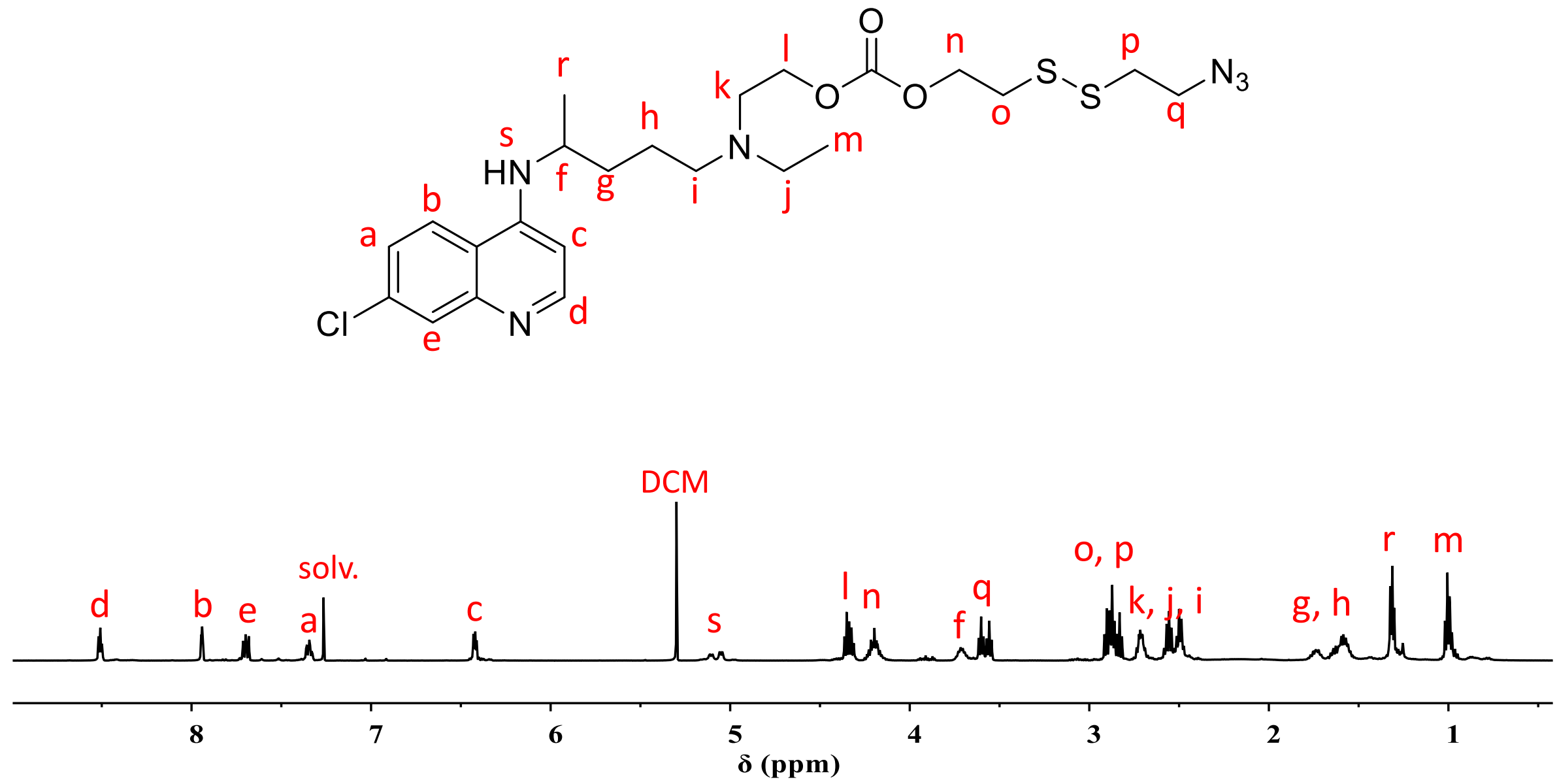


**Figure S9.** $^{1}$H NMR spectrum of HCQ-SS-$N_3$ in $CDCl_3$.

.

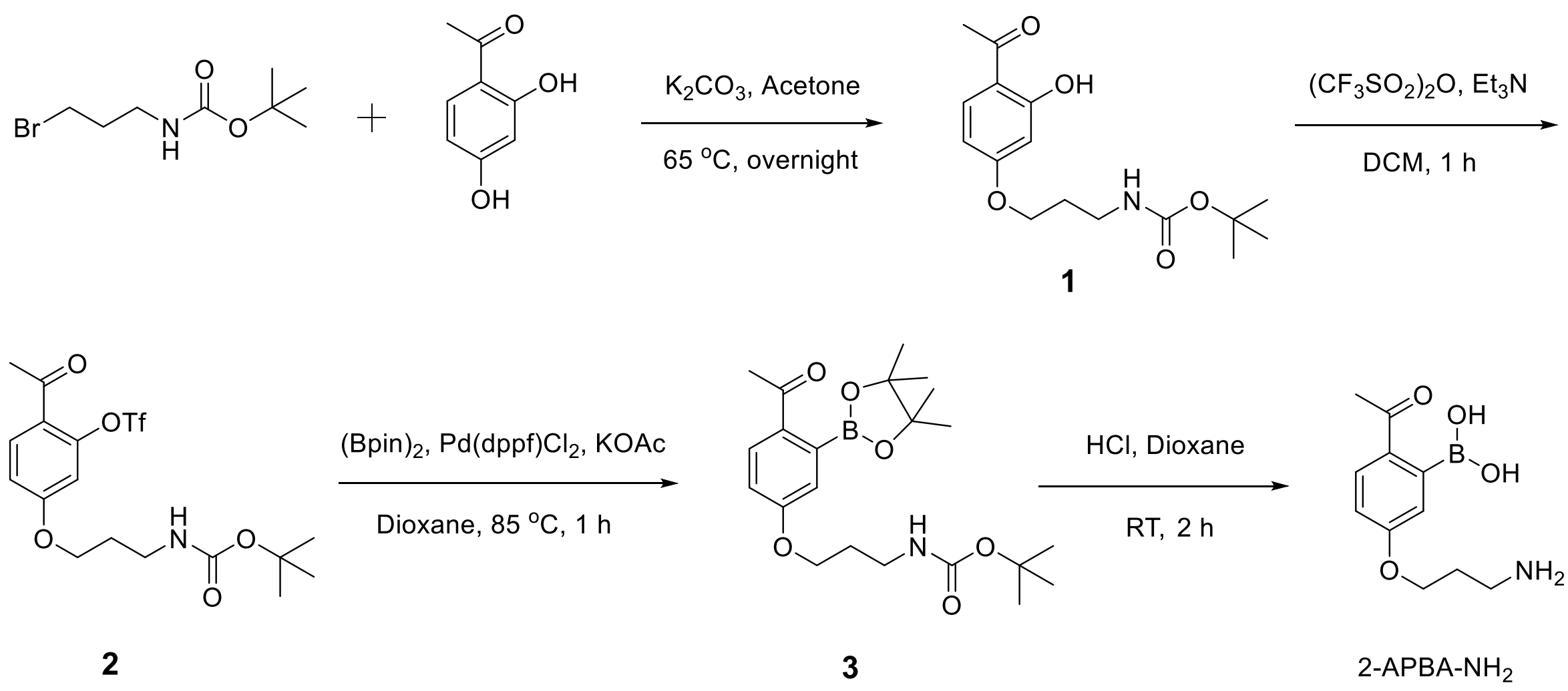


**Figure S10.** Synthetic route of 2-APBA-$NH_2$.

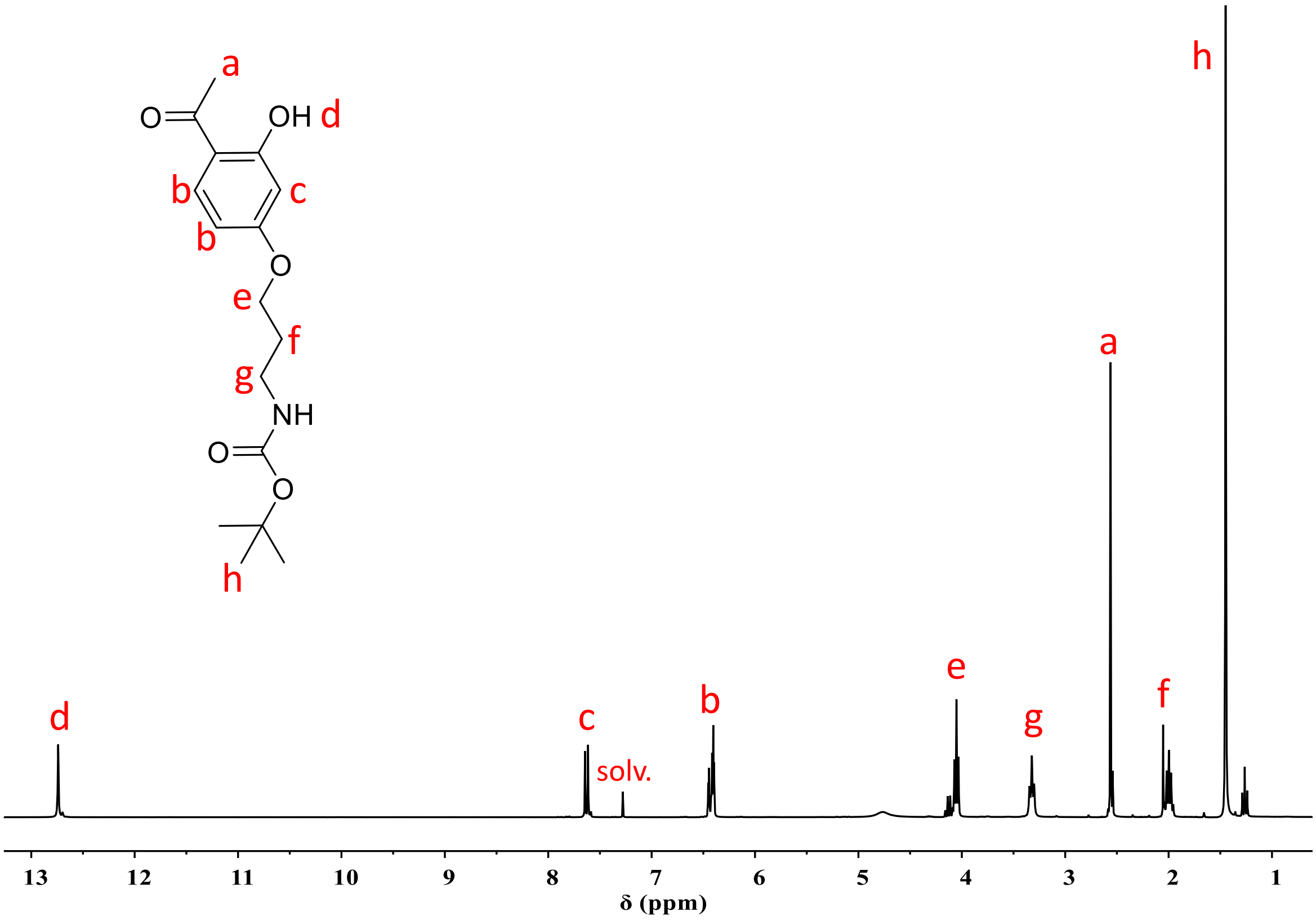


**Figure S11.** $^1$H NMR spectrum of intermediate **1** of 2-APBA-$NH_2$ in $CDCl_3$.

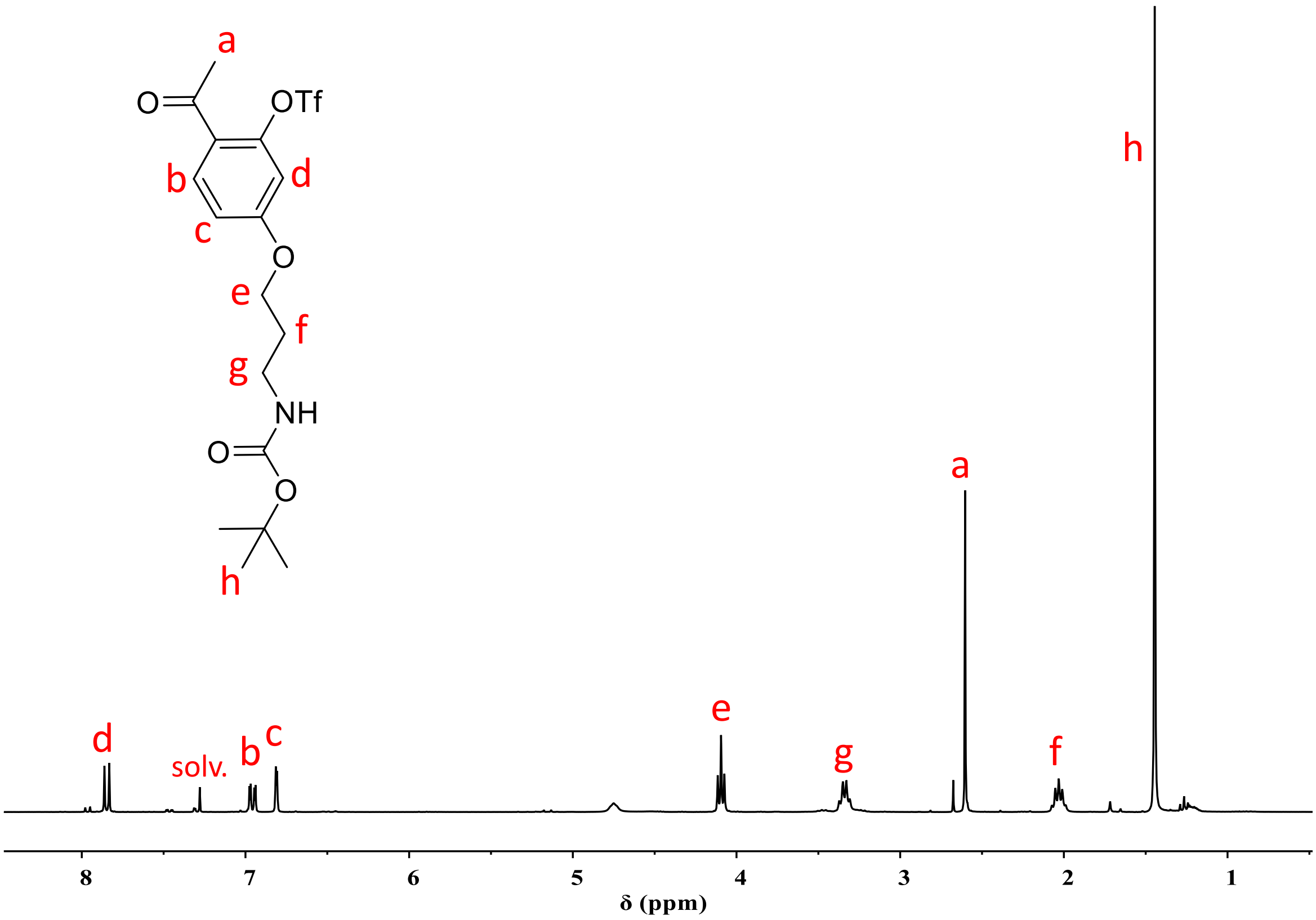


**Figure S12.** $^1$H NMR spectrum of intermediate **2** of 2-APBA-$NH_2$ in $CDCl_3$.

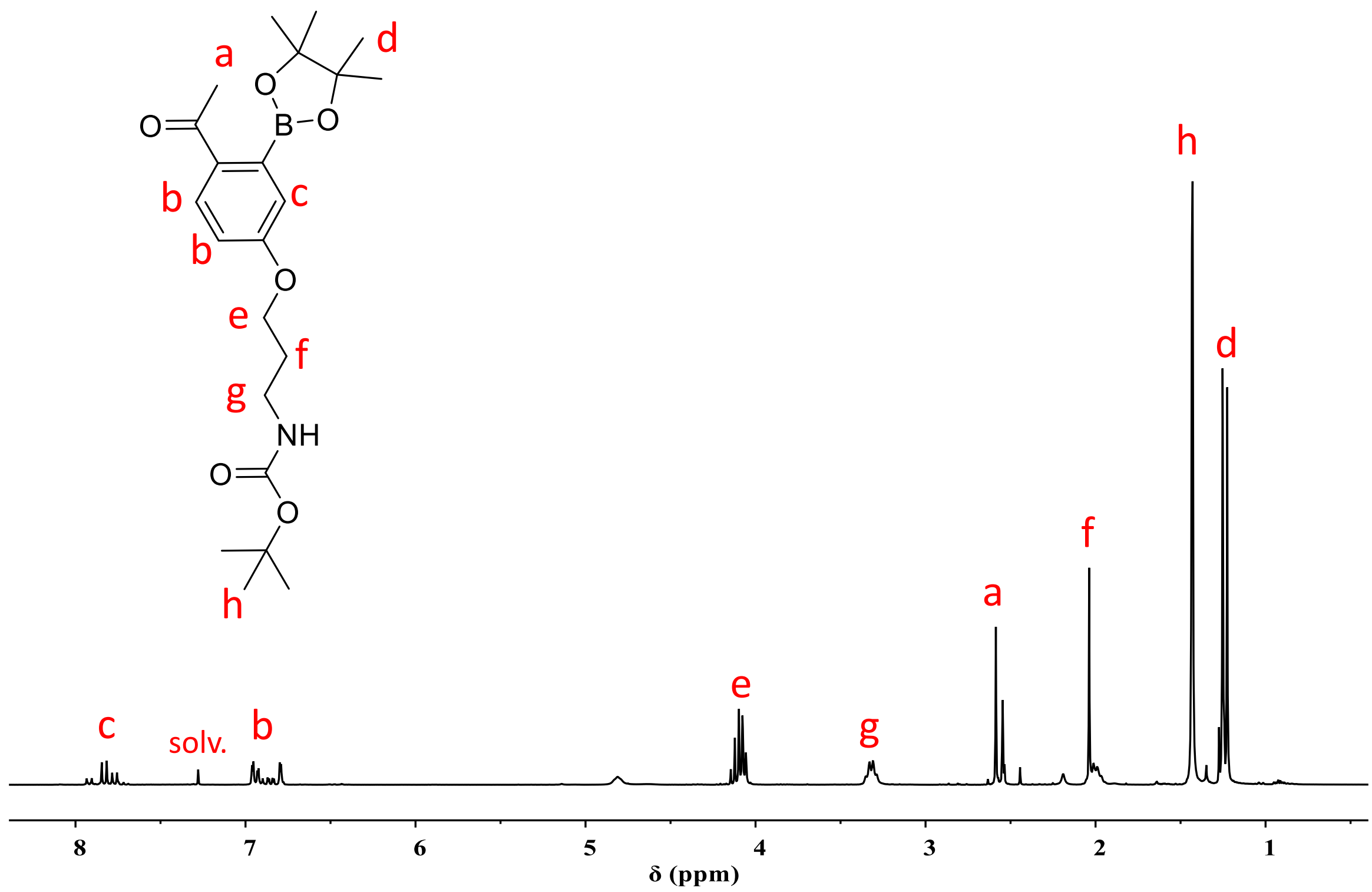


**Figure S13.** $^1H$ NMR spectrum of intermediate **3** of 2-APBA-$NH_2$ in $CDCl_3$.

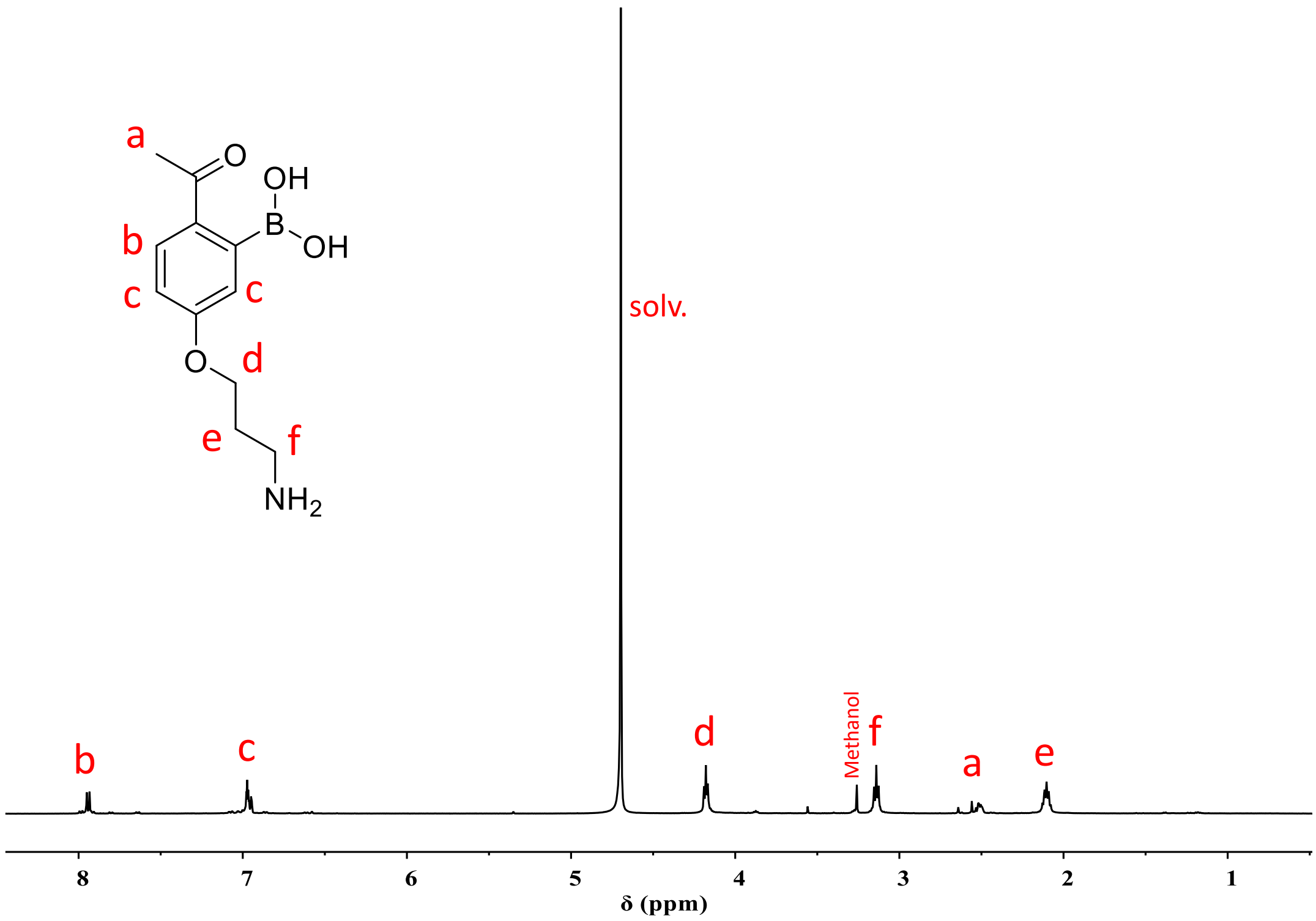


**Figure S14.** $^{1}$H NMR spectrum of 2-APBA-$NH_2$ in $D_2O$.

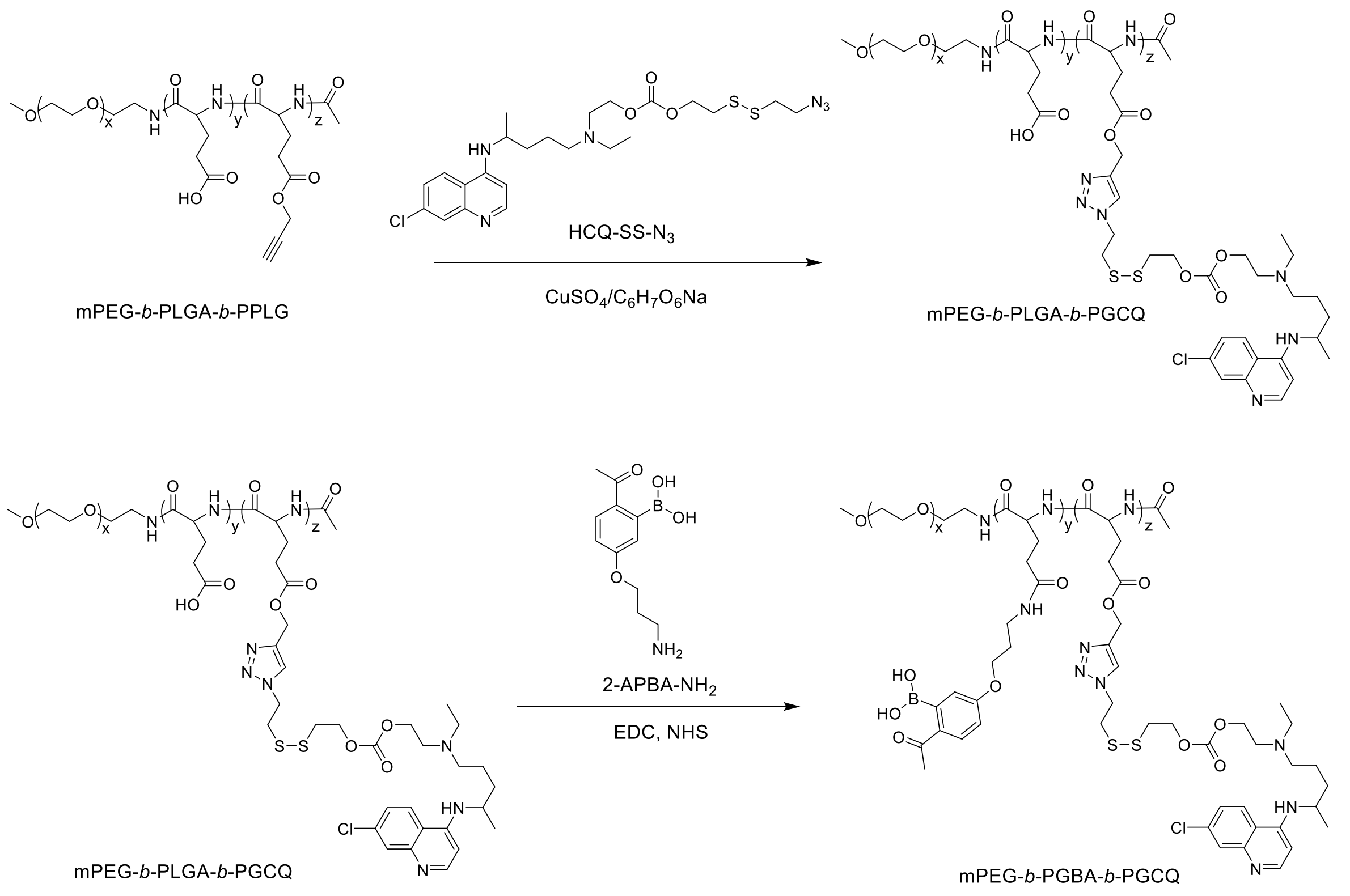


**Figure S15.** Synthetic route of mPEG-*b*-PGBA-*b*-PGCQ.

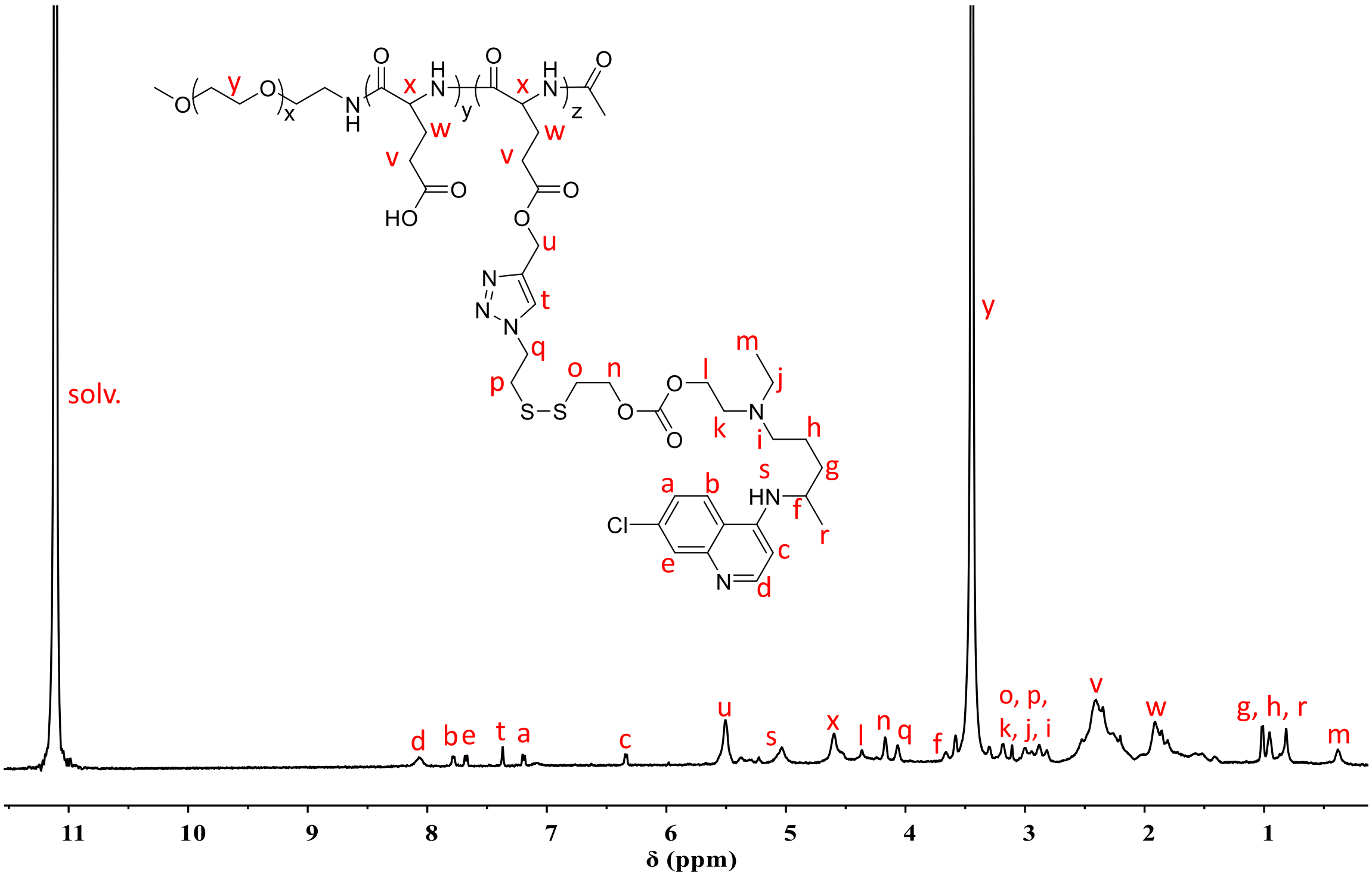


**Figure S16.** $^{1}$H NMR spectrum of mPEG-*b*-PLGA-*b*-PGCQ in $CF_3COOD$.

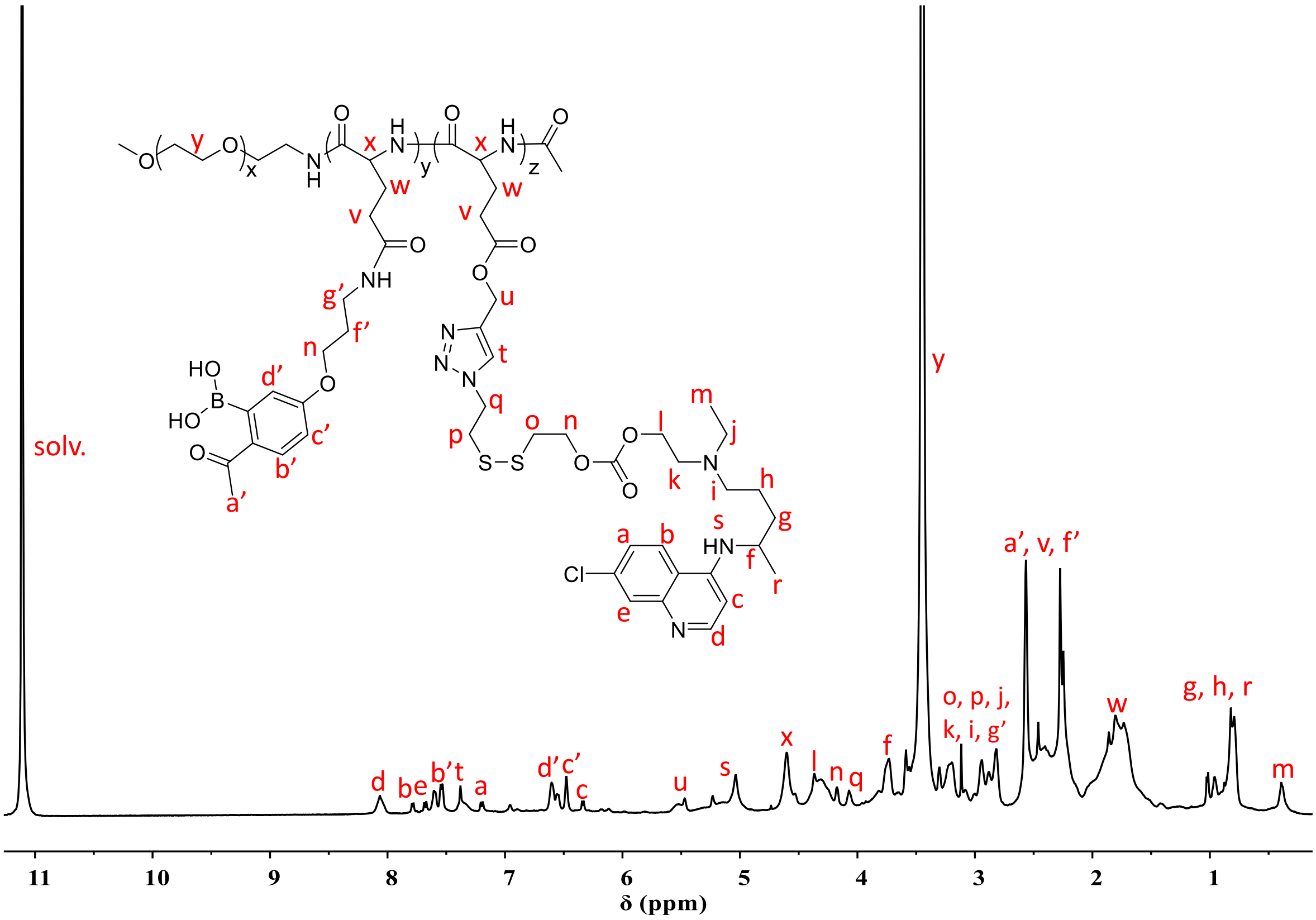


**Figure S17.** $^{1}$H NMR spectrum of mPEG-*b*-PGBA-*b*-PGCQ in $CF_3COOD$.

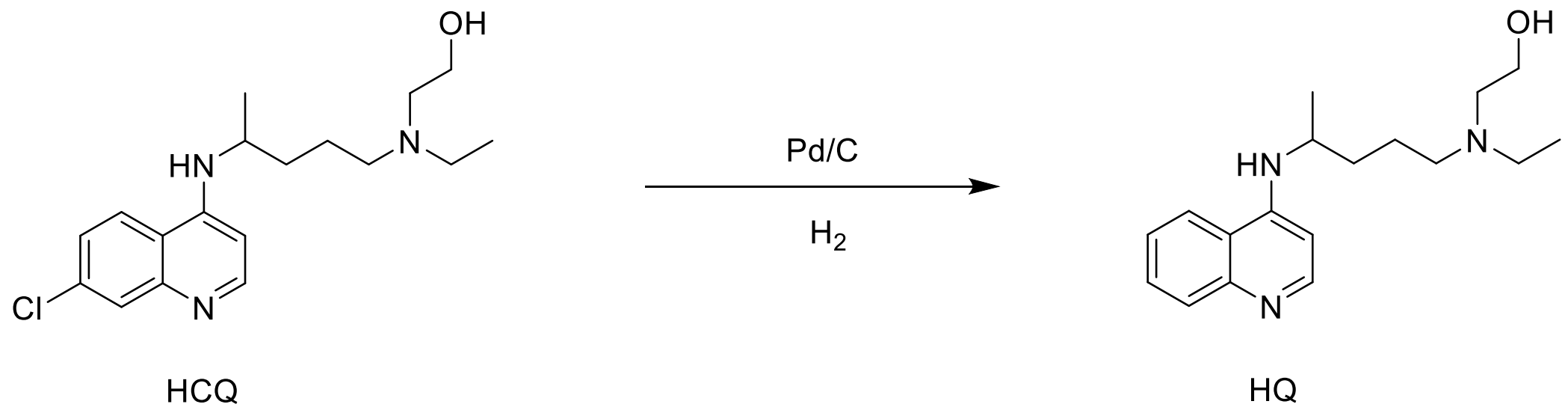


**Figure S18.** Synthetic route of HQ.

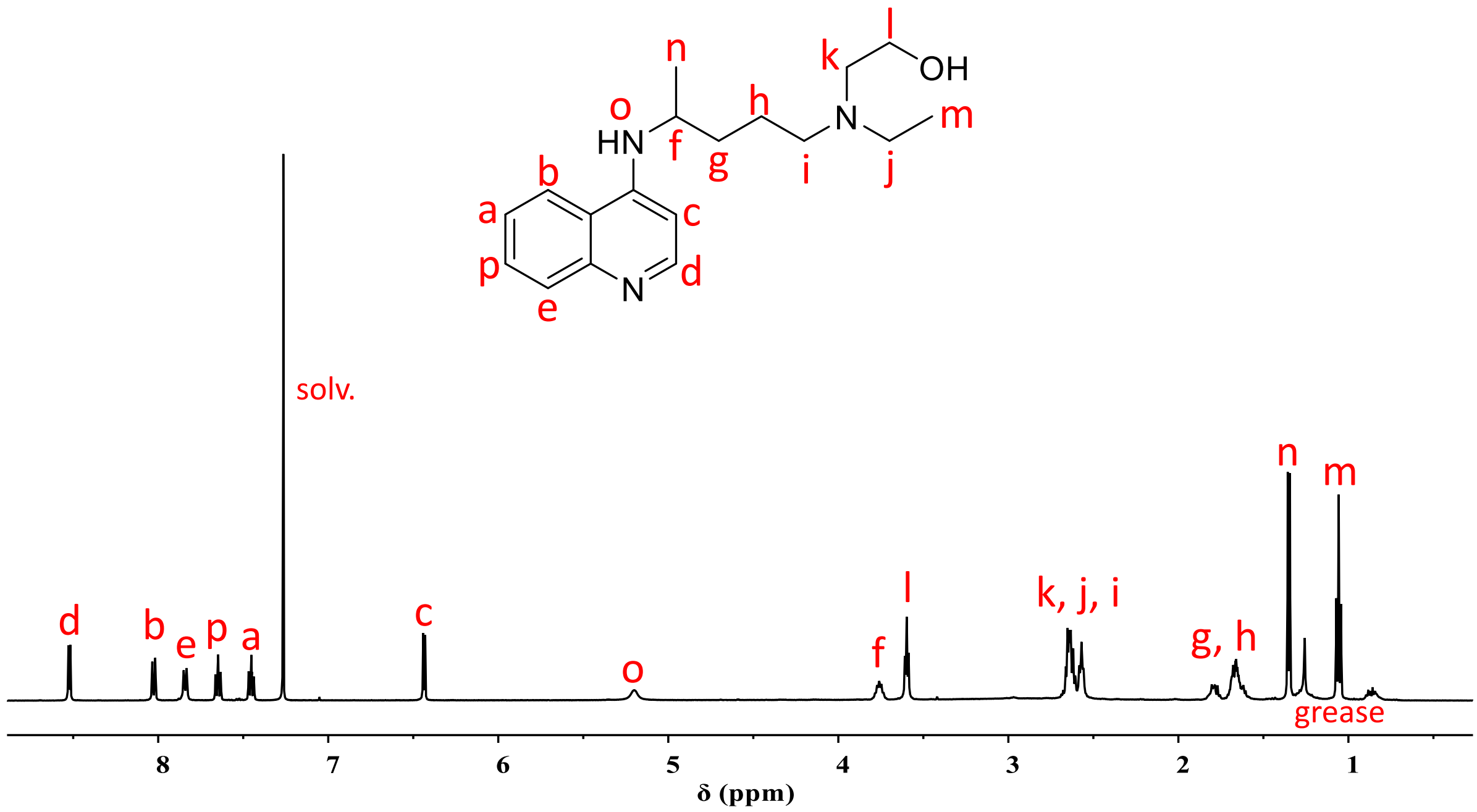


**Figure S19.** $^1$H NMR spectrum of HQ in $CDCl_3$.

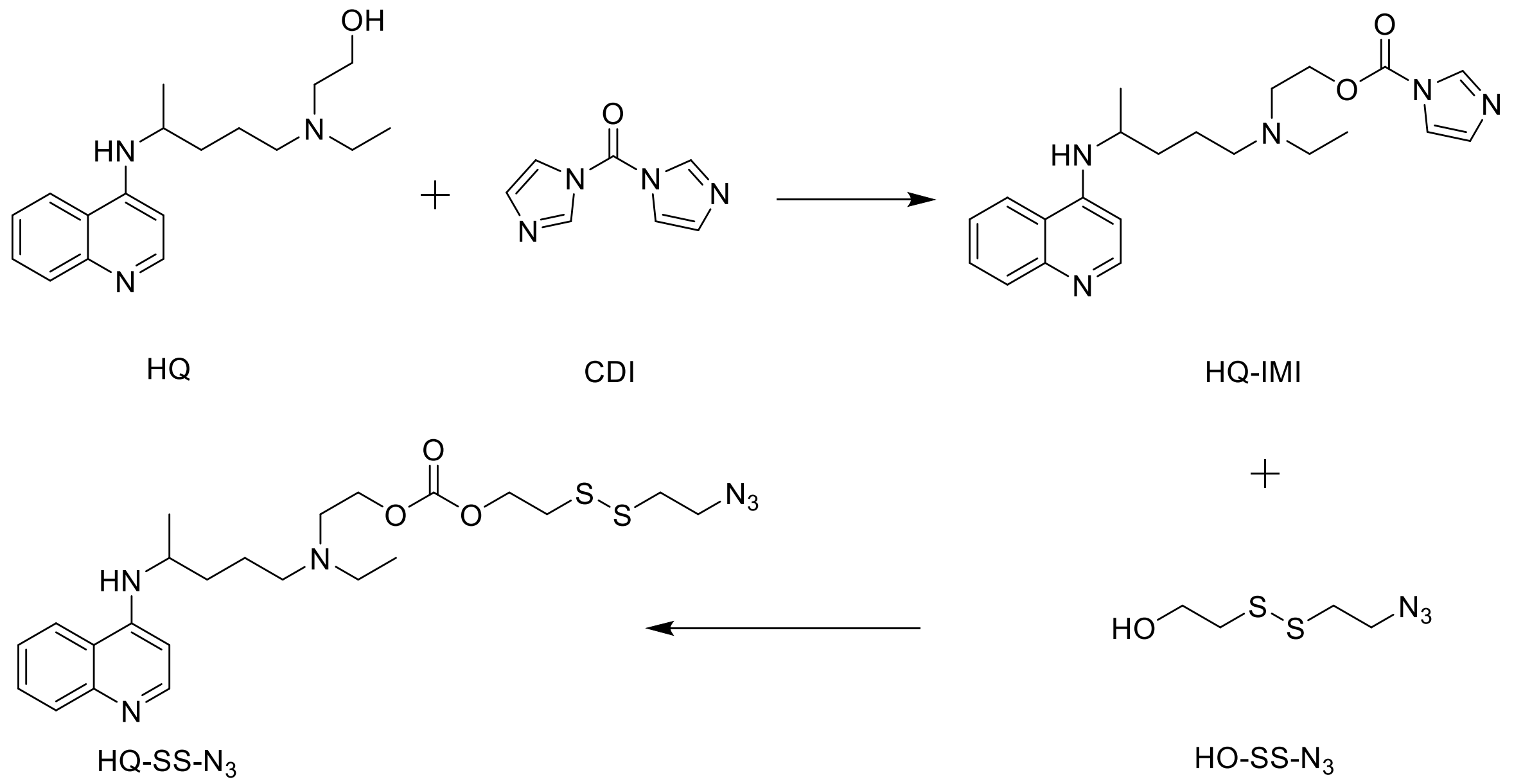


**Figure S20.** Synthetic route of HQ-$SS$-$N_3$.

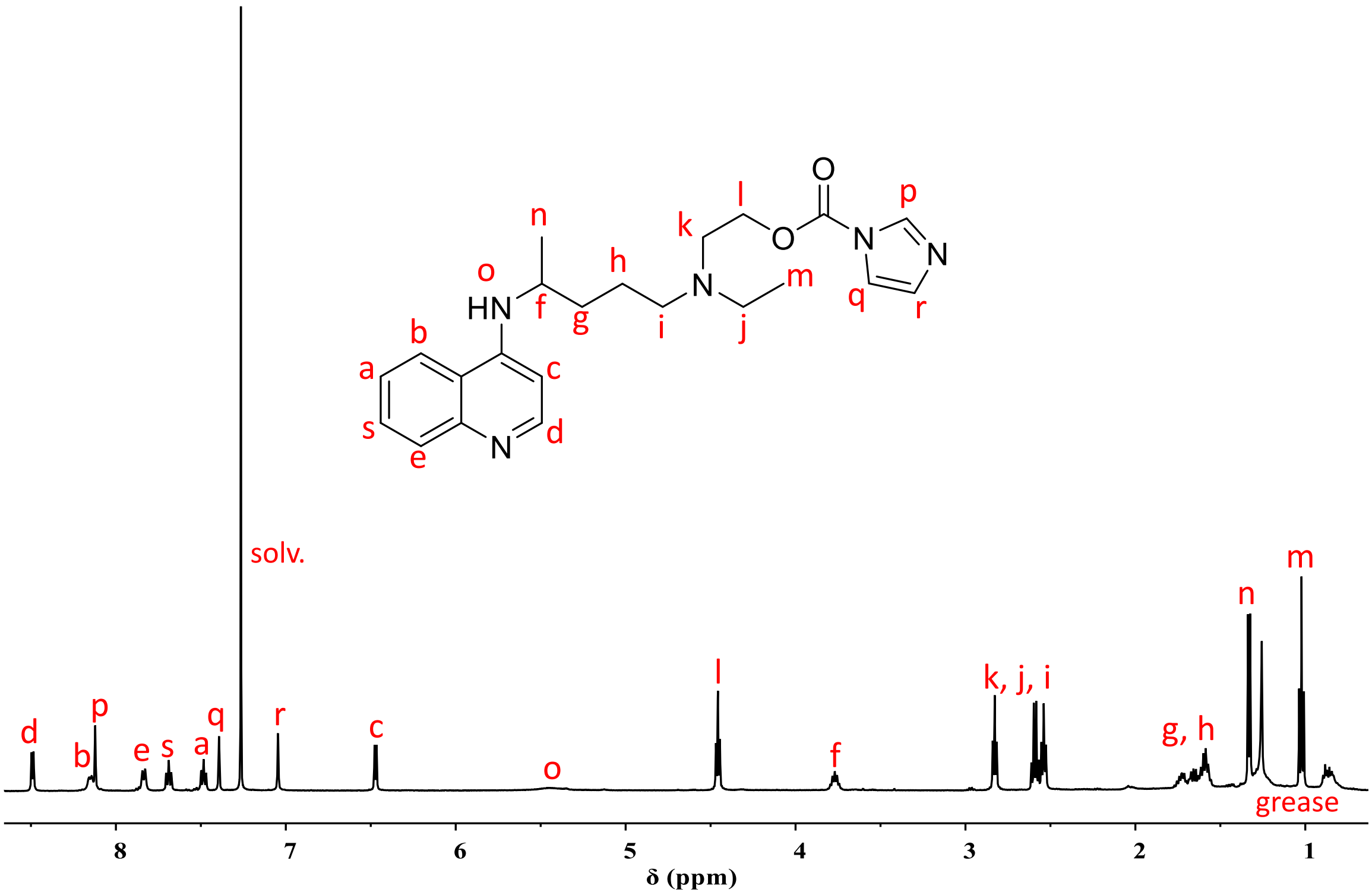


**Figure S21.** $^{1}$H NMR spectrum of HQ-IMI in $CDCl_3$.

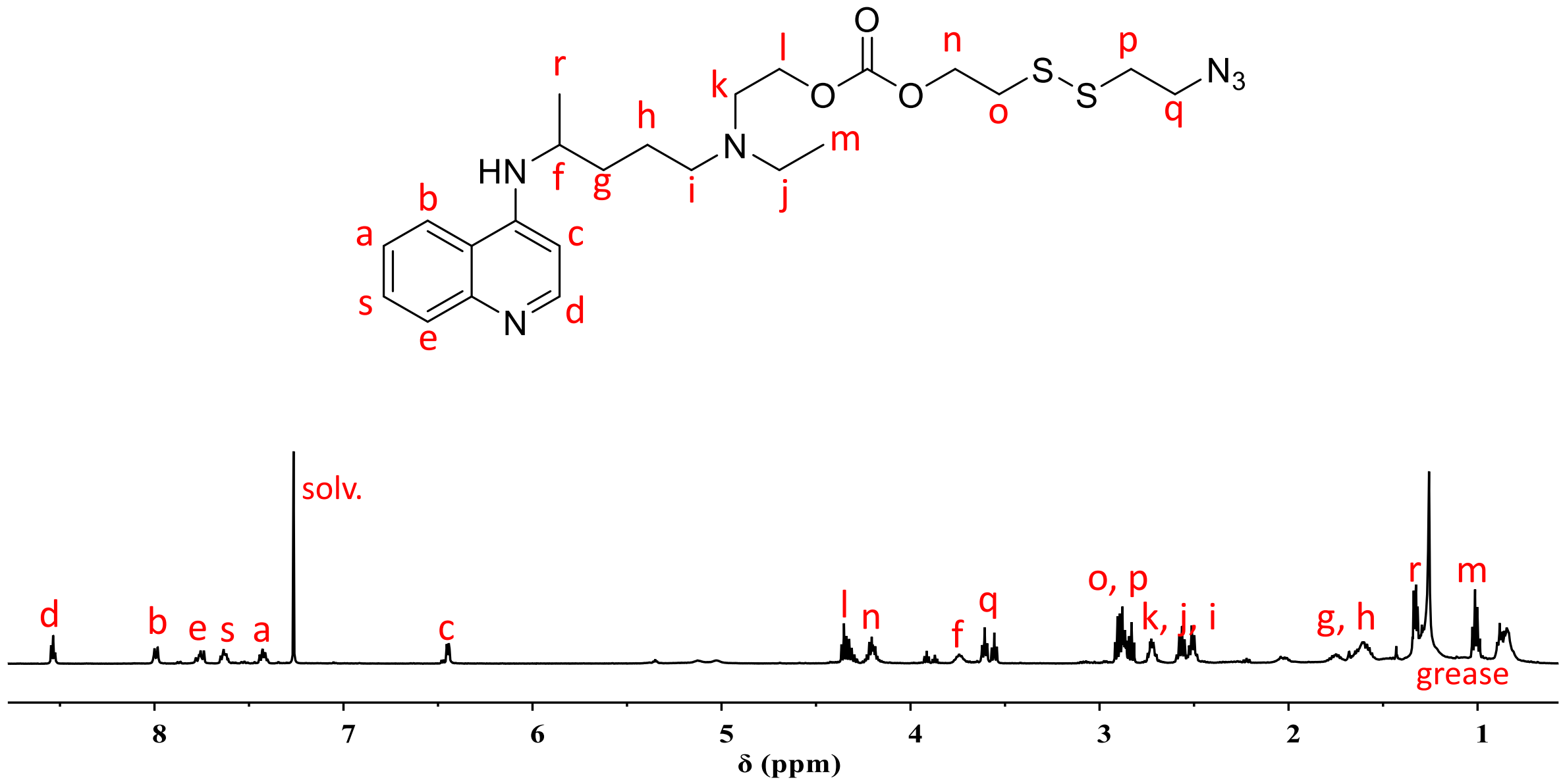


**Figure S22.** $^{1}$H NMR spectrum of HQ-SS-$N_3$ in $CDCl_3$.

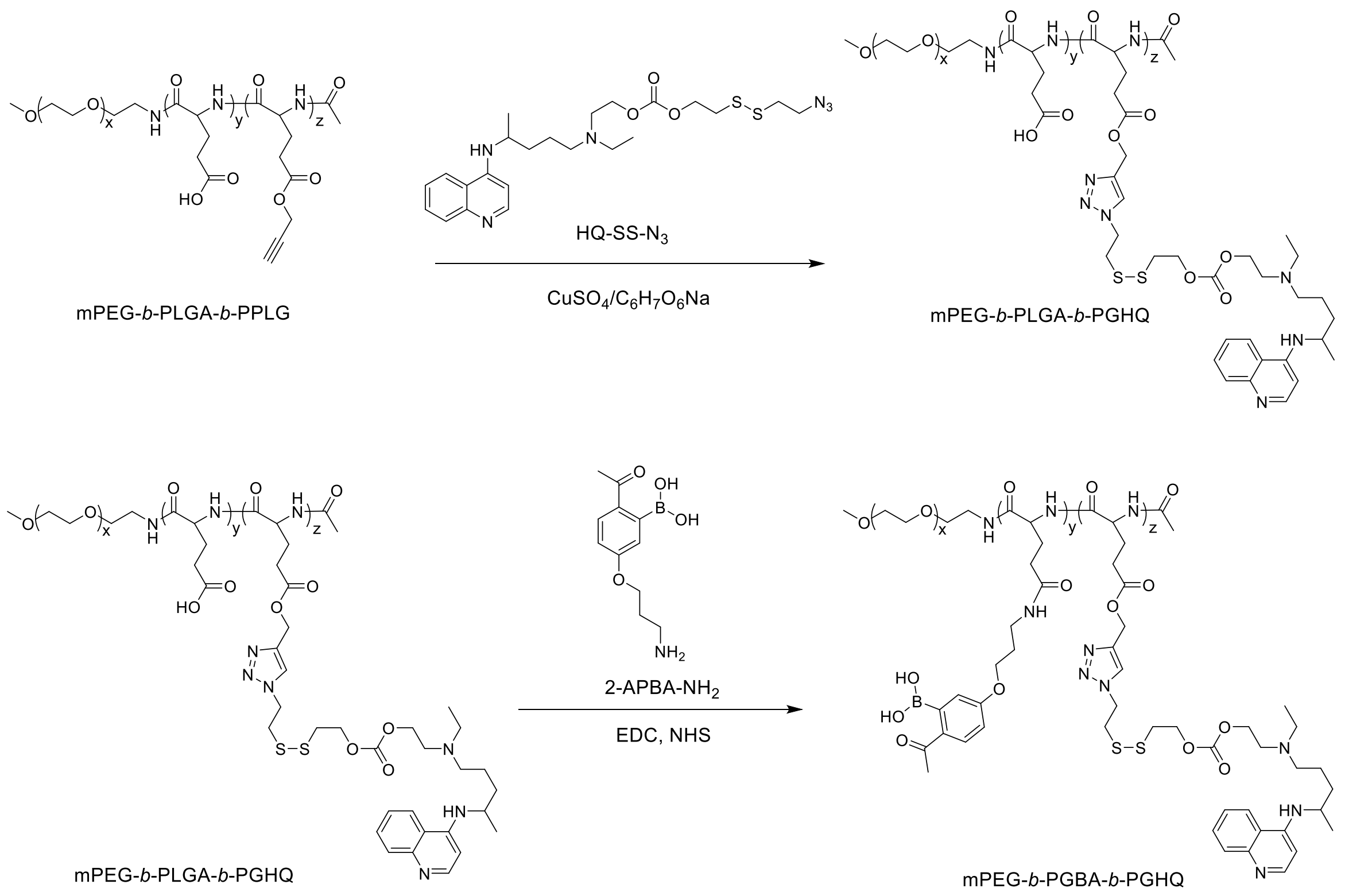


**Figure S23.** Synthetic route of mPEG-*b*-PGBA-*b*-PGHQ.

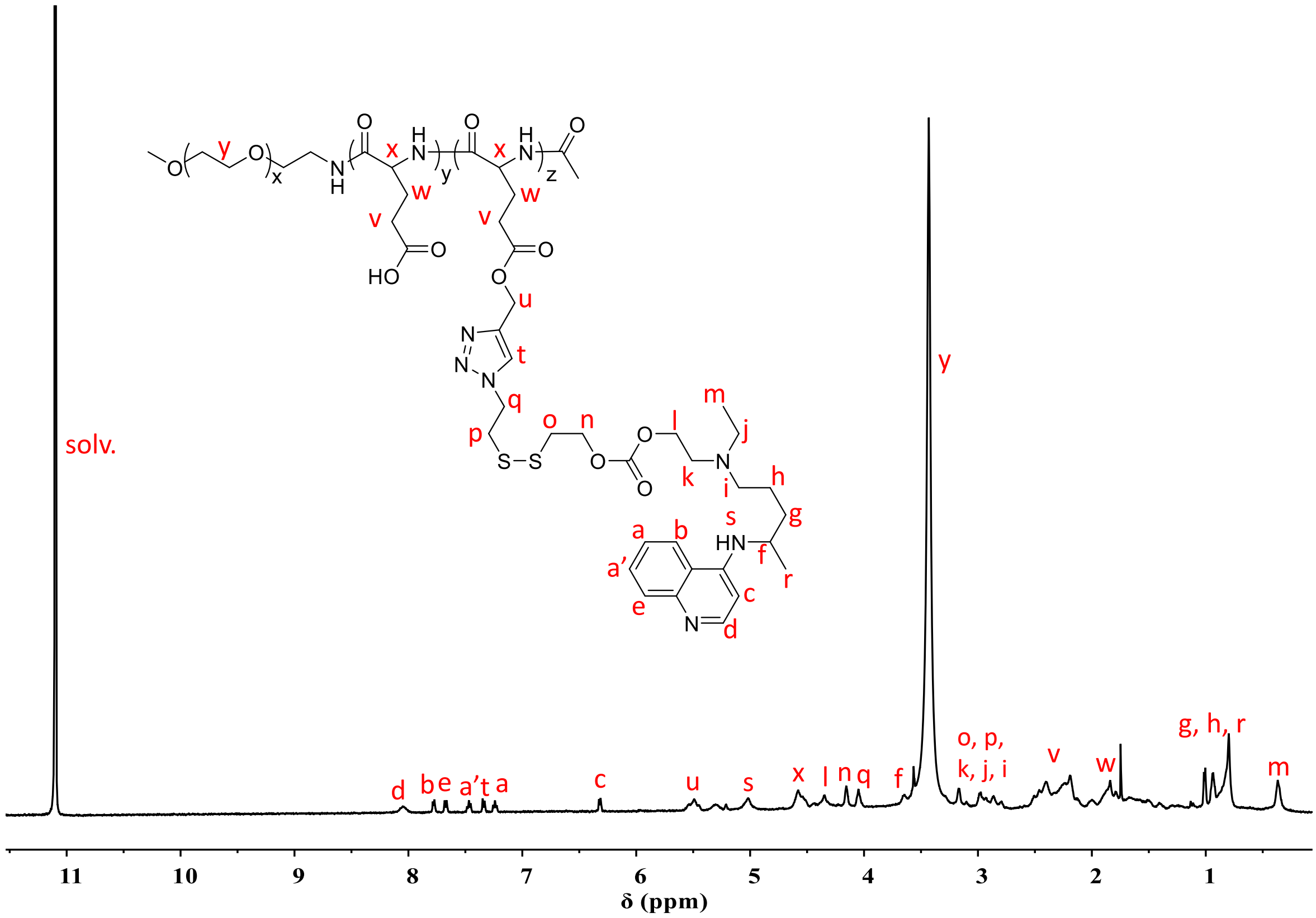


**Figure S24.** $^1$H NMR spectrum of mPEG-*b*-PLGA-*b*-PGHQ in $CF_3COOD$.

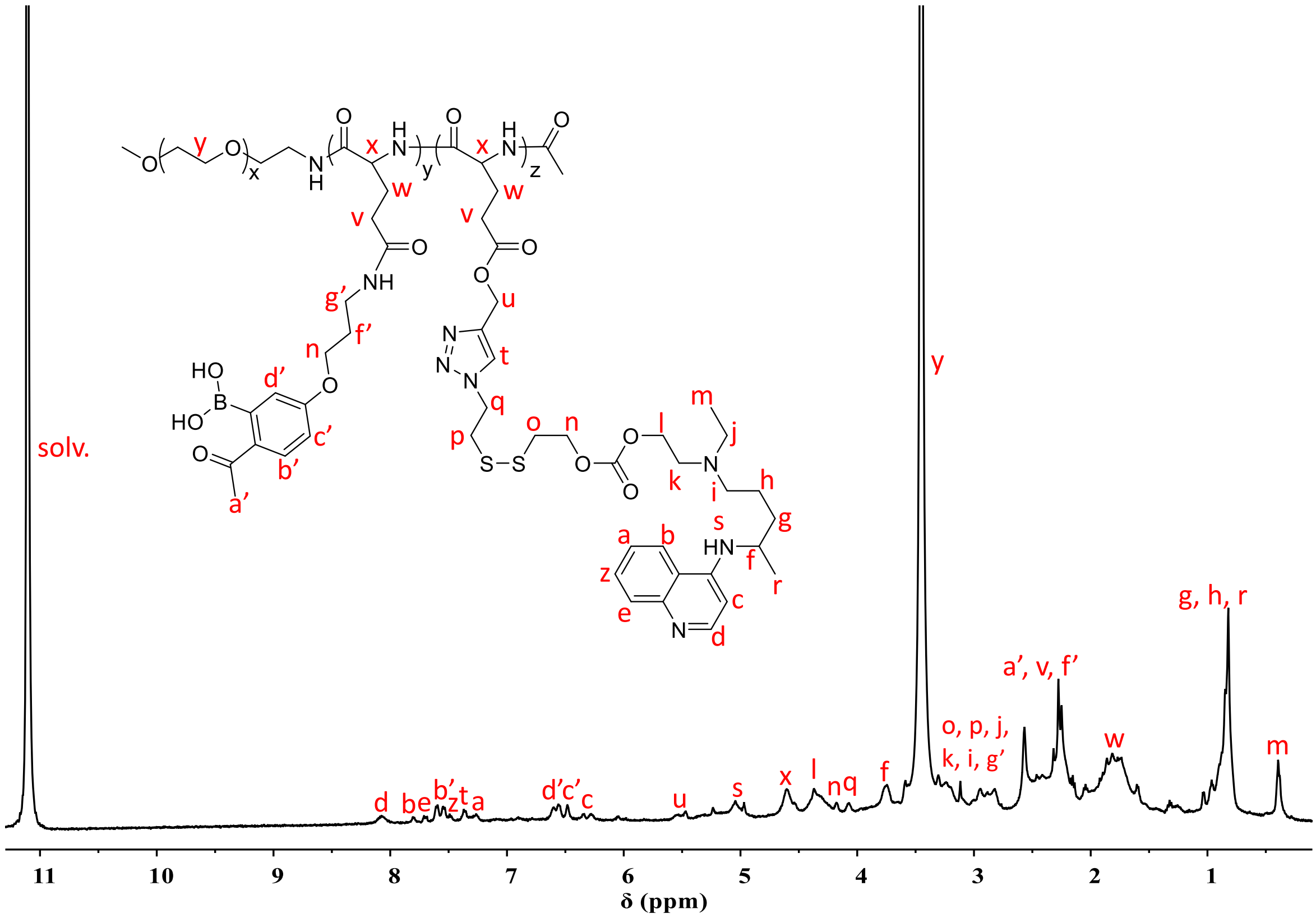


**Figure S25.** $^{1}H$ NMR spectrum of mPEG-*b*-PGBA-*b*-PGHQ in $CF_3COOD$.

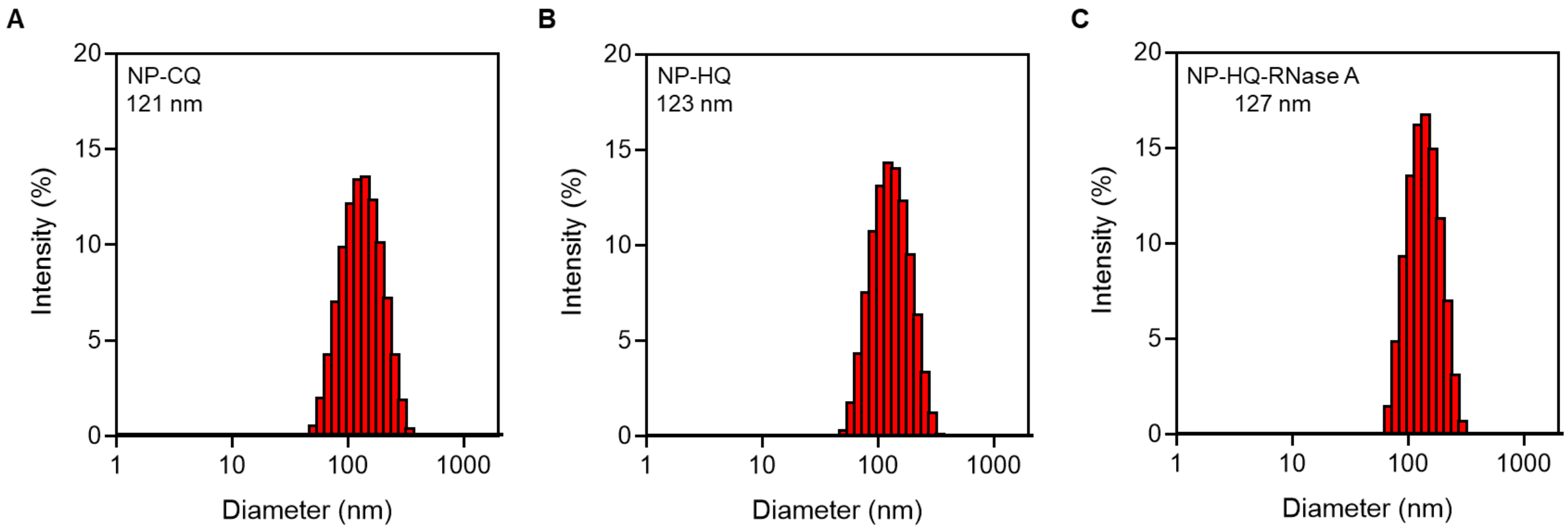


**Figure S26.** Particle size of NP-CQ (A), NP-HQ (B) and NP-HQ-RNase A (C) in PBS (pH 7.4) determined by DLS.

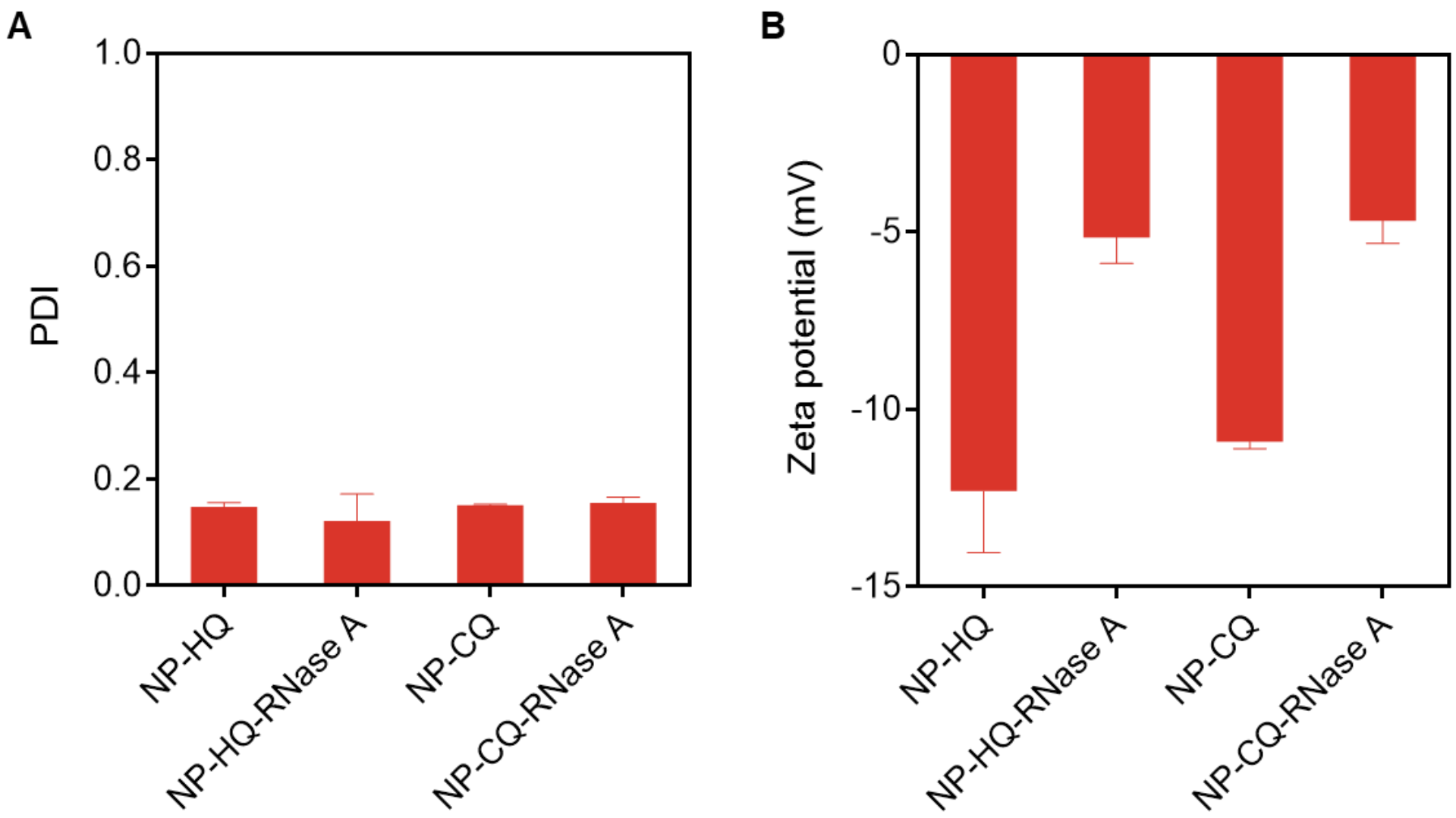


**Figure S27.** (A) PDI of NP-HQ, NP-HQ-RNase A, NP-CQ and NP-CQ-RNase A in PBS (pH 7.4) determined by DLS. (B) Zeta potential of NP-HQ, NP-HQ-RNase A, NP-CQ and NP-CQ-RNase A in PBS (pH 7.4) determined by DLS. Data are shown as mean ± SD (n = 3).

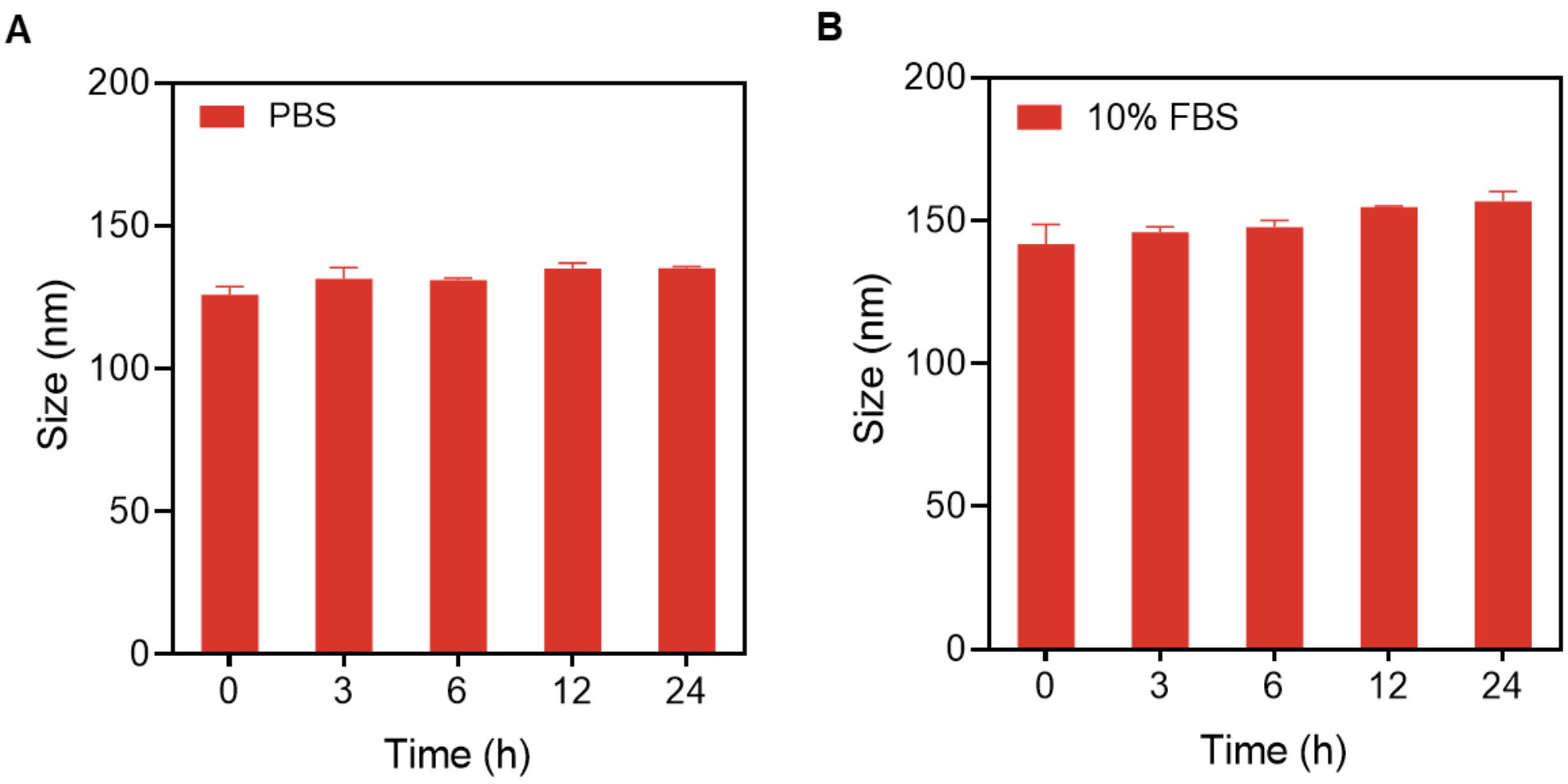


**Figure S28.** (A) Particle size of NP-CQ-RNase A in PBS (pH 7.4) at different times determined by DLS. (B) Particle size of NP-CQ-RNase A in PBS (pH 7.4) containing 10% FBS at different times determined by DLS. Data are shown as mean ± SD (n = 3).

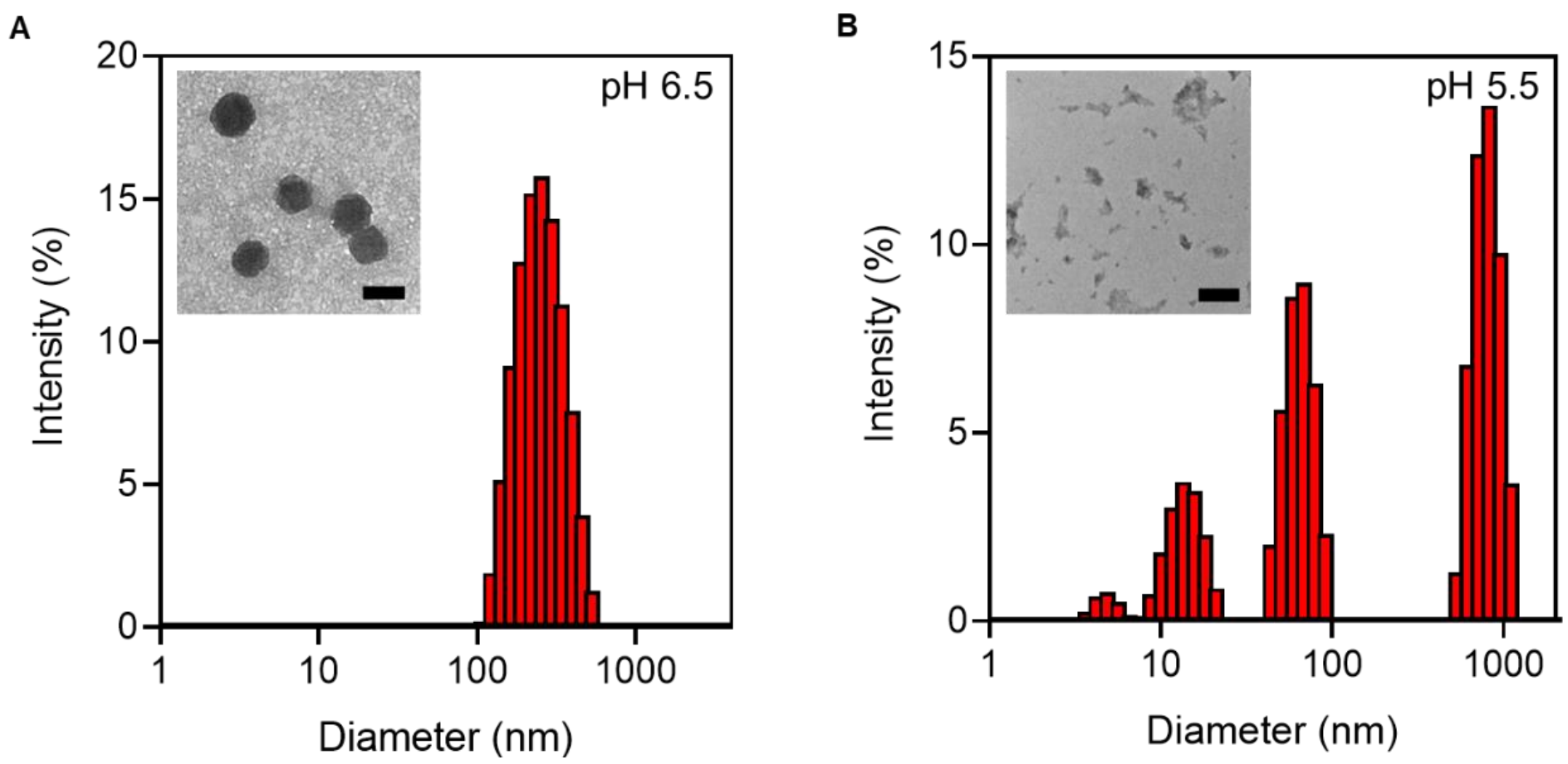


**Figure S29.** (A) DLS measurement and TEM image of NP-CQ-RNase A at pH 6.5. (B) DLS measurement and TEM image of NP-CQ-RNase A at pH 5.5. Scale bar = 200 nm.

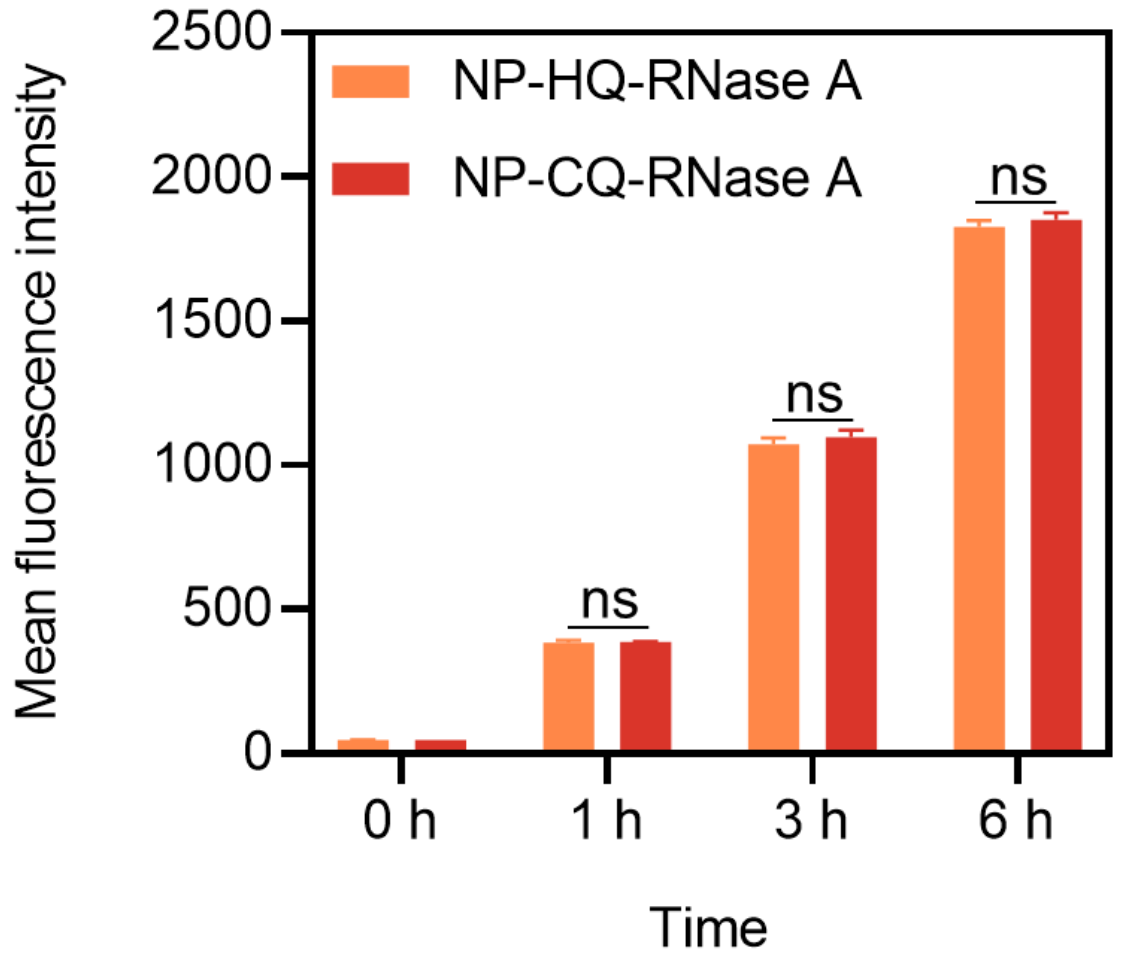


**Figure S30.** Flow cytometry analyses of cell uptake of NP-HQ-RNase A-Cy5 and NP-CQ-RNase A-Cy5 (10 µg/mL RNase A) by 4T1 cells after different incubation times. Data are shown as mean ± SD (n = 3). ns: not significant.

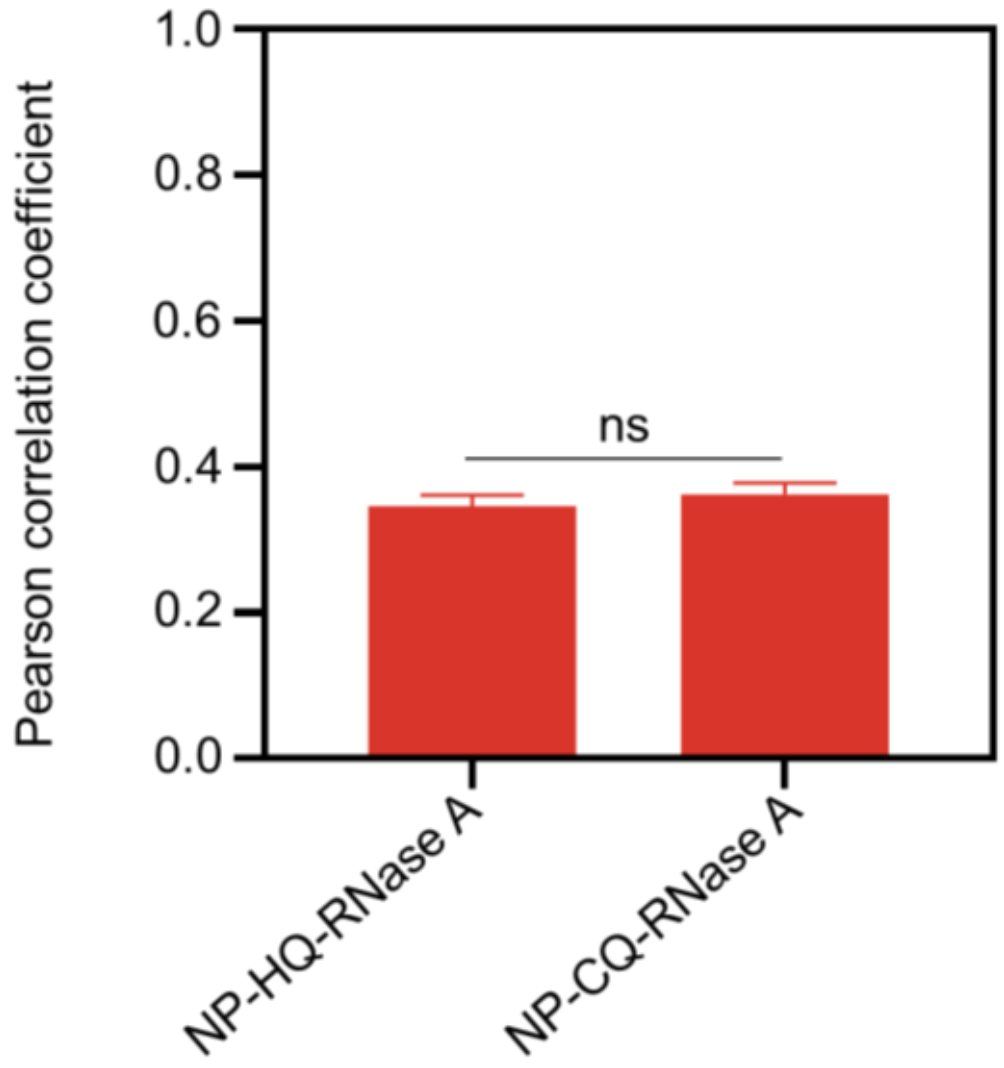


**Figure S31**. Quantitative Pearson correlation coefficients (lysosome-RNase A colocalization) of NP-HQ-RNase A and NP-CQ-RNase A groups corresponding to Figure 2B. Data are shown as mean ± SD (n = 3). ns: not significant.

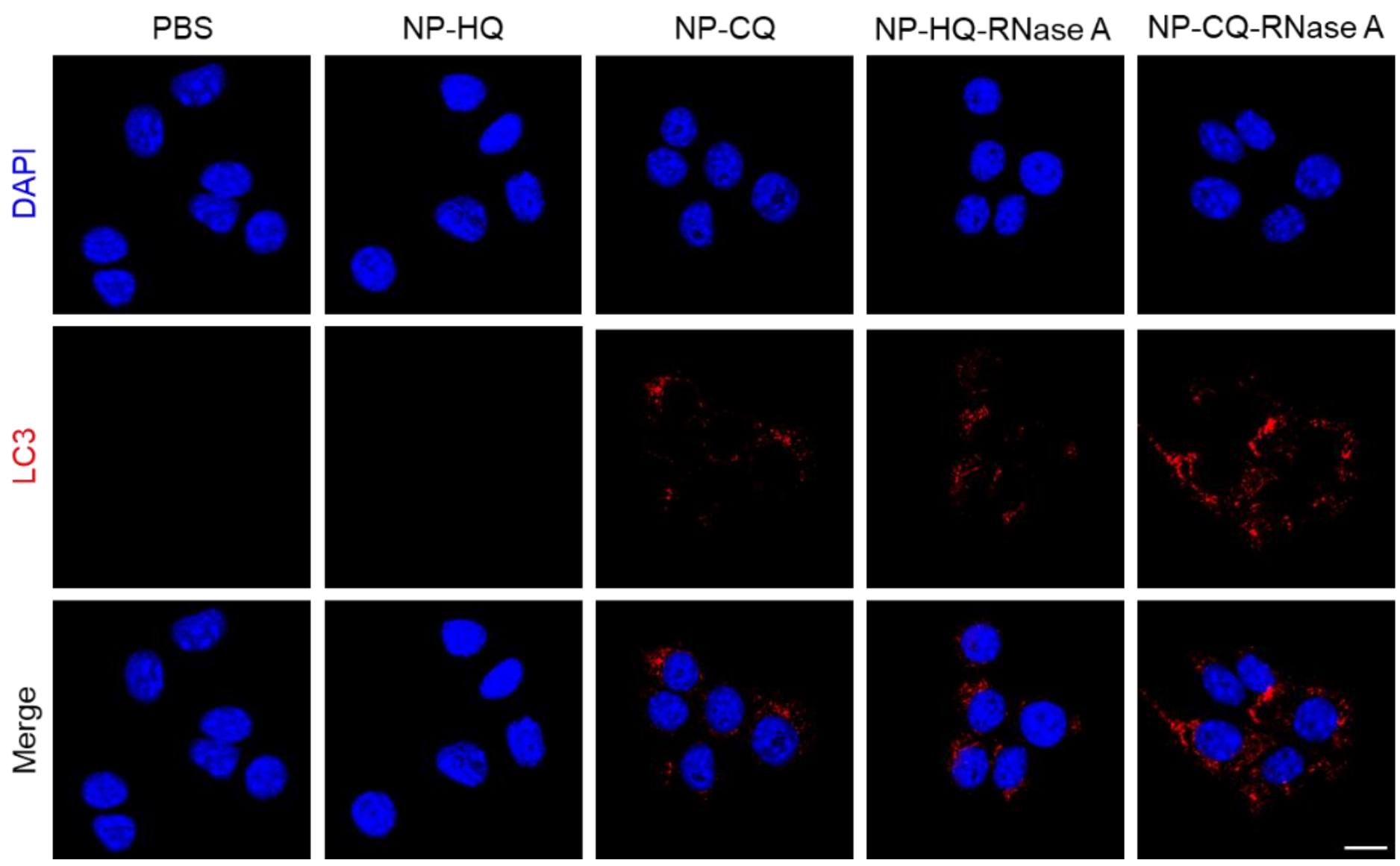


**Figure S32**. Representative immunofluorescence micrographs of LC3 puncta in 4T1 cells exposed to the indicated formulations (10 μg/mL RNase A) for 24 h. Scale bar = 10 μm.

.

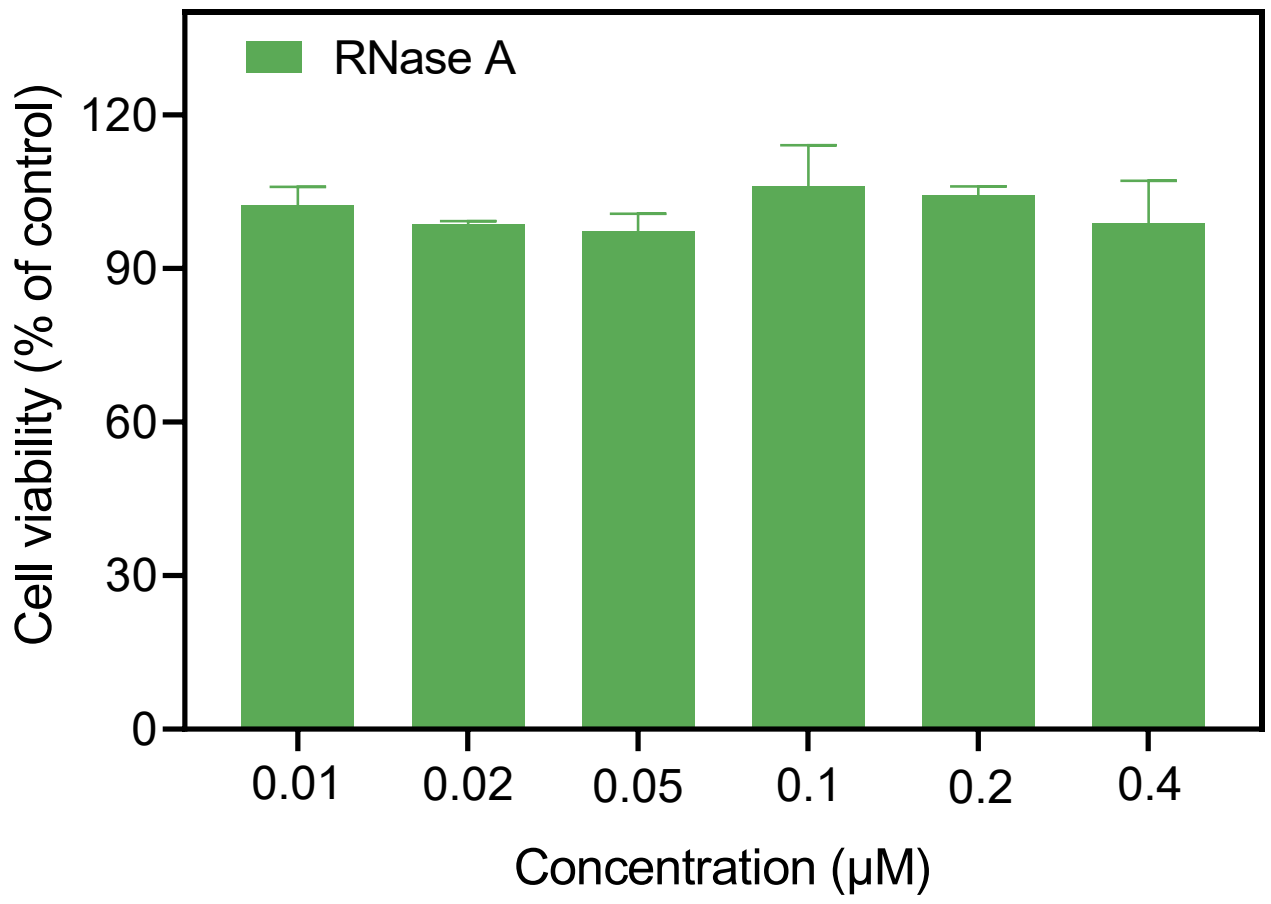


**Figure S33.** *In vitro* cytotoxicity of RNase A against 4T1 cells as determined by MTT assay. Cells were incubated with RNase A for 72 h at 37 °C. Data are shown as mean ± SD (n = 3).

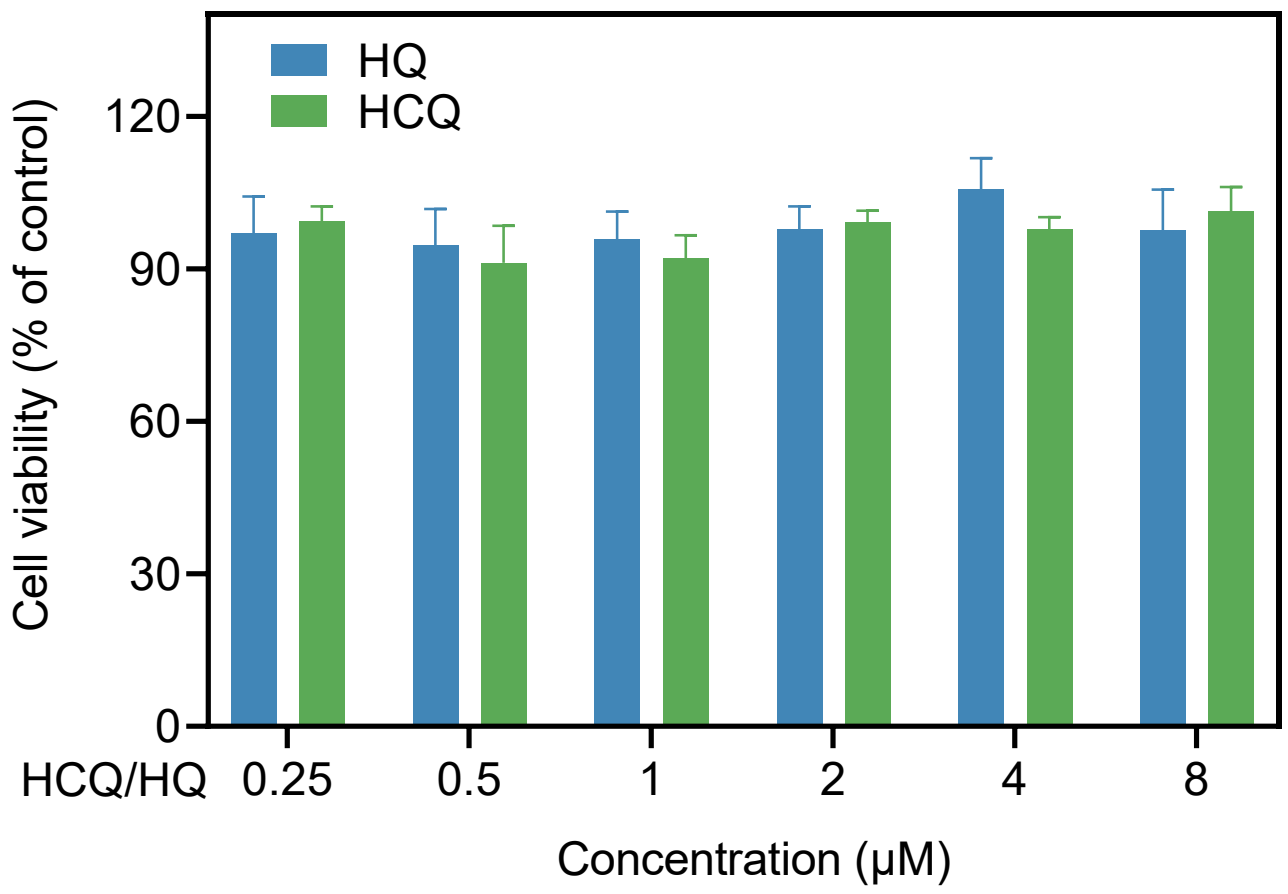

**Figure S34.** *In vitro* cytotoxicity of HQ and HCQ against 4T1 cells as determined by MTT assay. Cells were incubated with the indicated agents for 72 h at 37 °C. Data are shown as mean ± SD (n = 3).

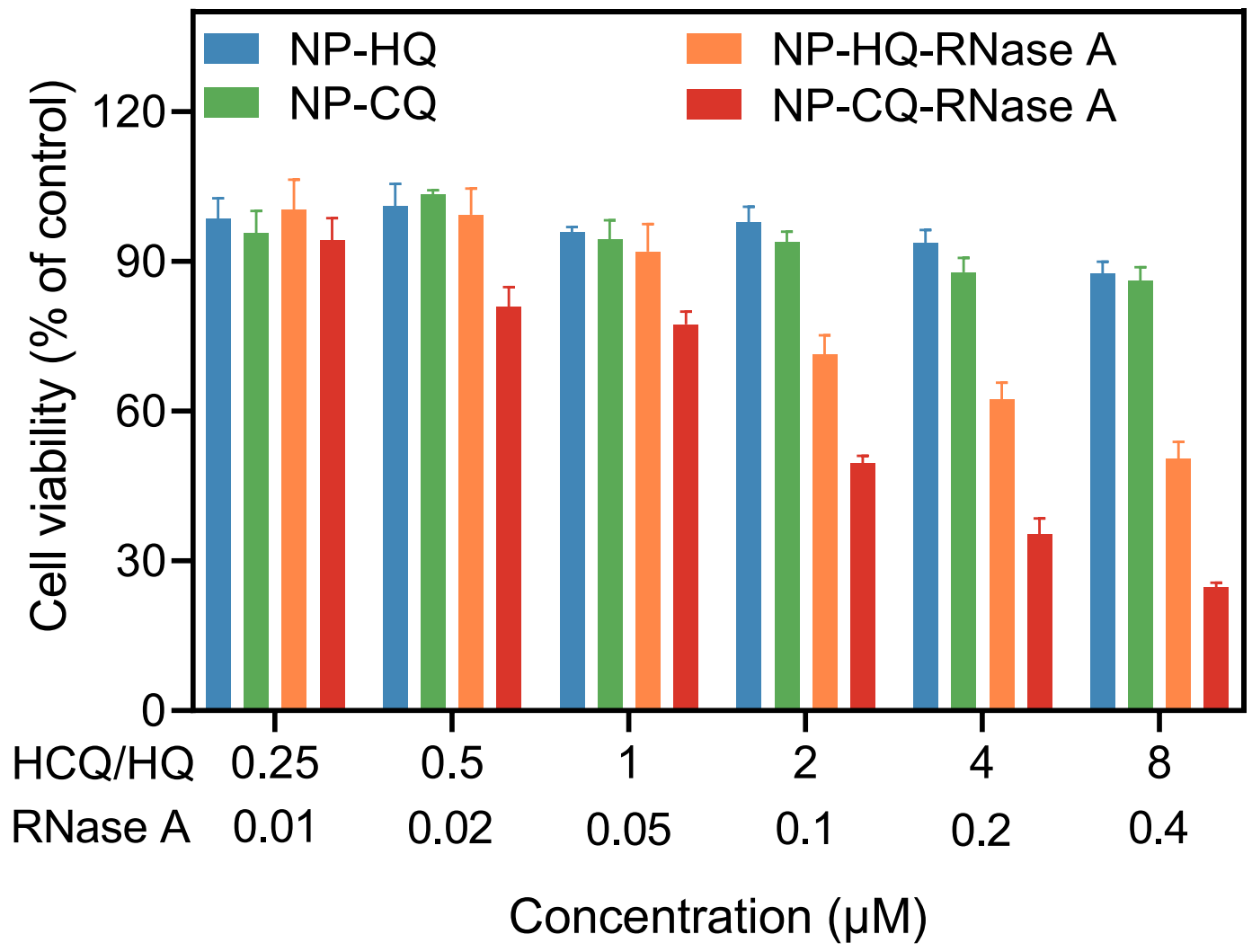


**Figure S35.** *In vitro* cytotoxicity of NP-HQ, NP-HQ-RNase A, NP-CQ and NP-CQ-RNase A against 4T1 cells as determined by MTT assay. Cells were incubated with the indicated agents for 72 h at 37 °C. Data are shown as mean ± SD (n = 3).

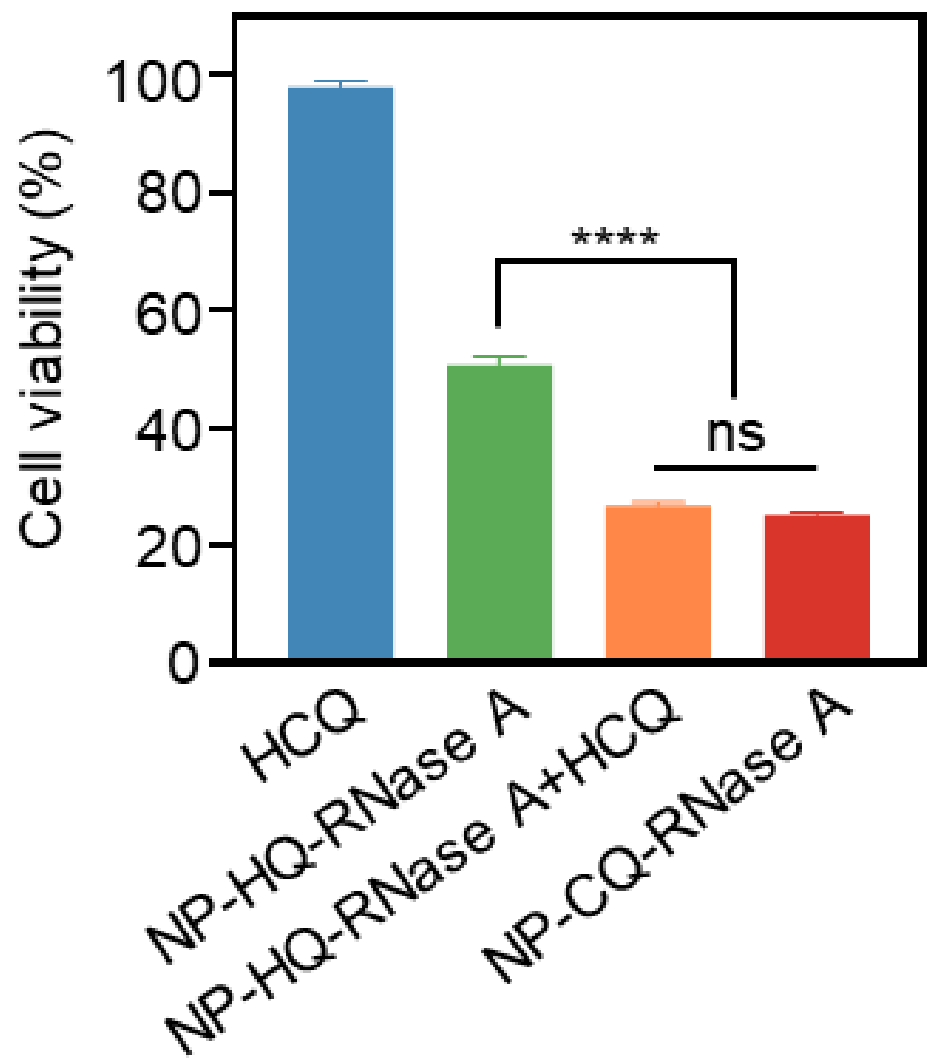


**Figure S36.** Cell viability of 4T1 cells exposed to HCQ, NP-HQ-RNase A, NP-HQ-RNase A+HCQ, and NP-CQ-RNase A (0.4 μM RNase A, 8 μM HCQ/HQ) for 72 h at 37 °C as determined by MTT assay. ****$p < 0.001$, ns: not significant. Data are shown as mean ± SD (n = 3).

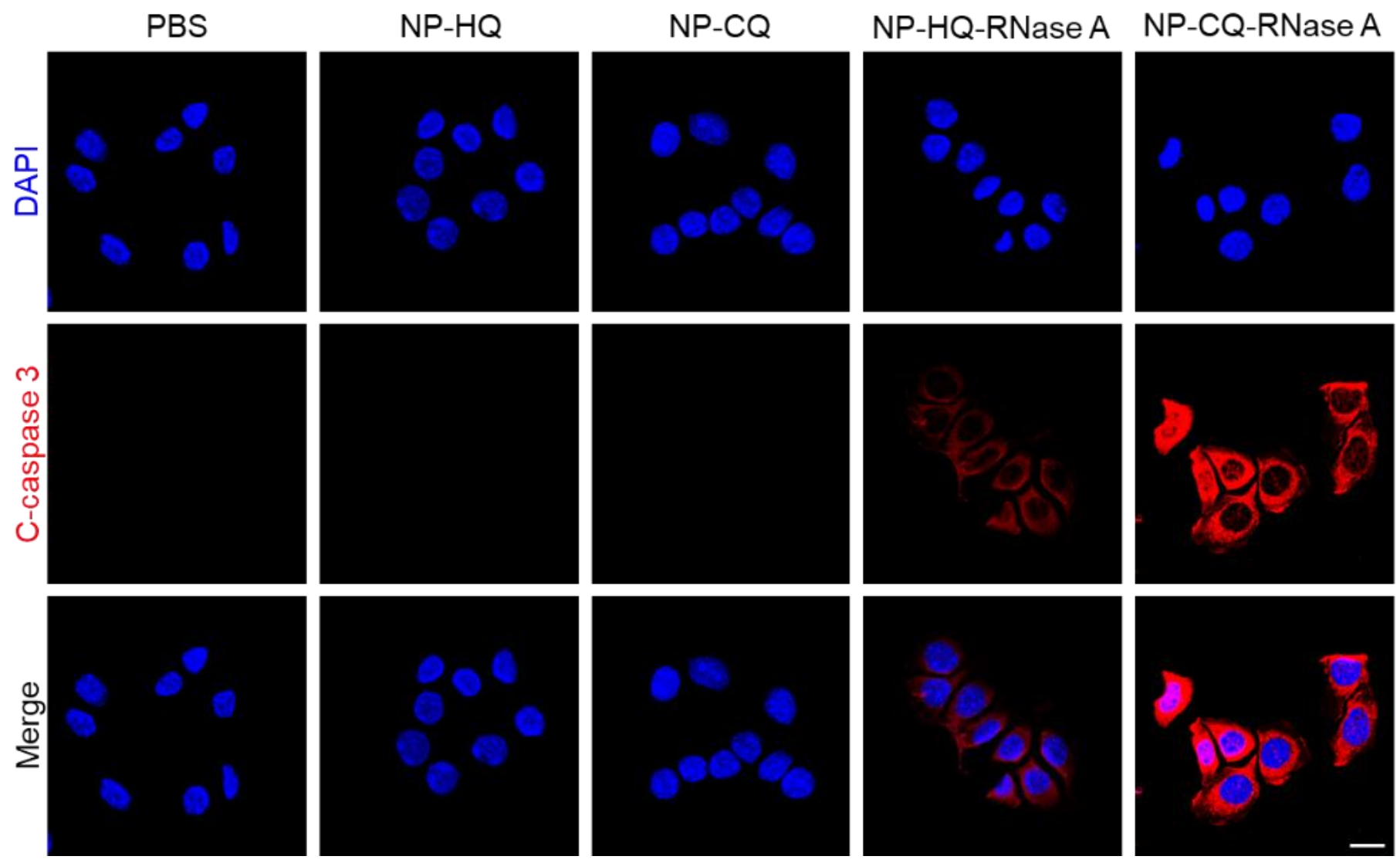


**Figure S37.** Immunofluorescence detection of C-caspase 3 in 4T1 cells following 24 h treatments (10 μg/mL RNase A). Scale bar = 10 μm.

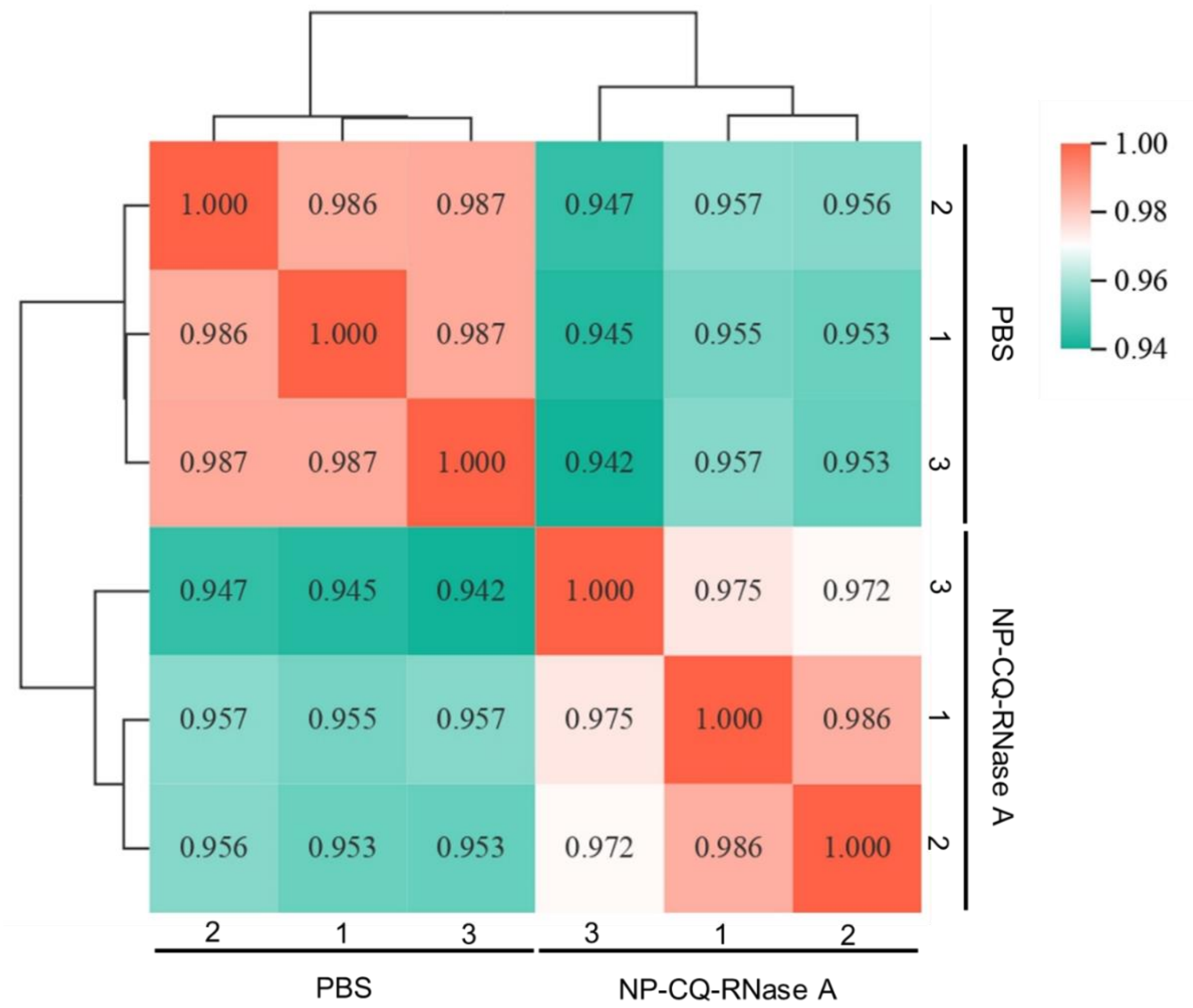


**Figure S38.** Heat map diagram of Pearson correlation coefficients between each sample.

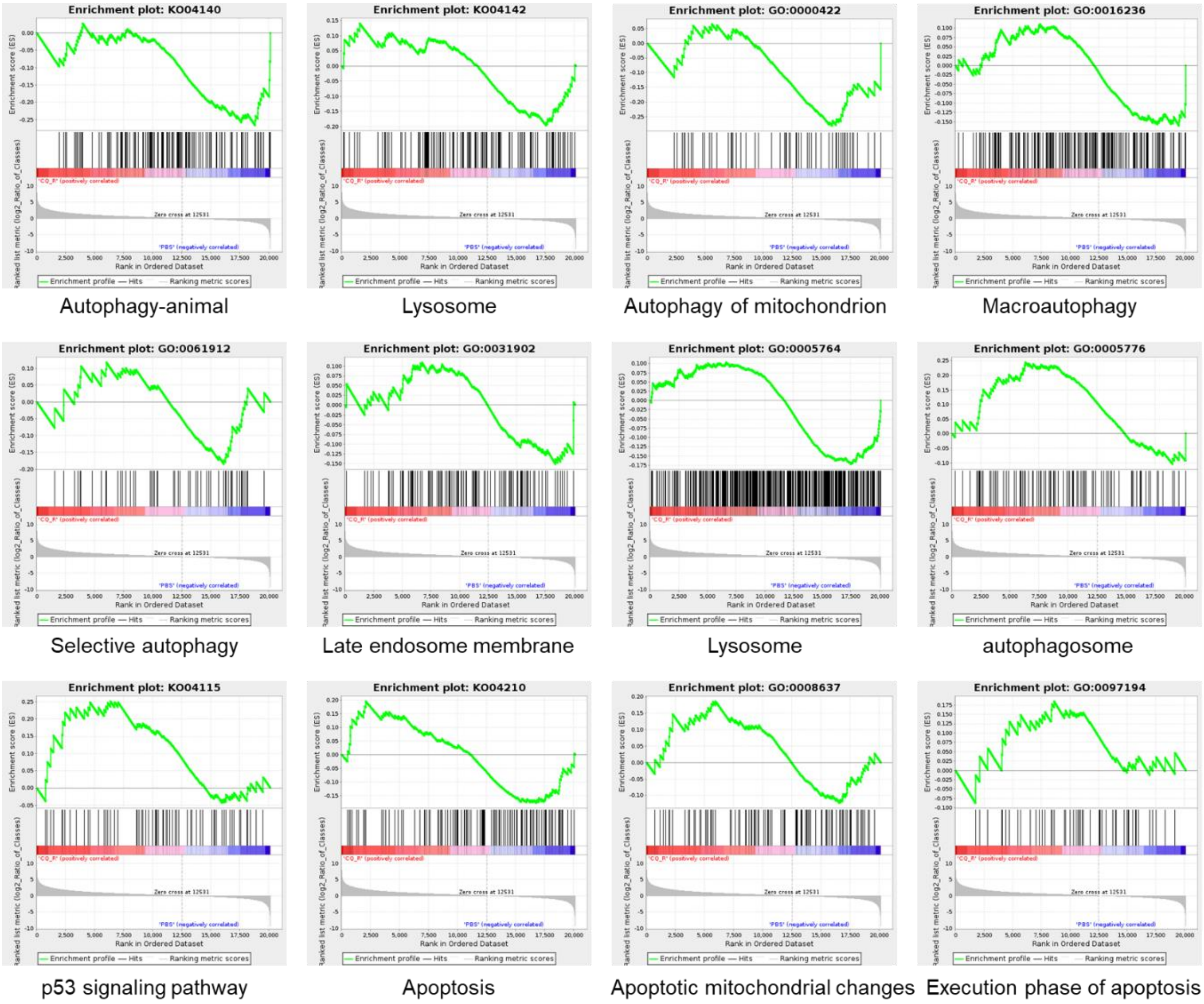


**Figure S39.** GSEA for the depicted signaling pathway.

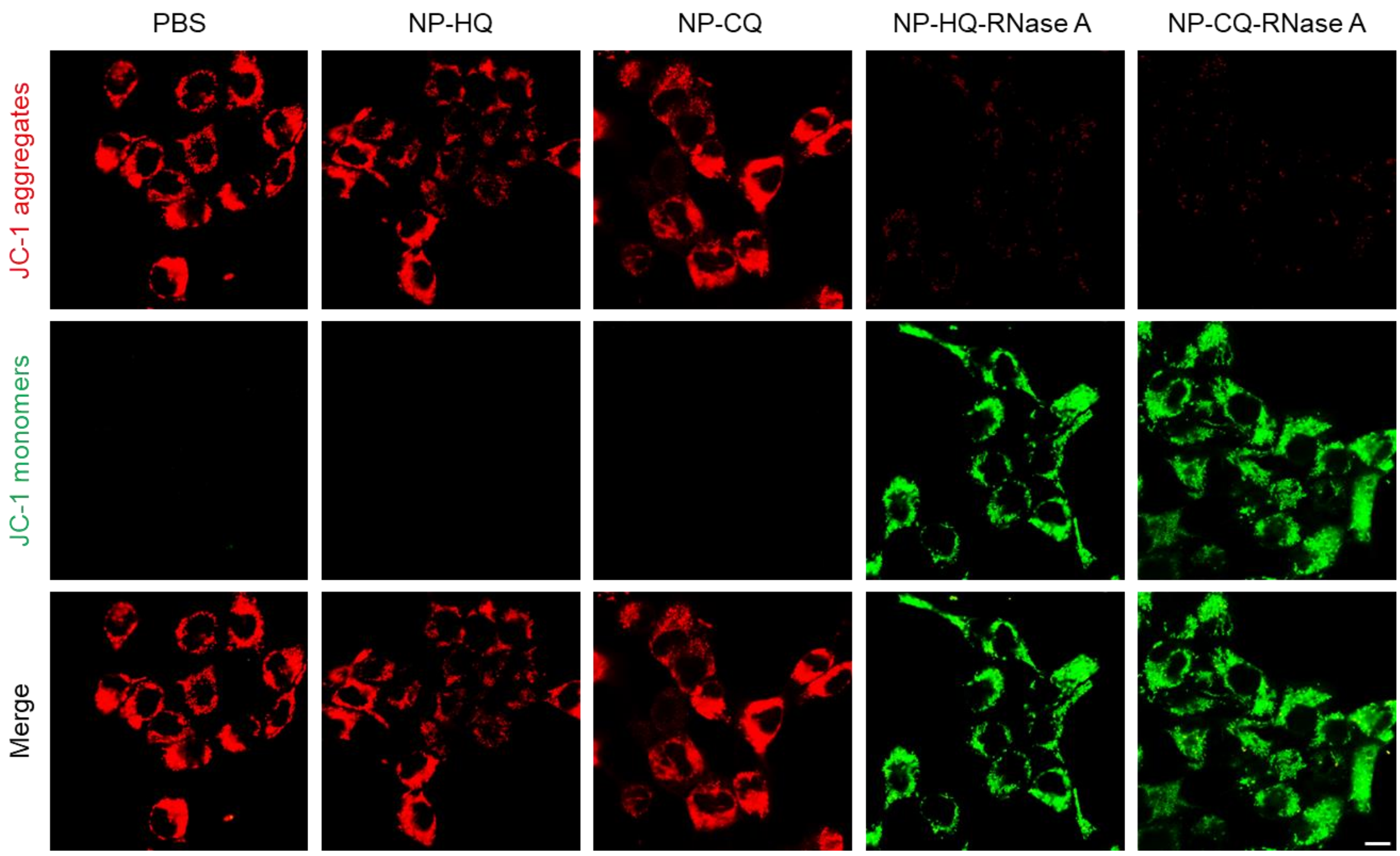


**Figure S40.** Assessment of ΔΨm using JC-1 staining after 24 h treatments (10 μg/mL RNase A). Scale bar = 10 μm.

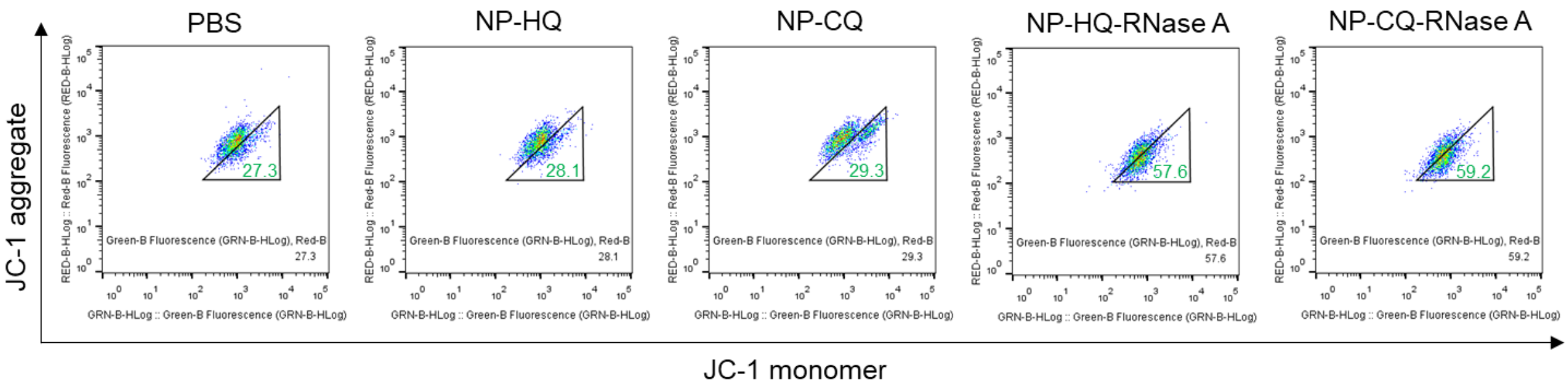


**Figure S41.** JC-1 staining of 4T1 cells after treatment with indicated formulations (10 µg/mL RNase A) for 24 h detected by flow cytometry.

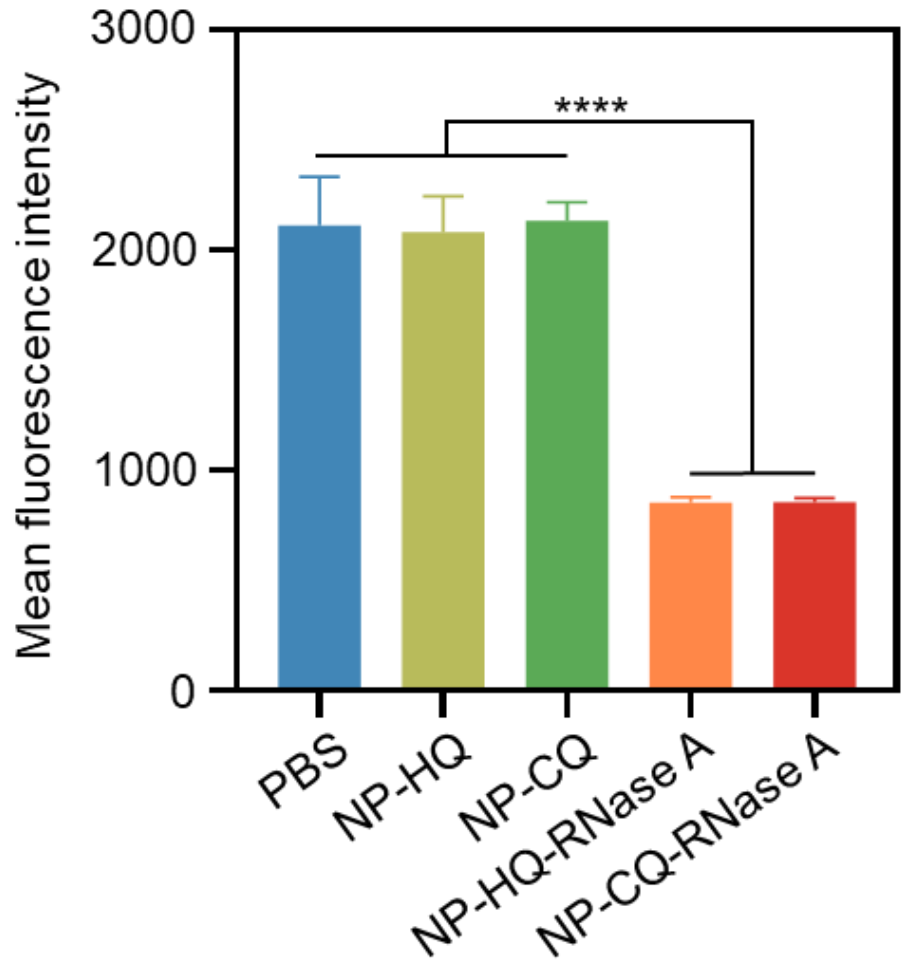


**Figure S42.** Rhodamine 123 staining of 4T1 cells after treatment with indicated formulations (10 μg/mL RNase A) for 24 h detected by flow cytometry. ****$p < 0.001$. Data are shown as mean ± SD (n = 3).

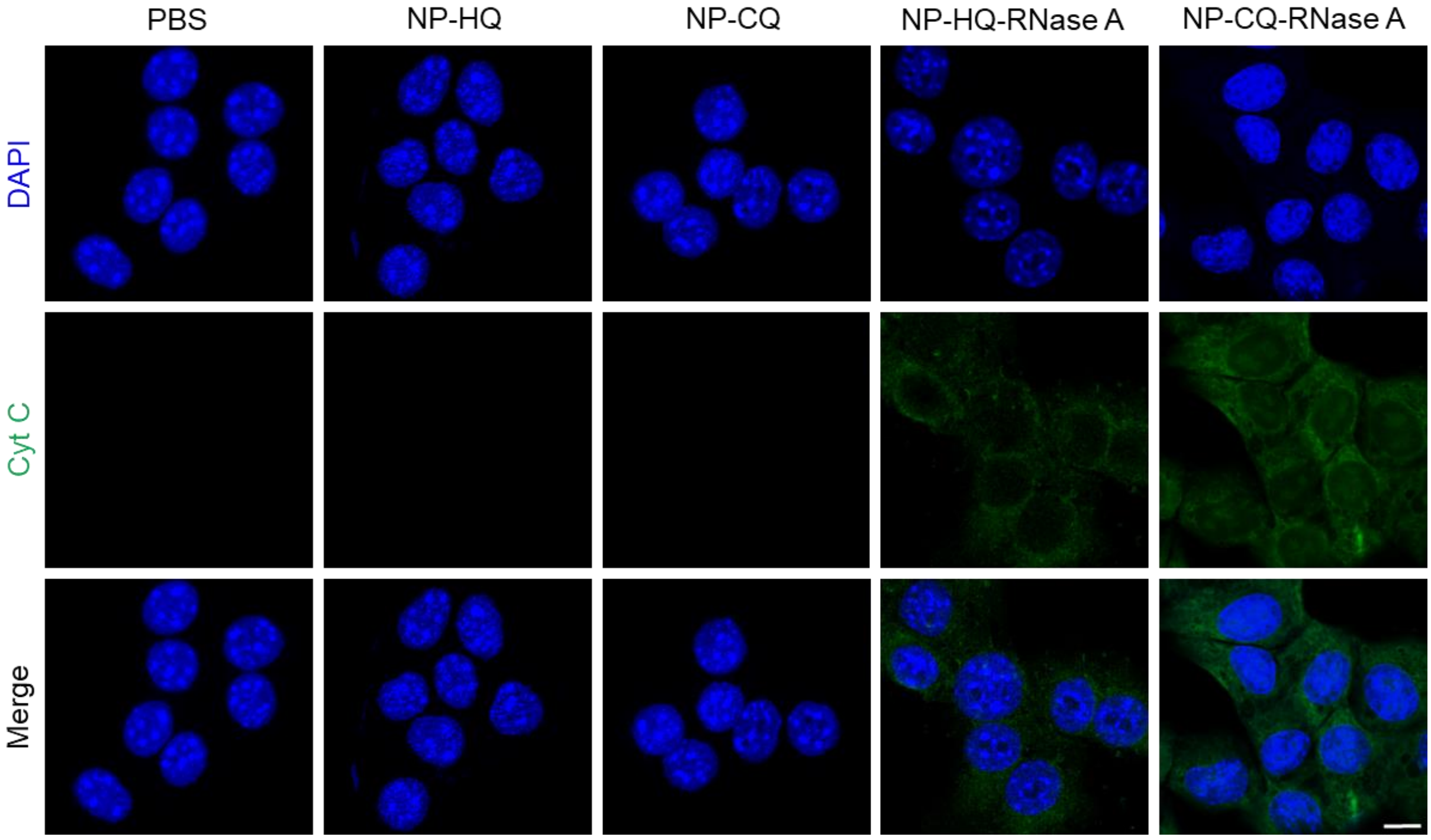


**Figure S43.** Representative immunofluorescence micrographs showing Cyt C localization in 4T1 cells subjected to indicated formulations (10 μg/mL RNase A) for 24 h. Scale bar = 10 μm.

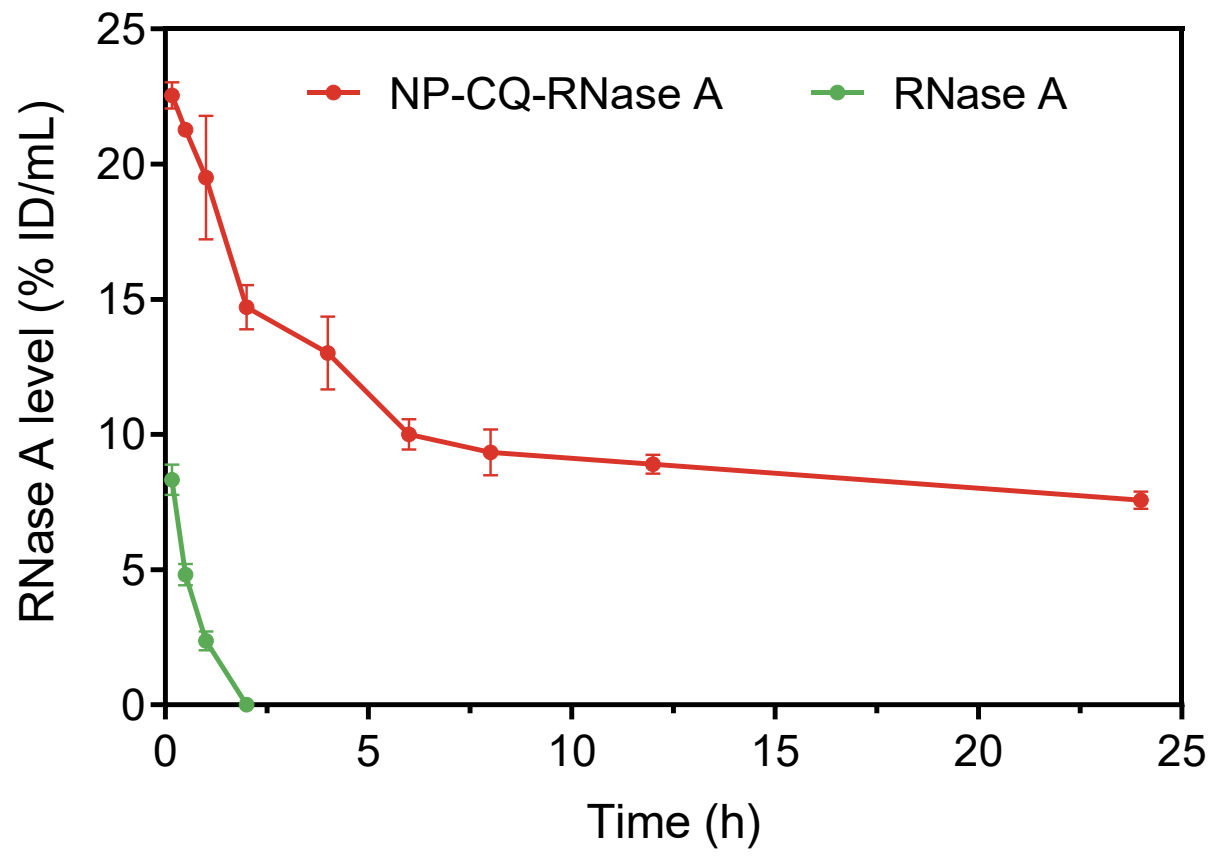


**Figure S44.** Pharmacokinetics of NP-CQ-RNase A-Cy5 and RNase A-Cy5 following intravenous injection. Data are shown as mean ± SD (n = 3).

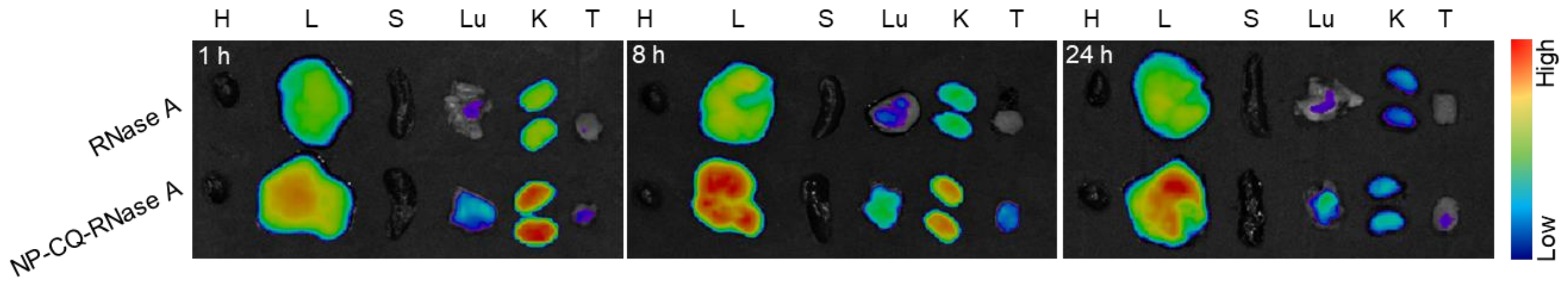


**Figure S45.** *Ex vivo* fluorescence imaging of the excised tumors and major organs of RNase-Cy5 and NP-CQ-RNase A-Cy5 treated 4T1 tumor-bearing mice at 1, 8 and 24 h post-injection (H: heart; L: liver; S: spleen; Lu: lung; K: kidney; T: tumor).

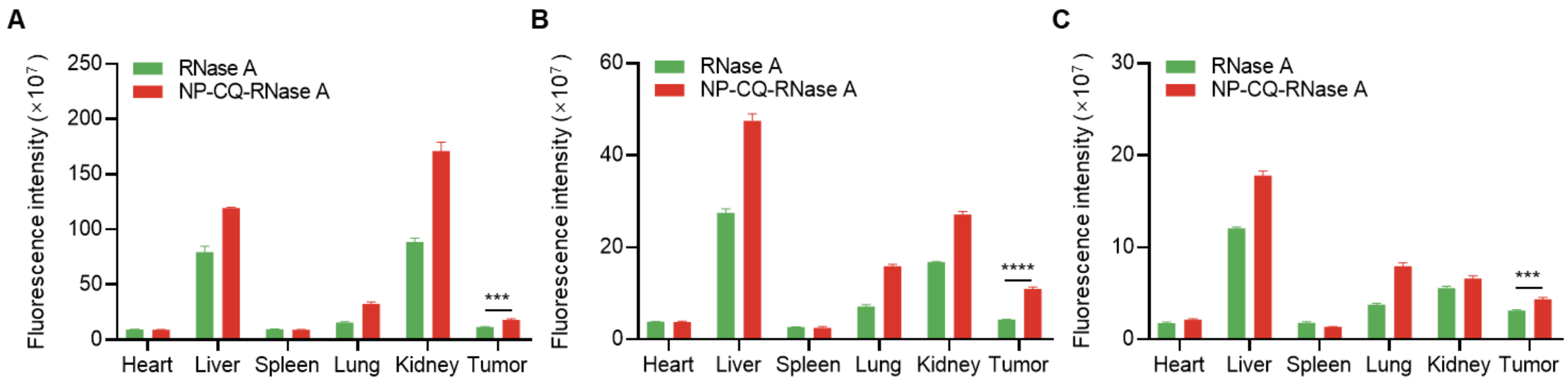


**Figure S46.** Biodistribution of RNase A-Cy5 in the tumors and major organs of RNase A-Cy5 and NP-CQ-RNase A-Cy5 treated 4T1 tumor-bearing mice at 1 h (A), 8 h (B) and 24 h (C) post-injection. ***$p < 0.001$, ****$p < 0.0001$. Data are shown as mean ± SD (n = 3).

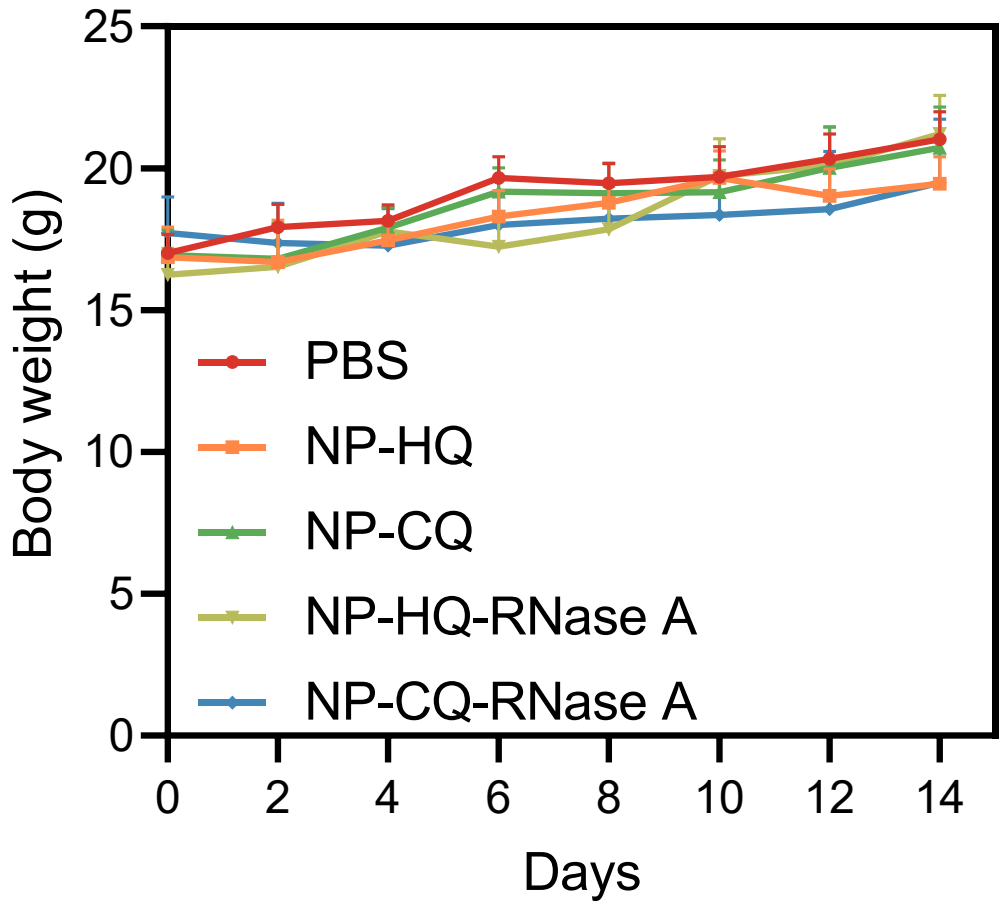


**Figure S47.** Body weight changes of 4T1 tumor-bearing mice during treatment. Data are shown as mean ± SD (n = 5).

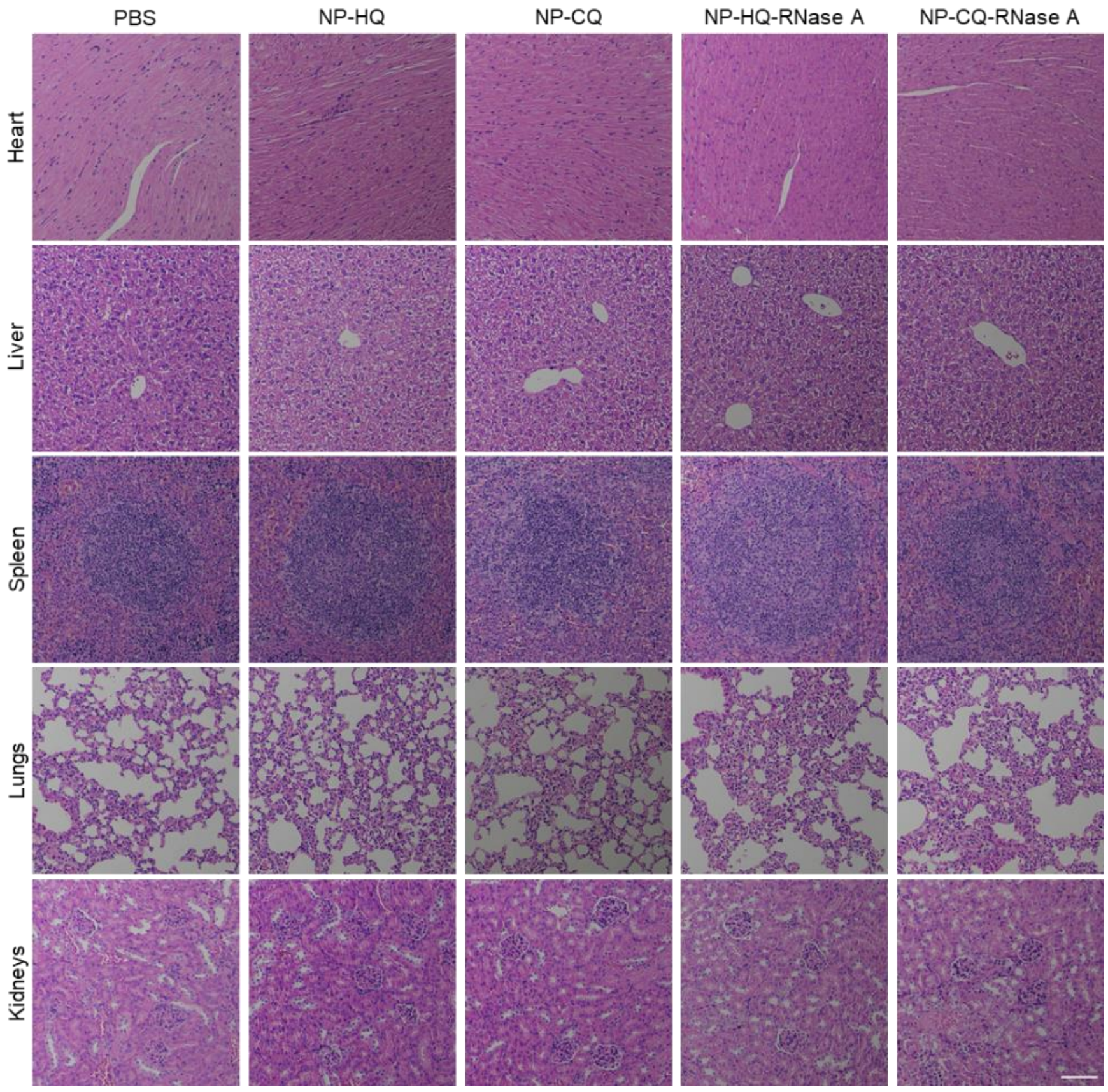


**Figure S48.** H&E staining of major organs from the 4T1 tumor-bearing mice at the end of treatment. Scale bar = 100 μm.

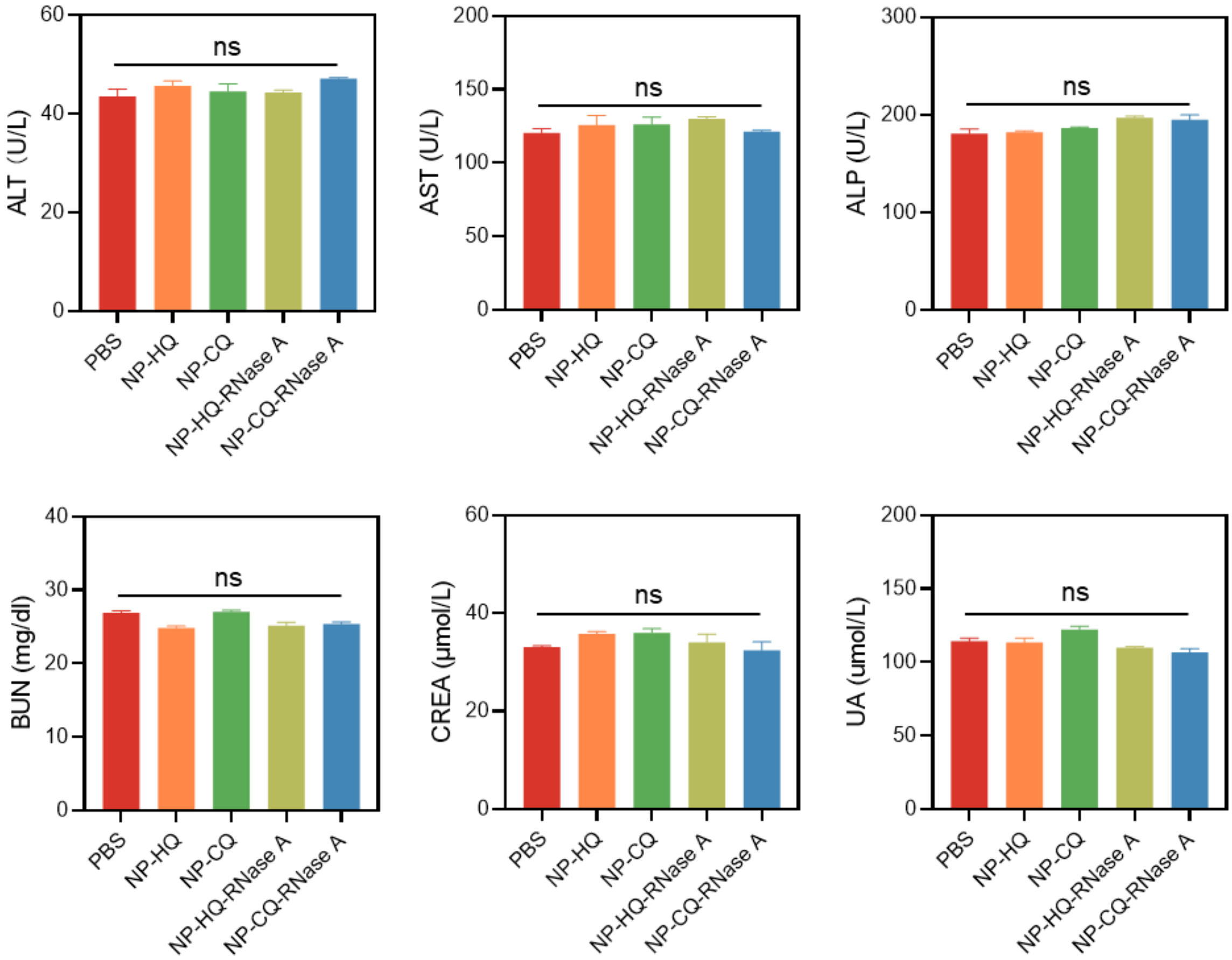


**Figure S49**. Quantitative analyses of liver (ALT, AST, ALP) and kidney (BUN, CREA, UA) function at the end of treatment. Data are shown as mean ± SD (n = 3), ns: not significant.

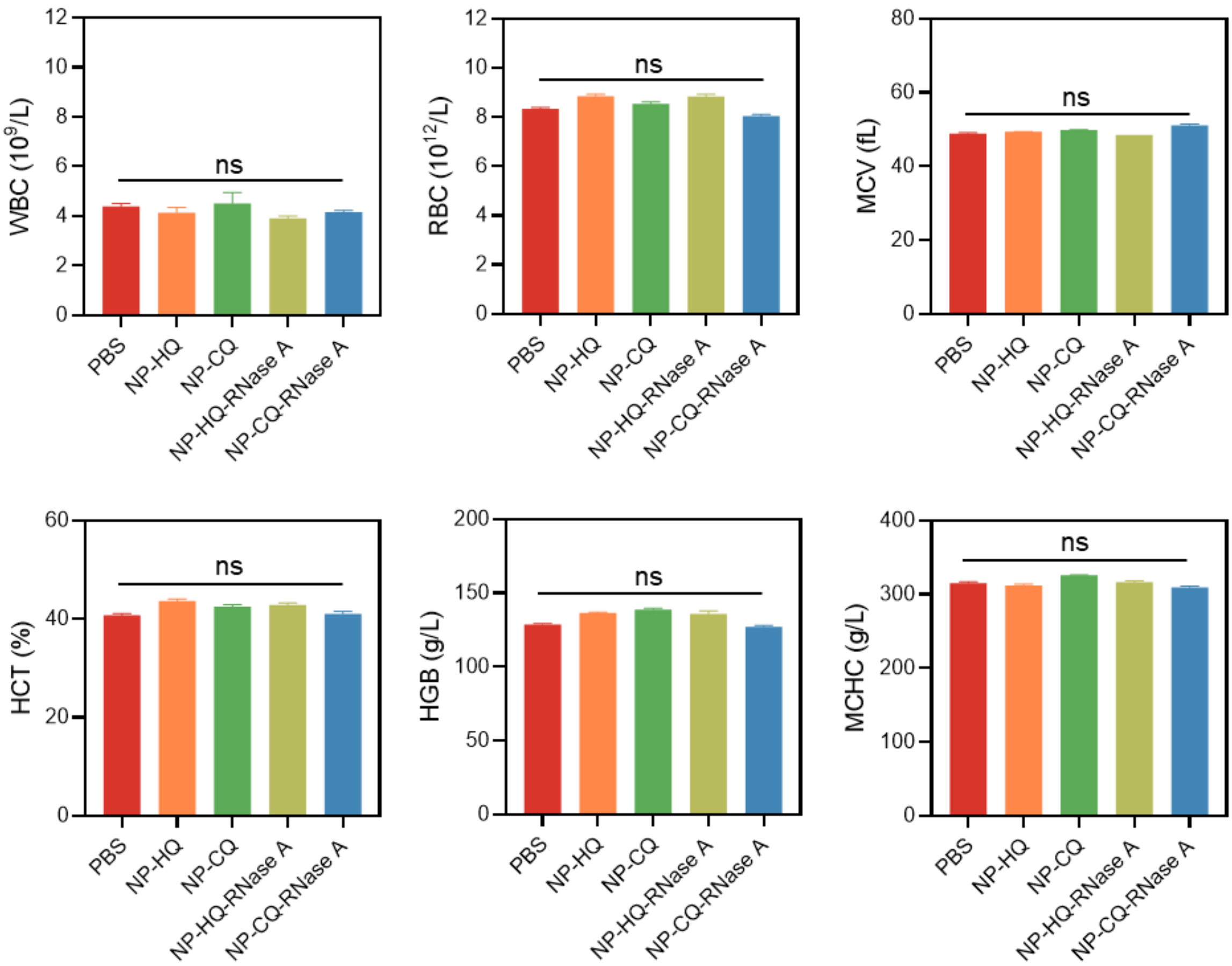


**Figure S50**. Blood routine tests of indicated groups at the end of treatment. Data are shown as mean ± SD (n = 3), ns: not significant.

**Table S1.** The DLC and DLE of various proteins in indicated nanoparticles.

| Nanoparticles | NP-HQ-GFP | NP-CQ-GFP | NP-HQ-Gal | NP-CQ-Gal | NP-HQ-HRP | NP-CQ-HRP |
|---|---|---|---|---|---|---|
| DLC (%) | 22.2 | 23.3 | 18.6 | 20.1 | 26.5 | 27 |
| DLE (%) | 96.2 | 97.5 | 94.4 | 95.1 | 97.6 | 97.9 |

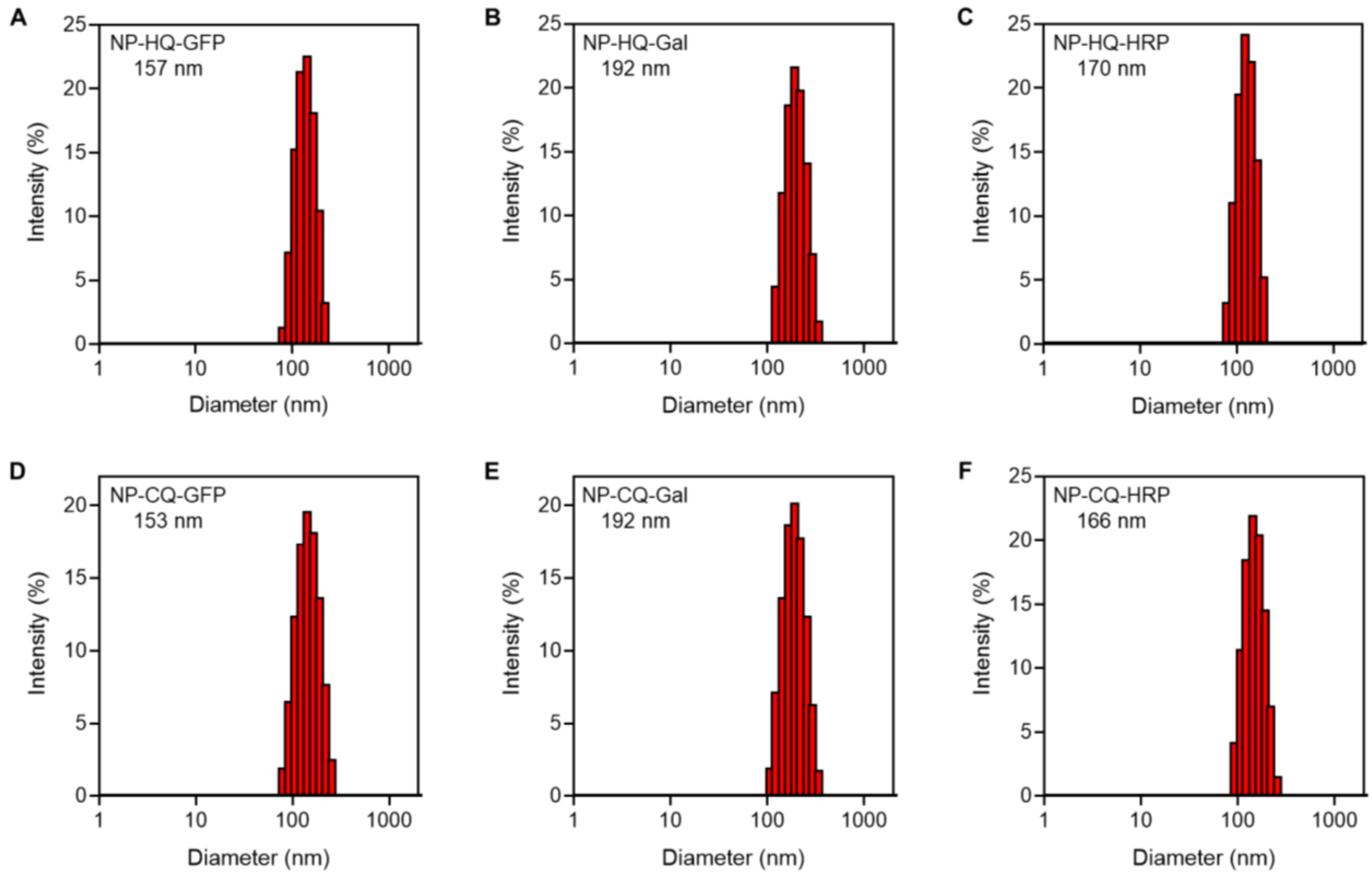


**Figure S51.** Particle size of NP-HQ-GFP (A), NP-HQ-Gal (B), NP-HQ-HRP (C), NP-CQ-GFP (D), NP-CQ-Gal (E) and NP-CQ-HRP (F) in PBS (pH 7.4) determined by DLS.

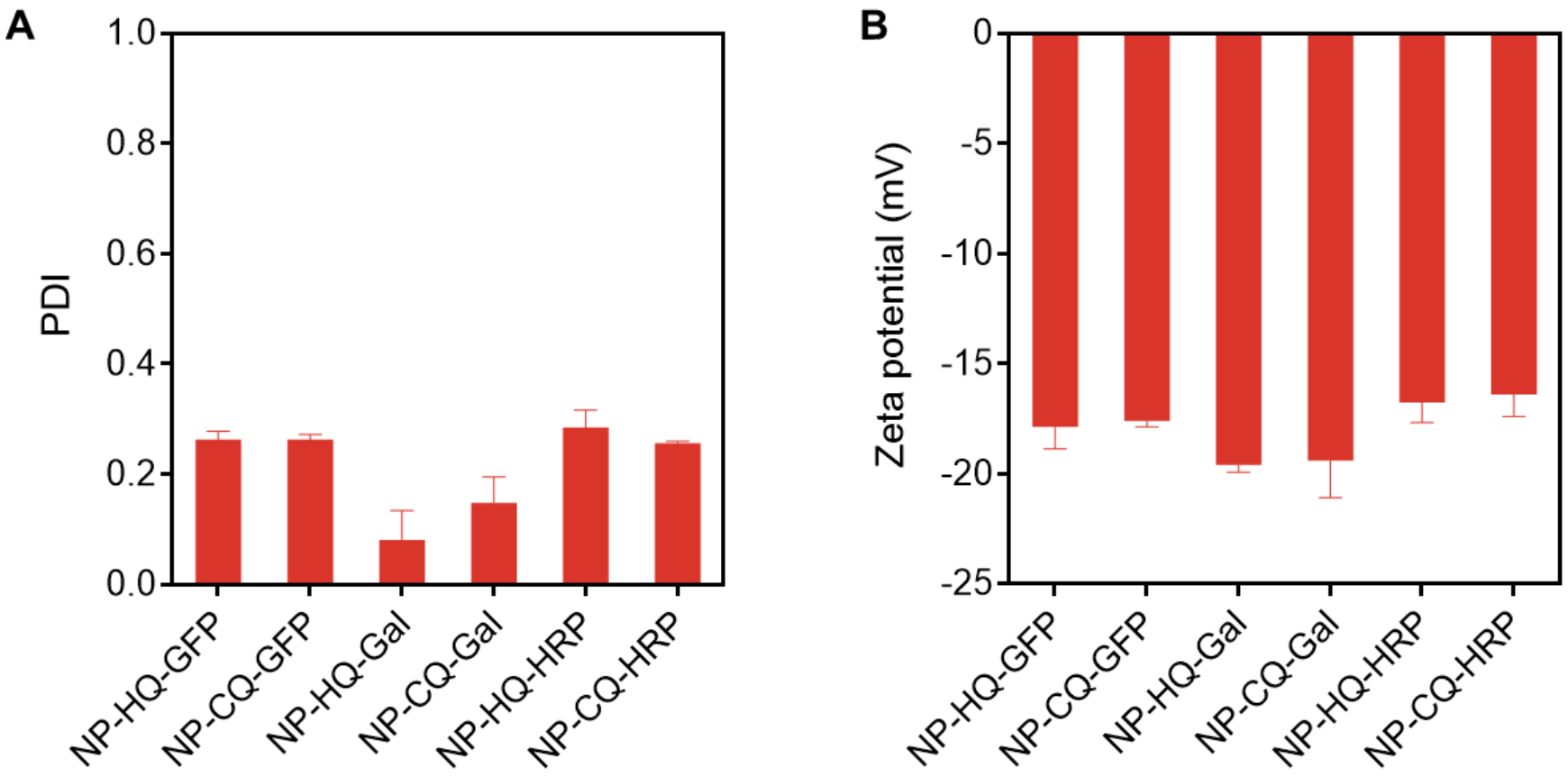


**Figure S52.** (A) PDI of NP-HQ-GFP, NP-CQ-GFP, NP-HQ-Gal, NP-CQ-Gal, NP-HQ-HRP and NP-CQ-HRP in PBS (pH 7.4) determined by DLS. (B) Zeta potential of NP-HQ-GFP, NP-CQ-GFP, NP-HQ-Gal, NP-CQ-Gal, NP-HQ-HRP and NP-CQ-HRP in PBS (pH 7.4) determined by DLS. Data are shown as mean ± SD (n = 3).

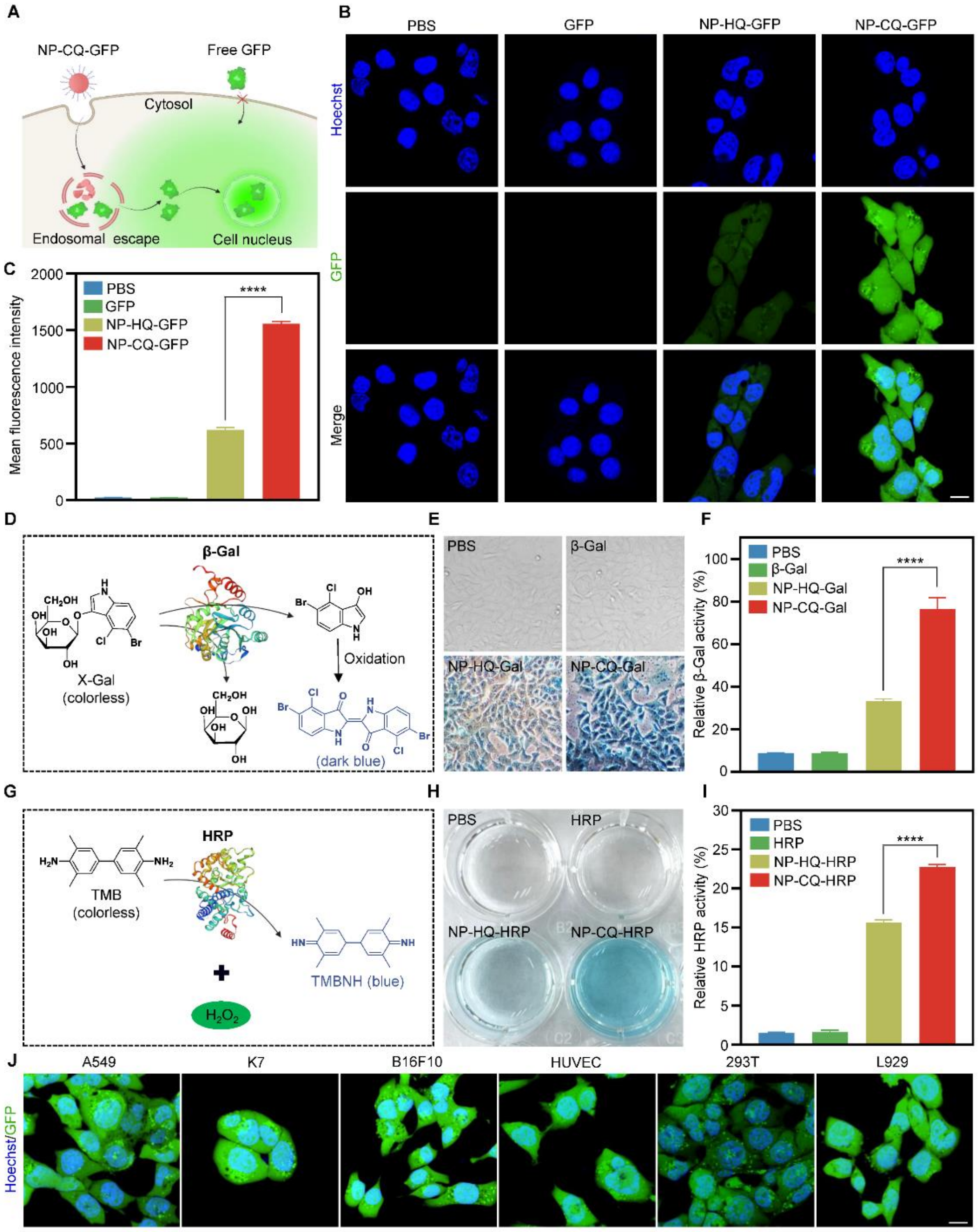


**Figure S53.** NP-CQ-mediated cytosolic transport of GFP, β-Gal, and HRP. (A) Diagram depicting the intracellular delivery mechanism of GFP via NP-CQ-GFP. (B) Fluorescence micrographs of 4T1 cells following 24 h incubation with GFP, NP-HQ-GFP, or NP-CQ-GFP (1 μM GFP) at 37°C. Nuclei were visualized with Hoechst 33342 (1 μg/mL, 10 min). Scale bar = 10 μm. (C) Flow cytometric quantification of mean fluorescence intensities in 4T1 cells under the treatments indicated in (B). (D) Schematic of the enzymatic reaction where β-Gal converts colorless X-Gal into a blue precipitate. (E) X-Gal staining results in 4T1 cells after 24 h exposure to different formulations (1 μM β-Gal) at 37°C. Scale bar = 50 μm. (F) Quantified intracellular β-Gal activity levels post-treatment with various formulations (4 μg/mL β-Gal) for 24 h. (G) Illustration of the

HRP-catalyzed oxidation of colorless TMB to a blue product. (H) TMB staining in 4T1 cells treated with the indicated formulations (4 µg/mL HRP) for 24 h. (I) Measured intracellular HRP activity following treatment with different formulations (4 µg/mL HRP) for 24 h. (J) Fluorescence visualization of multiple cell lines after 24 h incubation with NP-CQ-GFP (1 µM GFP). Nuclei were labeled with Hoechst 33342. Scale bar = 10 µm. ****$p < 0.0001$. All values represent mean ± SD ($n = 3$).

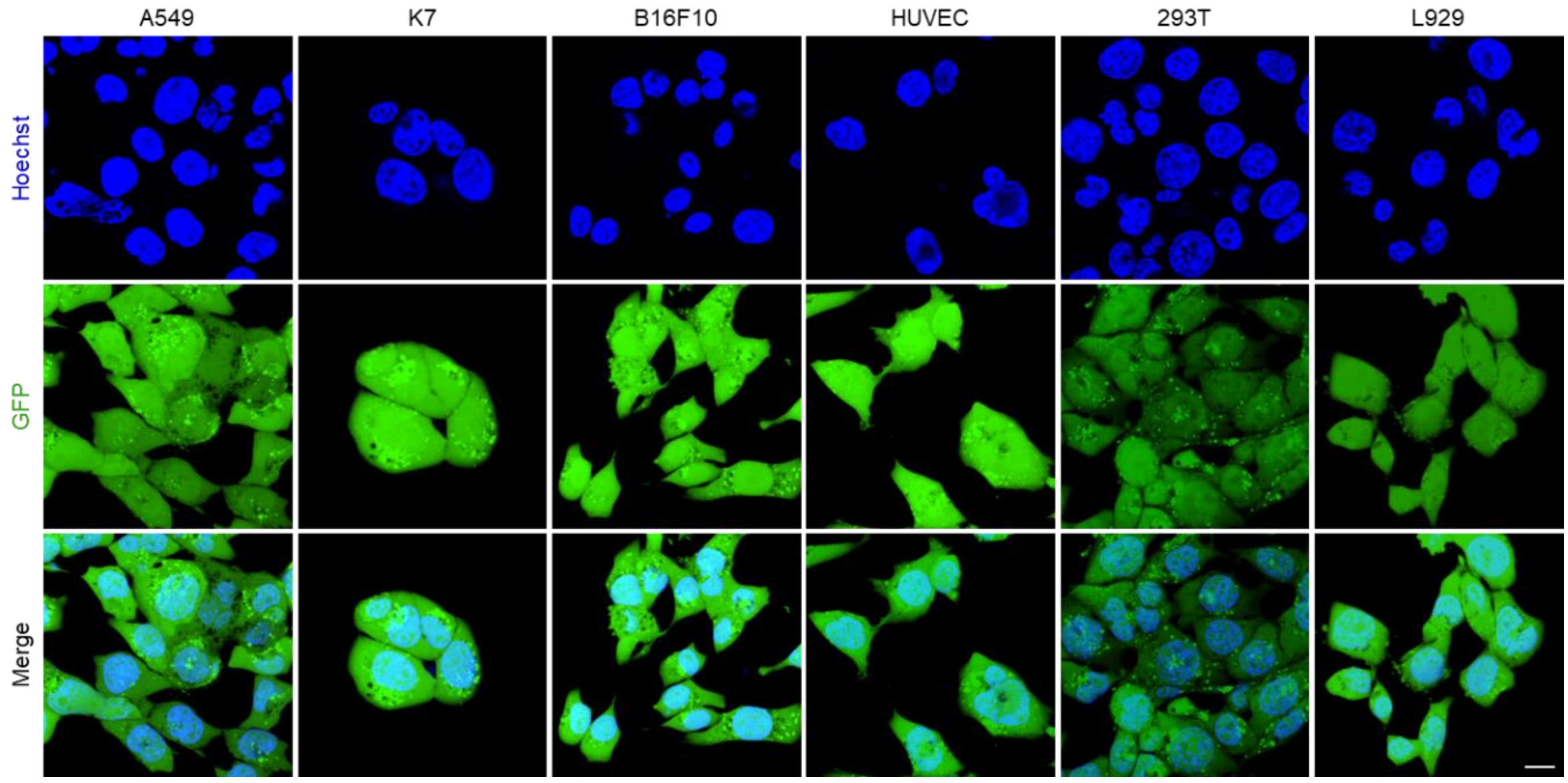


**Figure S54.** Fluorescence visualization of multiple cell lines after 24 h incubation with NP-CQ-GFP (1 μM GFP). Nuclei were labeled with Hoechst 33342. Scale bar = 10 μm.

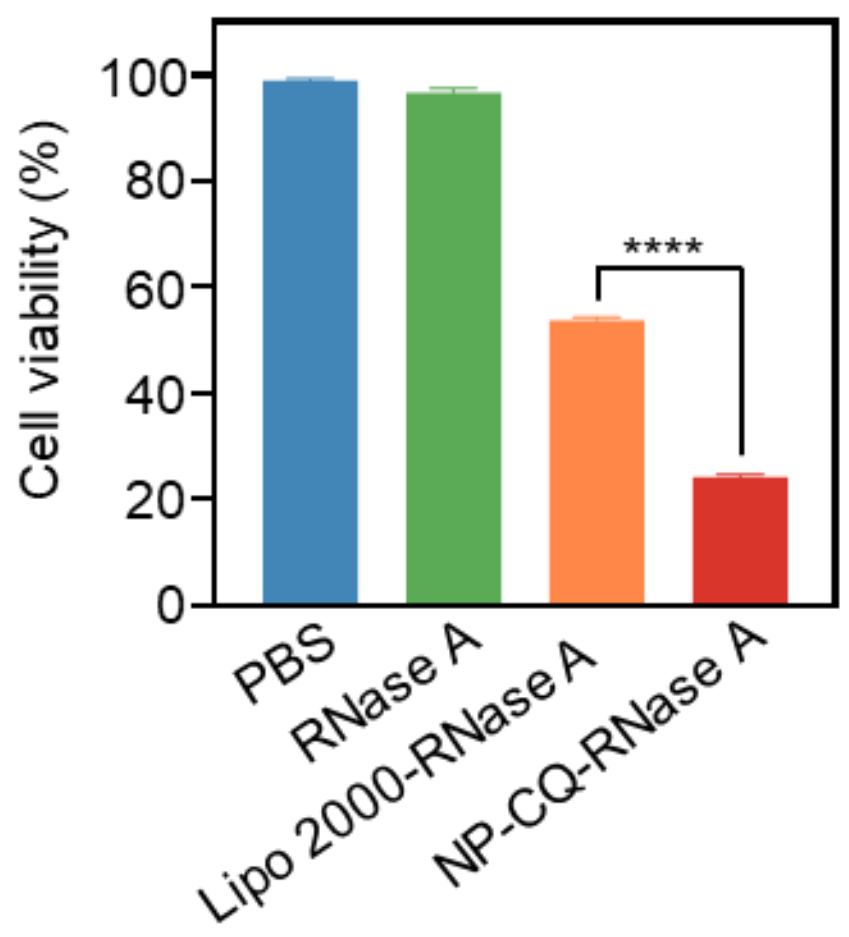


**Figure S55.** Cell viability of 4T1 cells following treatment with RNase A, Lipo 2000-RNase A, and NP-CQ-RNase A (0.4 μM RNase A) for 72 h at 37 °C, as determined by an MTT assay. ****$p < 0.001$. Data are presented as mean ± SD (n = 3). Lipo 2000-RNase A complexes were prepared by dissolving 4 μg of RNase A in 90 μL of Opti-MEM medium, followed by the addition of 10 μL of Lipofectamine 2000 (Lipo 2000). The mixture was gently vortexed and incubated at RT for 15 min to allow complex formation prior to cell treatment.
